# Potent Phytotoxic Harziane Diterpenes from a Soft Coral-Derived Strain of the Fungus *Trichoderma harzianum* XS-20090075

**DOI:** 10.1038/s41598-019-49778-7

**Published:** 2019-09-16

**Authors:** Dong-Lin Zhao, Lu-Jia Yang, Ting Shi, Chao-Yi Wang, Chang-Lun Shao, Chang-Yun Wang

**Affiliations:** 1 Key Laboratory of Marine Drugs, The Ministry of Education of China, School of Medicine and Pharmacy, Ocean University of China, Qingdao, 266003 People’s Republic of China; 2 Marine Agricultural Research Center, Tobacco Research Institute of Chinese Academy of Agricultural Sciences, Qingdao, 266101 People’s Republic of China; 3 Laboratory for Marine Drugs and Bioproducts, Qingdao National Laboratory for Marine Science and Technology, Qingdao, 266237 People’s Republic of China; 4 Institute of Evolution & Marine Biodiversity, Ocean University of China, Qingdao, 266003 People’s Republic of China

**Keywords:** Natural products, Small molecules

## Abstract

Two new harziane diterpene lactones, possessing a 6/5/7/5-fused carbocyclic core containing a lactone ring system, harzianelactones A and B (**1** and **2**), and five new harziane diterpenes, harzianones A–D (**3**–**6**) and harziane (**7**), were isolated from the soft coral-derived fungus *Trichoderma harzianum* XS-20090075. Their structures were determined by extensive NMR spectroscopic data, ECD and OR calculations, as well as X-ray diffraction. The isolated compounds exhibited potent phytotoxicity against seedling growth of amaranth and lettuce. Harziane diterpenes were rarely reported for their remarkably bioactivities, and it was the first report to study the phytotoxicity of harziane diterpenes, which provide a new application of such compounds in agriculture for future research.

## Introduction

Increasing concerns for the management of weeds have been caused by scientists, as they can bring out greater reduction in crop yields than plant diseases and pests^1^. Nowadays more than half of the pesticides used are herbicides^1,2^. With the increasing attention to food safety and environmental protection, it is desiderate to develop new types of bio-herbicides with high efficiency and low toxicity.

*Trichoderma* spp. are one of the most commonly disseminated fungi in nature, and are distributed around the world ranging from the tundra to the tropics. They have been widely used as biocontrol agents (*T. harzianum, T. atroviride*, and *T. asperellum*), and commercially marketed as biopesticides, due to their capacity to parasitize in other fungi and to compete with deleterious plant microorganisms^3^. However, there are few *Trichoderma* spp. products sold in the commercial market, and limited studies are focused on the phtotoxicity of compounds from *Trichoderma* spp^4^.

Marine fungi have gained more and more attention over the past decades, as the recognition that they are a quite diverse group and an excellent source of natural products, possessing prominent bioactivities, including antibacterial, antifungal, antiviral, anti-inflammatory, antitumor, and insecticidal^5^. Marine-derived *Trichoderma* spp. have been reported to represent a potential source for producing compounds with novel structures and remarkable bioactivities, such as trichodermamides A and B^6^, dithioaspergillazine A^7^, tandyukisins E and F^8^, as well as harzianone^9^. Therefore, it has huge potential to find new phytotoxic compounds from marine-derived *Trichoderma* spp.

**Figure 1:**
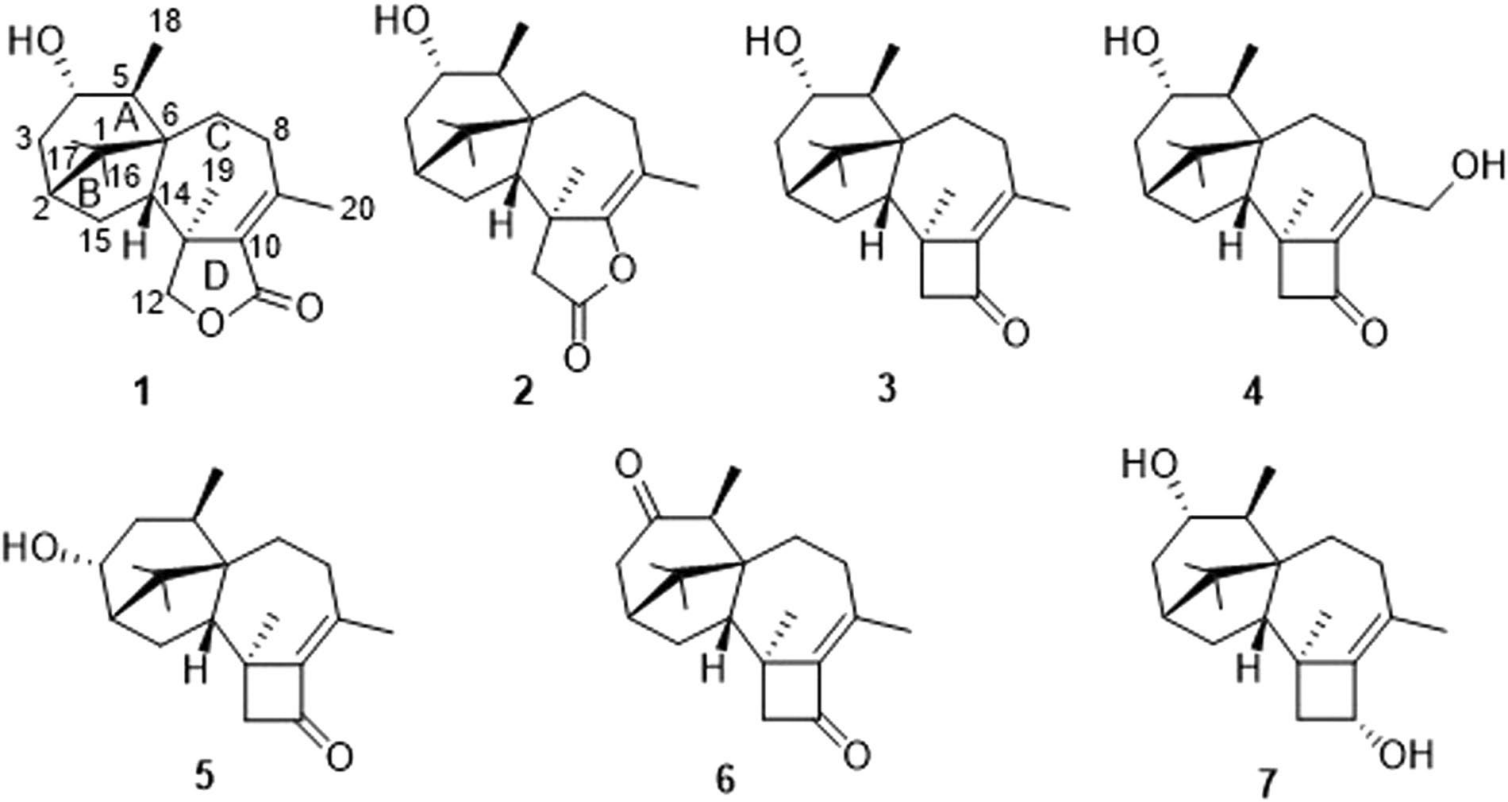
Chemical structures of harzianelactones A and B (1 and 2), harzianones A–D (3–6), and harziane (7).

During our efforts to find novel bioactive compounds from coral-derived fungi in the South China Sea^10–13^, a *T. harzianum* XS-20090075 strain attracted our attention because the finger-print for the extract of the fungal culture on HPLC showed abundant peaks with interesting UV absorption spectra at around 250 nm, and the fungal extracts showed obvious phytotoxicity. Further chemical examination on the EtOAc extract resulted in the discovery of two new harziane diterpene lactones, harzianelactones A and B (**1** and **2**), and five new harziane diterpenes, harzianones A–D (**3**–**6**), and harziane (**7**) (Fig. 1). Herein, we describe the isolation, structure elucidation, and phytotoxicity of these harziane diterpenes.

## Results and Discussion

**Figure 2:**
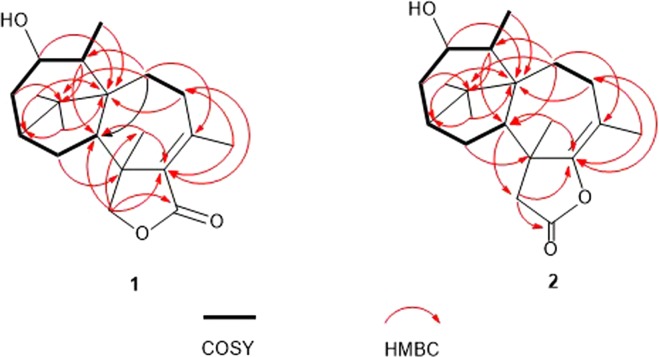
COSY and key HMBC correlations of 1 and 2.

Harzianelactone A (**1**) was obtained as a colorless oil with the molecular formula of C_20_H_30_O_3_ by HRESIMS, requiring six degrees of unsaturation. The ^1^H NMR spectrum (Table 1) showed three protons on oxygenated carbons at *δ*_H_ 3.89 (d, *J* = 9.0 Hz), 3.80 (d, *J* = 8.0 Hz), and 3.73 (d, *J* = 8.0 Hz), four methyl singlets at *δ*_H_ 2.33 (s), 1.43 (s), 0.85(s), and 0.84 (s), as well as one methyl doublet at *δ*_H_ 1.15 (d, *J* = 8.0 Hz). The ^13^C NMR (Table 2) and DEPT spectra in combination with HMQC data revealed one lactone carbonyl (*δ*_C_ 171.3), one oxymethylene carbon (*δ*_C_ 78.4), one oxymethine carbon (*δ*_C_ 73.7), four methylenes (*δ*_C_ 39.8, 32.1, 28.2, and 28.0), three methines (*δ*_C_ 50.5, 41.3, and 40.0), five methyl groups (*δ*_C_ 24.9, 22.9, 22.6, 21.2, 20.7), and five nonprotonated carbons (*δ*_C_ 155.9, 131.0, 49.7, 46.3, 44.9) including two quaternary olefinic ones. The aforementioned data corresponded to two degrees of unsaturation, and the remaining four degrees of unsaturation suggested the existence of four rings. The planar structure of **1** was elucidated on the basis of COSY and HMBC experiments (Fig. 2). The spin systems of H-14/H-15/H-2/H-3/H-4/H-5/H-18 in the COSY cross peaks and the correlations from H-3 to C-1, C-5, and C-15, from H-4 to C-2, C-6 and C-18, from H-5 to C-1 and C-14, from H-16 and H-17 to C-2 and C-6, and from H-18 to C-6 in the HMBC spectrum, led to the construction of a five-membered ring B and a six-membered ring A with a hydroxy and a methyl group anchored to C-4 and C-5, respectively. The seven-membered ring C with two methyl groups connected to C-9 and C-13 was further constructed according to the HMBC correlations from H-7 to C-9 and C-14, from H-8 to C-6 and C-10, from H-19 to C-10 and C-14, and from H-20 to C-8 and C-10. The lactone carbonyl (*δ*_C_ 171.3), in addition to the HMBC correlations from H-12 to C-10, C-11, C-14, and C-19, and from H-15 to C-13 indicated the existence of a five-membered lactone ring D connected to ring C. Finally, the connection of ring B and ring C was confirmed by the HMBC correlations from H-7 and H-14 to C-1, from H-7 to C-5, and from H-15 to C-13. Therefore, the planar structure of **1** was determined.

**Table 1:** 1H NMR Data of **1**–**7** (500 MHz, CDCl_3_, *δ* in ppm, *J* in Hz).

Position	1	2	3	4	5	6	7
2	1.66, m	1.65, m	1.53–1.58, m	1.59–1.65, m	1.89–2.01, m	1.73, m	1.58–1.60, m
3	2.39, ddd (13.5, 9.0, 4.0)	2.30–2.41, m	2.27–2.36, m	2.38, ddd (14.5, 8.5, 4.0)	2.73, brd (18.0)	4.27, m	2.38, ddd (14.0, 9.0, 4.5)
	1.43–1.45, m	1.44–1.47, m	1.43, d (15.0)	1.48, d (14.5)	2.28–2.41, m		1.43, d (14.0)
4	3.89, d (9.0)	3.86, d (8.5)	3.82, d (8.5)	3.87, d (8.5)		1.62–1.82, m	3.83, d (9.0)
5	2.55, q (8.0)	2.49, q (8.0)	2.54, q (8.0)	2.57, q (8.0)	3.13, q (8.0)	2.54, q (8.0)	2.49, brq (8.0)
7	1.83, dd (13.0, 7.0)	1.78, dd (13.0, 6.5)	1.76, dd (13.0, 7.0)	1.85, dd (13.0, 7.0)	2.28–2.41, m	1.77–1.82, m	1.75–1.82, m
	1.28, t (13.0)	1.27, t (13.0)	1.15, t (13.0)	1.18, t (13.0)	1.33, t (13.5)	1.28, t (13.0)	1.07, t (13.0)
8	2.48, t (14.5)	2.33, t (14.0)	2.27–2.36, m	2.30, t (13.0)	2.28–2.41, m	2.36, t (14.0)	2.27, t (14.0)
	2.10, dd (14.5, 7.0)	1.87, dd (14.0, 6.5)	1.82–1.90, m	1.98, dd (13.0, 7.0)	1.89–2.01, m	1.93, dd (14.0, 5.0)	1.75–1.82, m
11							4.66, d (7.0)
12	3.80, d (8.0)	2.46, d (17.0)	2.46, d (16.5)	2.58, d (17.0)	2.51, d (16.5)	2.55, d (16.0)	1.88–1.93, m
	3.73, d (8.0)	2.33, d (17.0)	2.33, d (16.5)	2.47, d (17.0)	2.38, d (16.5)	2.39, d (16.0)	1.58–1.60, m
14	2.04, t (10.5)	2.15, t (11.0)	2.00, t (10.5)	2.05, t (10.5)	2.28–2.41, m	2.19, t (10.0)	1.91, t (10.5)
15	1.79–1.86, m	1.82–1.89, m	1.82–1.90, m	1.90–1.94, m	1.17–1.23, m	1.62–1.69, m	1.82–1.86, m
	1.49–1.54, m	1.57, t (12.0)	1.53–1.58, m	1.59–1.65, m			1.37–1.45, m
16	0.84, s	0.85, s	0.79, s	0.83, s	1.13, s	0.89, s	0.82, s
17	0.85, s	0.86, s	0.80, s	0.84, s	1.01, s	1.04, s	0.83, s
18	1.15, d (8.0)	1.14, d (8.0)	1.09, d (8.0)	1.14, d (8.0)	1.23, d (8.0)	1.05, d (8.0)	1.13, d (8.0)
19	1.43, s	1.43, s	1.47, s	1.56, s	1.33, s	1.48, s	1.58, s
20	2.33, s	1.75, s	2.02, s	4.36, d (18.5)	2.09, s	2.08, s	1.72, s
				4.24, d (18.5)

**Table 2:** 13C NMR Data of 1–7 (125 MHz, CDCl_3_, *δ* in ppm).

Position	1	2	3	4	5	6	7
1	44.9, C	45.2, C	45.2, C	45.3, C	46.2, C	47.6, C	45.2, C
2	41.3, CH	41.2, CH	41.0, CH	41.0, CH	41.0, CH	49.8, CH	41.3, CH
3	39.8, CH_2_	39.5, CH_2_	39.5, CH_2_	39.6, CH_2_	46.4, CH_2_	67.5, CH	40.1, CH_2_
4	73.7, CH	73.6, CH	73.5, CH	73.7, CH	216.9, C	35.4, CH_2_	74.1, CH
5	40.0, CH	39.8, CH	39.8, CH	39.8, CH	46.1, CH	29.9, CH	40.3, CH
6	49.7, C	49.8, C	50.2, C	50.0, C	51.1, C	50.4, C	50.1, C
7	28.0, CH_2_^a^	29.3, CH_2_^a^	29.0, CH_2_	29.6, CH_2_	29.1, CH_2_	29.4, CH_2_	29.3, CH_2_
8	32.1, CH_2_	26.8, CH_2_	29.4, CH_2_	24.4, CH_2_	28.9, CH_2_	29.6, CH_2_	28.0, CH_2_^a^
9	155.9, C	112.4, C	146.7, C	153.4, C	145.9, C	146.2, C	134.7, C
10	131.0, C	153.1, C	150.5, C	149.2, C	149.9, C	149.8, C	143.7, C
11	171.3, C	174.4, C	200.0, C	200.1, C	198.0, C	199.0, C	67.8, CH
12	78.4, CH_2_	47.3, CH_2_	59.8, CH_2_	58.7, CH_2_	60.0, CH_2_	59.8, CH_2_	45.8, CH_2_
13	46.3, C	44.9, C	39.4, C	38.9, C	39.9, C	40.6, C	45.4, C
14	50.5, CH	51.3, CH	51.5, CH	51.2, CH	53.3, CH	51.9, CH	52.9, CH
15	28.2, CH_2_^a^	29.2, CH_2_^a^	28.5, CH_2_	28.4, CH_2_	29.7, CH_2_	21.9, CH_2_	28.0, CH_2_^a^
16	24.9, CH_3_	24.9, CH_3_	24.9, CH_3_	24.9, CH_3_	24.8, CH_3_	26.1, CH_3_	24.9, CH_3_
17	22.9, CH_3_	23.0, CH_3_	22.7, CH_3_	22.8, CH_3_	22.4, CH_3_	22.0, CH_3_	23.3, CH_3_
18	21.2, CH_3_	21.0, CH_3_	21.2, CH_3_	21.2, CH_3_	17.0, CH_3_	20.8, CH_3_	21.4, CH_3_
19	20.7, CH_3_	22.1, CH_3_	21.8, CH_3_^a^	22.0, CH_3_	21.7, CH_3_	21.3, CH_3_	23.3, CH_3_
20	22.6, CH_3_	19.5, CH_3_	21.7, CH_3_^a^	66.8, CH_2_	22.4, CH_3_	22.5, CH_3_	19.8, CH_3_

^a^Signals are exchangeable.

**Figure 3:**
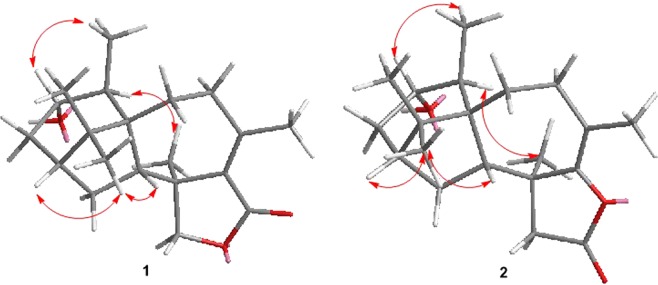
Selected NOESY correlations of 1 and 2.

The NOESY correlations of H_3_-16 with H-2 and H-14 indicated a *cis*-relationship of them (Fig. 3). Hence, ring D was oriented on the opposite face of ring C relative to these protons. The NOESY correlation of H-4/H_3_-18 indicated an *anti*-relationship of 4-OH and H_3_-18. The vicinity of H-5 and C-19 was deduced by the correlation of H-5/H_3_-19 in the NOESY spectrum.

**Figure 4:**
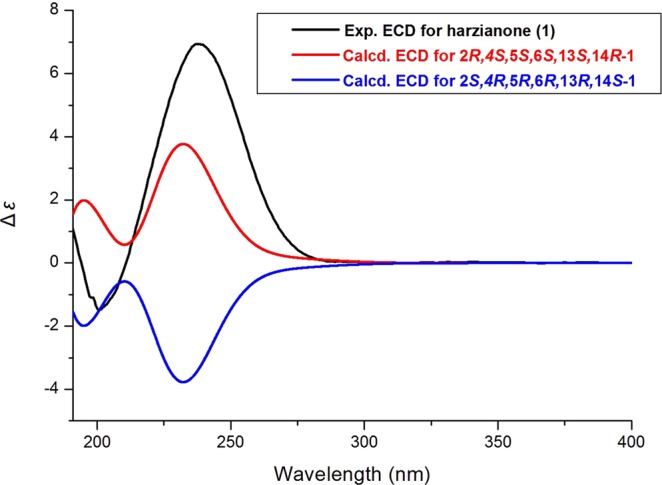
Experimental ECD spectra of 1 and calculated ECD spectra for (2*S*, 4*R*, 5*R*, 6*R*, 13*R*, 14*S*)-1 and (2*R*, 4*S*, 5*S*, 6*S*, 13*S*, 14*R*)-1.

The absolute configuration of **1** was established by comparison of its calculated and observed ECD and optical rotations (OR) data (see Supplementary Information). The predicted ECD for (2R, 4S, 5S, 6S, 13S, 14R)-**1** was in agreement with the experimental result of **1** (*λ*_max_ (Δ*ε*) 200 (−1.55), 239 (+7.03) nm) (Fig. 4). The computed ORs in the gas phase were –38.8 for (2*S*, 4*R*, 5*R*, 6*R*, 13*R*, 14*S*)-**1**, and +38.8 for (2*R*, 4*S*, 5*S*, 6*S*, 13*S*, 14*R*)-**1**, respectively, and the experimental value was +34.0. Based on both of ECD and OR calculations, the absolute configuration of **1** was assigned as 2*R*, 4*S*, 5*S*, 6*S*, 13*S*, 14*R*.

Harziane diterpenes are a unique class of terpenes, and only 16 such skeletons have been reported^9,14–20^. The cyclization mechanism of these unique diterpenes was illuminated by studies of selectively ^13^C- and ^2^H-labeled synthetic mevalonolactone isotopologues^21^. Distinguishing **1** from classic harziane diterpenes such as harzianone and harziandione was the D ring, which is a product of a Baeyer-Villiger monooxygenase catalyzed oxidation of a 6/5/7/4 fused tetra-cyclic skeleton; only two such harziane diterpene lactones have been discovered^17,22^. Moreover, only one report has appeared on the absolute configuration of such a harziane diterpene lactone (harzianelactone) by comparison its optical rotation data with that of the classic harziane diterpene, harzianone^17^. This report is the first to determine the absolute configuration of a harziane diterpene lactone by comparison of calculated and observed ECD spectra.

Harzianelactone B (**2**) was also isolated as a colorless oil and was assigned the same molecular formula C_20_H_30_O_3_ as **1** by HRESIMS results. Extensive analysis of the 1D and 2D NMR data indicated that **2** was also a harziane diterpene lactone, possessing a 6/5/7/5-fused tetra-cyclic ring scaffold like **1**. Compared to **1**, the disappearance of the oxymethylene signals in the ^1^H (*δ*_H_ 3.80, 3.73) and ^13^C NMR (*δ*_C_ 78.4) spectra, and its replacement with ketomethylene signals at *δ*_H_ 2.46, 2.33, and *δ*_C_ 47.3, and the significant downfield shift of C-10, as well as the different UV absorption (**2**: *λ*_max_ = 204 nm; **1**: *λ*_max_ = 237 nm), in combination with biogenetic considerations, suggested that C-10 is connected to the O-atom of the ester carbonyl. Detailed analysis of the HMBC correlations from H-8 and H_3_-20 to C-10, from H-12 to C-11, from H-14 to C-12, and from H-15 to C-13 (Fig. 2) also confirmed the structure. The relative configuration of **2** was deduced to be identical to that of **1** from the assignments of the cross-peaks in its NOESY spectrum (Fig. 3). The computed OR was +21.7 for (2*R*, 4*S*, 5*S*, 6*S*, 13*S*, 14*R*)-**2**, and the experimental value was +18.9 (see Supplementary Information). Therefore, the absolute configuration of **2** was determined as 2*R*, 4*S*, 5*S*, 6*S*, 13*S*, 14*R*. This is the first report of the absolute configuration of harziane diterpene lactones with the acyloxy group connected to C-10, such as **2**.

**Figure 5:**
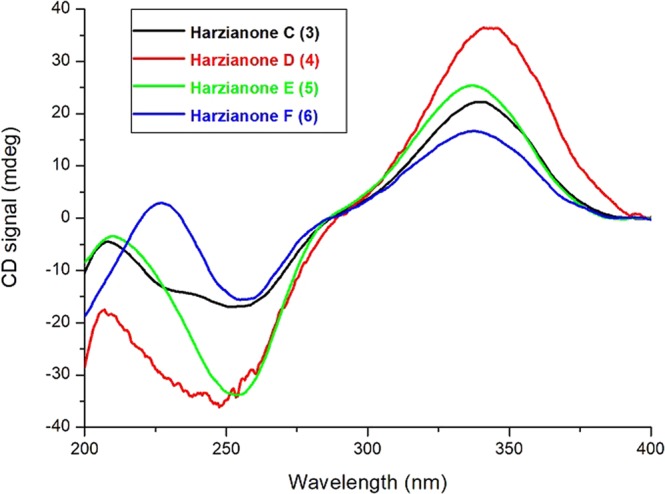
Experimental ECD spectra of 3–6.

Harzianone A (**3**) was obtained as a colorless oil. Its molecular formula of C_20_H_30_O_2_ (six degrees of unsaturation) was determined by HRESIMS and NMR data (Tables 1, 2). The UV absorption and the IR spectrum as well as the NMR data showed that **3** was also a harziane diterpene. Inspection of its NMR data revealed that **3** was similar to harzianone, which was isolated from an alga-endophytic isolate of *T. longibrachiatum*^9^. The difference between these two compounds was on the A ring, especially the chemical shifts of C-2 to C-4. In the ^1^H and ^13^C NMR spectra, the replacement of a methylene in harzianone with an oxymethine (*δ*_H_ 3.82; *δ*_C_ 73.5), and the downfield shift of C-2 to C-4 indicated that there was a hydroxy group anchored at one of these three carbons. The COSY correlations of H-2/H-3/H-4/H-5/H-18 and the HMBC correlations from H-4 to C-2, C-3, C-5, C-6 and C-18 suggested the hydroxy group was attached to C-4. Therefore, **3** has a 6/5/7/4-fused tetra-cyclic ring scaffold different from **1** and **2**. Analysis of the NOESY spectrum allowed the relative configuration of **3** to be the same as those of **1** and **2**. The positive first Cotton effect at 340 nm (Δ*ε* + 4.09) and the negative second one at 251 nm (Δ*ε* −3.11) (Fig. 5) in the ECD spectrum was consistent with that of harzianone^9^, thus indicating a 2 *R*,4 *S*,5 *S*,6 *S*,13 *S*,14 *S* absolute configuration of **3**.

As illustrated above, **1** and **2** might formed from **3** through a Baeyer-Villiger monooxygenase catalyzed oxidation. The different oxygenation position would from **1** and **2**, respectively. On the basis of biogenetic considerations, **1**−**3** should have the same configurations, which were in accordance with the description we discussed above.

Harzianone B (**4**) was afforded as a colorless oil, and had a molecular formula of C_20_H_30_O_3_ evidenced from its HRESIMS spectrum. Analysis of the NMR data demonstrated that the structure of **4** resemble that of **3**. The ^1^H and ^13^C NMR spectra of **4** displayed signals of four methyl groups, while those of **3** revealed five ones. Compared to **3**, the C-20 methyl group was replaced by an oxymethylene in **4**, which was defined by the HMBC correlations from H-20 to C-8, C-9, and C-10. As expected, subsequent analyses of the coupling constants, NOESY correlations, and experimental ECD data (Fig. 5) indicated that **4** has the same absolute configuration (2*R*, 4*S*, 5*S*, 6*S*, 13*S*, 14*S*) as that of **3**.

**Figure 6:**
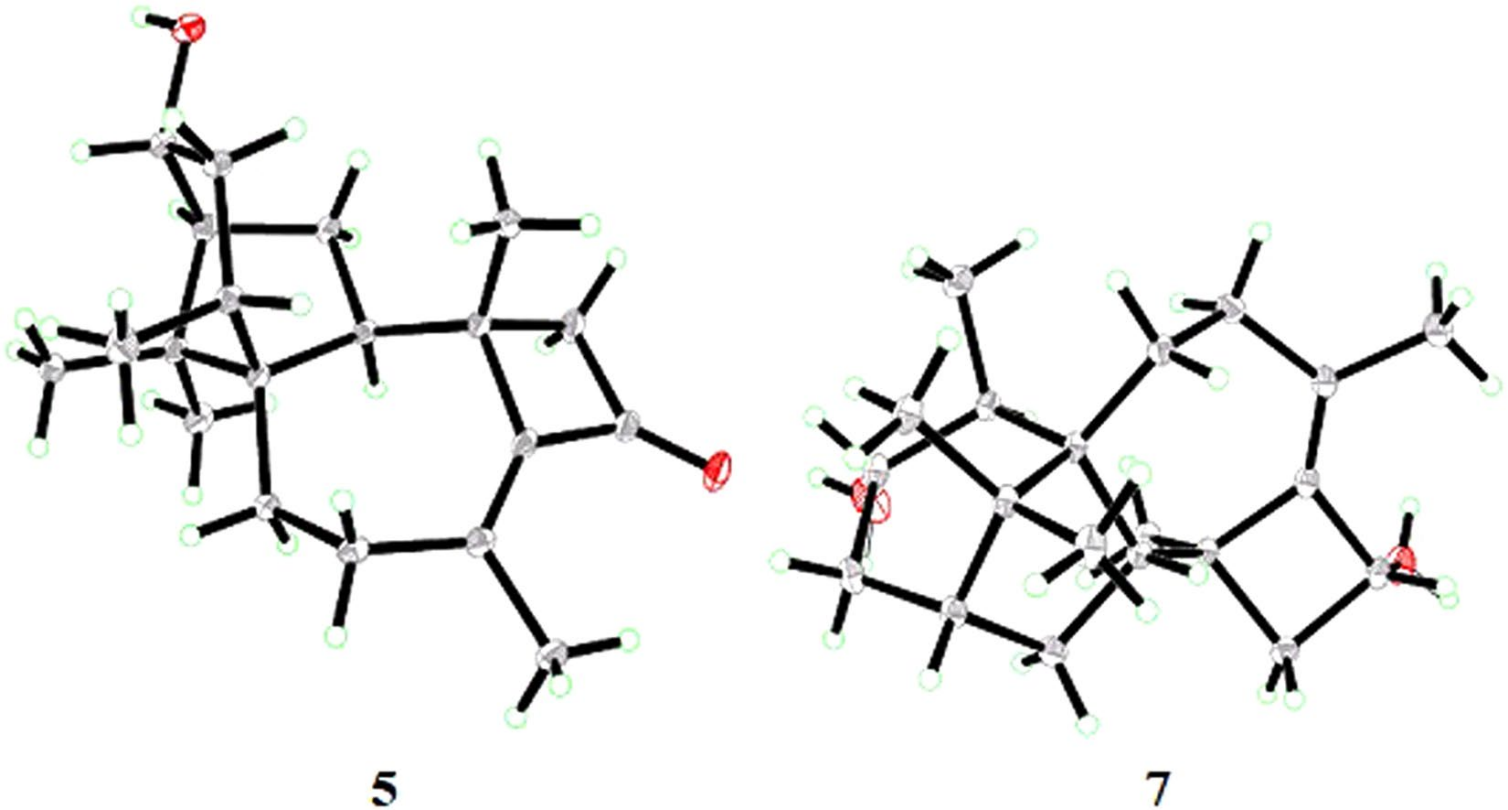
X-ray ORTEP diagrams of compounds 5 and 7.

Harzianone C (**5**) was isolated as colorless crystal needles. The molecular formula, C_20_H_30_O_2_, was assigned to be the same as that of **3** by its HRESIMS. The ^1^H and ^13^C NMR spectra of **5** showed similar characteristic signals to **3** (Tables 1, 2), except for the chemical shifts around the oxymethine group. In the ^1^H NMR spectrum, the oxymethine proton appeared as a multiplet, which was different from the doublets for **1**−**4**, and indicated the position of the hydroxy group was changed in **5**. In the HMBC spectrum, the correlations of H-4 with C-6, of H-15 with C-3, and of H_3_-18 with C-4 indicated the hydroxyl group was attached to C-3. The NOESY correlations from H-3 to H_3_-18 suggested that 3-OH and H_3_-18 are on the opposite sites of ring A. The relative configurations of the other chiral centers were confirmed to be the same as those of **3**. The ECD spectrum of **5** showed the same pattern as those of **3** and **4** (Fig. 5), suggesting that their chirality centers have the same absolute configurations. In addition, an X-ray crystallographic study (Fig. 6) was performed to confirm unambiguously the structure and determined the absolute configuration of **5** as 2*S*, 4*S*, 5*S*, 6*S*, 11*R*, 13*S*, 14*S*.

Harzianone D (**6**) was obtained as a colorless oil. The NMR spectral features suggested that **6** was closely related to **3**. The additional carbonyl group (*δ*_C_ 216.9) and the disappearance of the oxymethine group (*δ*_C_ 73.5; *δ*_H_ 3.82 in **3**) in **6** indicated that the hydroxy group at C-4 in **3** was replaced by a carbonyl group in **6**, which was confirmed by the HMBC correlations from H-2, H-3, H-5, and H-18 to C-4. The relative configuration was determined as the same as **1**−**5** through NOESY spectrum. Similar cotton effects observed for **6** (Δ*ε* 223 + 4.16, Δ*ε* 280 −1.36) to **3** and **4** in their ECD spectra (Fig. 5) indicated that they shared the same absolute configurations of (2*R*, 5*S*, 6*S*, 13*S*, 14*S*).

Harziane (**7**) was obtained as colorless crystals. Its molecular formula, C_20_H_32_O_2_, was deduced from its HRESIMS data with five indices of hydrogen deficiency, one fewer than those of **1**−**5**. The ^1^H and ^13^C NMR data (Tables 1, 2) of **7** and **3** were very similar with each other except for those in the vicinity of C-11. In the ^13^C NMR spectra, the signals for *α,β*-unsaturated ketone (*δ*_C_ 200.0) in **3** was disappeared and one more oxymethine (*δ*_C_ 67.8) was emerged in **7**. These evidences as well as the unsaturation degrees of these two compounds indicated that the ketone carbonyl group at C-11 in **3** was replaced by an oxymethine group in **7**, which was confirmed by the HMBC correlations from H-11 to C-9, C-10, and C-13. The relative configuration of all chiral centers but C-11 was determined by the NOESY spectrum of **7** like **1**−**6**, and the correlation of H-11 with H_3_-18 indicated they were *cis*-oriented. To clarify its absolute stereochemistry, **7** was recrystallized in a dichloromethane/methanol (20:1) mixture to yield crystals. The low-temperature X-ray diffraction (CuK*α*) of the single crystals (Fig. 6) revealed that **7** had a (2*R*, 4*S*, 5*S*, 6*S*, 11*R*, 13*S*, 14*S*)-configuration.

Harziane diterpenes have rarely been reported to have significant bioactivities. In the present study, compounds **1**−**5** and **7** were evaluated for their phytotoxic and antibacterial activities. All the tested compounds showed obvious phytotoxicity against the seedling growth of amaranth and lettuce at a concentration of 200 ppm (Table 3). Compounds **1**, **3**, **4**, and **5** were more effective as they could completely inhibit seed germination against amaranth at 200 μg/mL, and this strong phytotoxicity was still evident at lower concentrations (50 μg/mL), compared to the positive control glyphosate. No compound was found to inhibit the root growth of lettuce at 200 ppm. It seemed that the isolated compounds caused weaker inhibition to lettuce than to amaranth, and have stronger toxicity on the growth of root growth than hypocotyl. Although there are three reports on the phytotoxicity of crude extracts of *Trichoderma* spp.^4,23,24^, no one had studied the phytotoxicity of compounds from *Trichoderma* spp. Thus, this is the first report of the phytotoxic compounds from *Trichoderma* spp., and the phytotoxicity of harziane diterpenes is also reported for the first time. None of the isolated compounds exhibited antibacterial activities.

**Table 3:** Phytotoxity of compounds **1**–**5**, and **7** against seedling growth.

Compounds	Root length (mm)		Hypocotyl length (mm)		
	Amaranth (200 ppm)	Amaranth (50 ppm)	Amaranth (200 ppm)	Amaranth (50 ppm)	Lettuce (200 ppm)
**1**	0	5.7 ± 0.2	0	6.5 ± 0.1	7.4 ± 0.4
**2**	12.8 ± 1.6	—	6.7 ± 0.4	—	6.4 ± 0.8
**3**	0	7.9 ± 0.5	0	6.3 ± 0.1	6.2 ± 0.5
**4**	0	4.9 ± 1.3	0	3.8 ± 0.8	6.5 ± 0.6
**5**	0	9.2 ± 1.0	0	6.5 ± 0.5	7.6 ± 0.2
**7**	5.6 ± 1.3	10.2 ± 0.3	3.7 ± 0.8	7.1 ± 0.7	6.1 ± 0.4
glyphosate	0	0	1.4 ± 0.3	0	3.8 ± 0.1
H_2_O	15.0 ± 1.0	15.0 ± 1.0	8.5 ± 0.4	8.5 ± 0.4	9.0 ± 0.5

“—” means no phytotoxicity; all of the compounds has no phytotoxicity on root elongation of lettuce.

## Conclusions

In summary, the present chemical investigation on the soft coral-derived *T. harzianum* XS-20090075 resulted in the discovery of a series of harziane diterpenes (**1**–**7**). Compounds **1** and **2** represent a unique type of harziane diterpene lactone derived from harziane diterpenes though Baeyer-Villiger monooxygenase catalyzed oxidations. Harziane diterpenes have rarely been studied, and only 18 such compounds have been reported, including two harziane diterpene lactones. In this study, the structures of harziane diterpenes were determined by NMR spectroscopic data, ECD and OR calculations, together with X-ray diffraction. The phytotoxicity of compounds from *Trichoderma* sp. was evaluated for the first time, and the isolated compounds exhibited potent phytotoxicity towards amaranth and lettuce.

## Methods

### General experimental procedures

Optical rotations were measured using a P-1020 polarimeter (JASCO). UV spectrua were obtained with a DU 640 spectrophotometer (Beckman). ECD spectra were acquired on a JASCO J-815-150S CD spectrometer. IR spectra were obtained via a Nicolet-Nexus-470 spectrometer. NMR spectra were recorded on an Agilent DD2 NMR spectrometer (500 MHz). ESIMS and HRESIMS spectra were obtained by a Q-TOF (Micromass) and a LTQ Orbitrap XL (Thermo Scientific) spectrometer, respectively. Single-crystal analysis were performed on a Gemini A Ultra system using Cu K*α* radiation (Aglient Technologies). A 1525 separation module (Waters) equipped with a C_18_ (Kromasil, 5 *μ*m, 10 × 250 mm) column was used for semi-preparative HPLC. ODS (Unicorn; 45–60 μm), Sephadex LH-20 (Amersham Biosciences), and silica gel (200–300 mesh; Qing Dao Hai Yang Chemical Group Co.) were applied for column chromatography. TLC (G60, F-254; Yan Tai Zi Fu Chemical Group Co.) was used in the compounds detection.

### Fungal materia

The fungal strain (XS-20090075) was isolated from the inner part of an unidentified soft coral, and was identified as *T. harzianum* by morphological characteristics and ITS sequence. A voucher specimen was deposited at School of Medicine and Pharmacy, Ocean University of China, PR China (KU866299).

### Extraction and isolation

The fungus XS-20090075 was fermented at room temperature for four weeks in 100 conical flask (1 L) containing 80 g rice and 120 mL H_2_O with 3% salinity. The culture medium was extracted by EtOAc and CH_2_Cl_2_−MeOH (v/v, 1:1) for three times, and the solution was concentrated under reduced pressure to afford a residue. The residue was mixed with 1000 mL of H2O, and extracted with ethyl acetate to yield the crude extract (18.5 g). The extract was fractioned by silica gel column chromatography (CC) eluted with gradient EtOAc in petroleum ether (0%–100%), and then with MeOH/EtOAc (10%–50%) to yield six fractions (Fr. 1−Fr. 6). Fr. 1 was first repeatedly chromatographed on silica gel column by EtOAc/petroleum ether (10%), and then separated by ODS eluted with MeOH−H_2_O (30−80%) to afford Fr. 1-1−Fr. 1–5. Fr. 1–3 was further purified over semipreparative RP-HPLC (MeOH/H_2_O, 80/100) to yiled **2** (24.0 mg), **3** (94.3 mg), and **6** (4.2 mg). Fr. 2 was first separated by silica gel CC (EtOAc/petroleum ether = 20/80), and the eluent were combined, concentrated, and submitted to Sephadex LH-20 CC (CH_2_Cl_2_/MeOH, v/v, 1/1), followed by purification on HPLC with 55% MeOH−H_2_O to afford **1** (8.9 mg) and **5** (22.8 mg). Fr. 3 was chromatographed on silica gel CC (EtOAc/petroleum ether = 20%) and separated by ODS CC using 50% MeOH−H_2_O to obtain Fr. 3-1–Fr. 3-3. Fr.3-1 was futher purified on HPLC (65% MeOH−H_2_O) to give **4** (38.2 mg). Fr. 3-2 was purified by semipreparative RP-HPLC (MeOH/H_2_O, 80/20) to yield **7** (6.4 mg).

#### Harzianelactone A (1)

colorless oil; [*α*]^20^_D_ + 33.8 (*c* 0.42, MeOH); UV (MeOH) *λ*_max_ (log *ε*) 237 (3.81) nm; ECD (1.57 mM, MeOH) *λ*_max_ (Δ*ε*) 200 (−1.55), 239 (+7.03) nm; IR (KBr) *ν*_*max*_ 3425, 2933, 2362, 2340, 1738, 1653, 1029 cm^–1^; ^1^H and ^13^C NMR data, Tables 1, 2; HRESIMS *m/z* 319.2263 [M + H]^+^ (calcd for C_20_H_31_O_3_, 319.2268).

#### Harzianelactone B (2)

colorless oil; [*α*]^20^_D_ + 18.9 (*c* 0.42, MeOH); UV (MeOH) *λ*_max_ (log *ε*) 204 (3.62) nm; IR (KBr) *ν*_*max*_ 3398, 2931, 2362, 2340, 1779, 1703, 1029 cm^−1^; ^1^H and ^13^C NMR data, Tables 1, 2; ESIMS *m/z* 319.3 [M + H]^+^, 341.3 [M + Na]^+^, 637.4 [2 M + H]^+^, 659.5 [2 M + Na]^+^; HRESIMS *m/z* 319.2271 [M + H]^+^ (calcd for C_20_H_31_O_3_, 319.2268).

#### Harzianone A (3)

colorless oil; [*α*]^20^_D_ + 72.1 (*c* 0.42, MeOH); UV (MeOH) *λ*_max_ (log *ε*) 259 (3.98) nm; ECD (1.65 mM, MeOH) *λ*_max_ (Δ*ε*) 251 (−3.11), 340 (+4.09) nm; IR (KBr) *ν*_*max*_ 3400, 2932, 2361, 1735, 1669, 1441, 1260, 1150, 1027 cm^−1^; ^1^H and ^13^C NMR data, Tables 1, 2; HRESIMS *m/z* 303.2316 [M + H]^+^ (calcd for C_20_H_31_O_2_, 303.2319).

#### Harzianone B (4)

colorless oil; [*α*]^20^_D_ + 32.2 (*c* 0.41, MeOH); UV (MeOH) *λ*_max_ (log *ε*) 203 (3.49), 256 (3.64) nm; ECD (1.57 mM, MeOH) *λ*_max_ (Δ*ε*) 248 (−6.98), 345 (+7.02) nm; IR (KBr) *ν*_*max*_ 3425, 2935, 2363, 1722, 1689, 1029 cm^−1^; ^1^H and ^13^C NMR data, Tables 1, 2; HRESIMS *m/z* 319.2262 [M + H]^+^ (calcd for C_20_H_31_O_3_, 319.2268).

#### Harzianone C (5)

colorless crystals; mp 168−169 °C; [*α*]^20^_D_ + 14.7 (*c* 0.46, MeOH); UV (MeOH) *λ*_max_ (log *ε*) 256 (3.58) nm; ECD (1.65 mM, MeOH) *λ*_max_ (Δ*ε*) 254 (−6.21), 337 (+4.66) nm; IR (KBr) *ν*_*max*_ 3398, 2932, 2363, 1737, 1659, 1444, 1382, 1020 cm^−1^; ^1^H and ^13^C NMR data, Tables 1, 2; HRESIMS *m/z* 303.2318 [M + H]^+^ (calcd for C_20_H_31_O_2_, 303.2319).

#### Harzianone D (6)

colorless oil; [*α*]^20^_D_ + 52.6 (*c* 0.28, MeOH); UV (MeOH) *λ*_max_ (log *ε*) 255 (2.72) nm; ECD (1.67 mM, MeOH) *λ*_max_ (Δ*ε*) 255 (−2.83), 338 (+3.02) nm; IR (KBr) *ν*_*max*_ 2948, 2356, 1728, 1655, 1438, 1260, 1022 cm^−1^; ^1^H and ^13^C NMR data, Tables 1, 2; HRESIMS *m/z* 301.2161 [M + H]^+^ (calcd for C_20_H_29_O_2_, 301.2162).

#### Harziane (7)

colorless crystals; mp 214−215 °C; [*α*]^20^_D_ + 5.1 (*c* 0.48, MeOH); UV (MeOH) *λ*_max_ (log *ε*) 206 (3.58) nm; IR (KBr) *ν*_*max*_ 3404, 2929, 2362, 1653, 1382, 1033 cm^−1^; ^1^H and ^13^C NMR data, Tables 1, 2; HRESIMS *m/z* 287.2364 [M + H − H_2_O]^+^ (calcd for C_20_H_31_O, 287.2369).

### X-ray Crystallographic Analysis of 5 and 7

The Single-crystal X-ray diffraction data were recorded on a Xcalibur, Atlas, Gemini ultra diffractometer at 120 K. Crystallographic data for **5** (deposition NO. CCDC 1573734) and **7** (deposition NO. CCDC 1573693) have been deposited in the Cambridge Crystallographic Data Centre. Copies of the data can be obtained, free of charge, on application to the Director, CCDC, 12 Union Road, Cambridge CB21EZ, UK [fax: + 44(0)-1233-336033 or e-mail: deposit@ccdc.cam.ac.uk].

#### Crystal data for 5

C_20_H_30_O_2_, *M*_r_ = 302.44, monoclinic, *a* = 7.13670 (10) Å, *b* = 13.7978 (3) Å, *c* = 8.4352(2) Å, *α* = 90.00°, *β* = 94.516 (2)°, *γ* = 90.00°, *V* = 828.04 (3) Å^3^, space group *P*21, *Z* = 2, *D*_x_ = 1.213 mg/m^3^, *μ* = 0.586 mm^−1^, and *F* (000) = 332. Crystal size: 0.42 × 0.25 × 0.13 mm^3^. Reflections collected/unique: 8011/2950 [*R*(int) = 0.0230]. The final indices were *R*_1_ = 0.0295, *wR*_2_ = 0.0733 (*I* > 2*σ*(*I*)). Flack parameter = 0.13 (19).

#### Crystal data for 7

C_20_H_32_O_2_, *M*_r_ = 304.46, monoclinic, *a* = 18.9468 (16) Å, *b* = 8.3433 (2) Å, *c* = 13.245 (4) Å, *α* = 90.00°, *β* = 124.739 (8)°, *γ* = 90.00°, *V* = 1720.6 (5) Å^3^, space group *C*2, *Z* = 4, *D*_x_ = 1.175 mg/m^3^, *μ* = 0.564 mm^−1^, and *F* (000) = 672. Crystal size: 0.21 × 0.20 × 0.19 mm^3^. Reflections collected/unique: 9303/3064 [*R*(int) = 0.0261]. The final indices were *R*_1_ = 0.0307, *wR*_2_ = 0.0748 (*I* > 2*σ*(*I*)). Flack parameter = 0.13 (19).

### Phytotoxicity bioassays

Phytotoxicity against seeding growth of amaranth (*Amaranthus retroflexus* L.) and lettuce (*Lactuca sativa*) was assayed by the method reported previously^25^. Glyphosate was used as the positive control.

### Antibacterial assays

The antibacterial activity was evaluated by the conventional broth dilution assay^26^. Five pathogenic bacterial strains, including Gram-positive *Kocuria rhizophila* (ATCC 9341), *Staphyloccocus aureus* (ATCC 27154), and Gram-negative *Escherichia coli* (ATCC 25922), *Ralstonia solanacearum*, *Vibrio anguillarum* (ATCC 19019), and *V. Parahemolyticus* (ATCC 17802) were used, and ciprofloxacin and streptomycin sulfate were used as positive controls.

## Supplementary information

Supplementary Information
