# Supplementary material for: Potent Phytotoxic Harziane Diterpenes from a Soft Coral-Derived Strain of the Fungus Trichoderma harzianum XS-20090075
Source: Sci Rep. 2019 Sep 16;9:13345. doi: 10.1038/s41598-019-49778-7 (PMC6746854; doi:10.1038/s41598-019-49778-7)
Supplement: Supplementary file 1 — Supplementary Information [file 41598_2019_49778_MOESM1_ESM.docx]

***Supplementary Information***

**Potent Phytotoxic Harziane Diterpenes from a Soft Coral-Derived Strain of the Fungus *Trichoderma harzianum* XS-20090075**

Dong-Lin Zhao,^1,2^ Lu-Jia Yang,^1^ Ting Shi,^1^ Chao-Yi Wang,^1^ Chang-Lun Shao,^1,3,*^ and Chang-Yun Wang^1,3,4,*^

^1^ Key Laboratory of Marine Drugs, The Ministry of Education of China, School of Medicine and Pharmacy, Ocean University of China, Qingdao 266003, People’s Republic of China. ^2^ Marine Agricultural Research Center, Tobacco Research Institute of Chinese Academy of Agricultural Sciences, Qingdao 266101, People’s Republic of China. ^3^ Laboratory for Marine Drugs and Bioproducts, Qingdao National Laboratory for Marine Science and Technology, Qingdao 266237, People’s Republic of China. ^4^ Institute of Evolution & Marine Biodiversity, Ocean University of China, Qingdao 266003, People’s Republic of China

^*^ Correspondence and requests for materials should be addressed to C.-Y.W (changyun@ouc.edu.cn) or C.-L.S (shaochanglun@163.com)

**List of Supplementary Information**

**Figure S1.** ^1^H NMR (500 MHz, CDCl_3_) spectrum of compound **1**

**Figure S2.** ^13^C NMR (125 MHz, CDCl_3_) spectrum of compound **1**

**Figure S3.** HMQC (CDCl_3_) spectrum of compound **1**

**Figure S4.** COSY (CDCl_3_) spectrum of compound **1**

**Figure S5.** HMBC (CDCl_3_) spectrum of compound **1**

**Figure S6.** NOESY (CDCl3) spectrum of compound **1**

**Figure S7.** HRESIMS spectrum of compound **1**

**Figure S8.** ^1^H NMR (500 MHz, CDCl_3_) spectrum of compound **2**

**Figure S9.** ^13^C NMR (125 MHz, CDCl_3_) spectrum of compound **2**

**Figure S10.** HMQC (CDCl_3_) spectrum of compound **2**

**Figure S11.** COSY (CDCl_3_) spectrum of compound **2**

**Figure S12.** HMBC (CDCl_3_) spectrum of compound **2**

**Figure S13.** NOESY (CDCl3) spectrum of compound **2**

**Figure S14.** HRESIMS spectrum of compound **2**

**Figure S15.** ^1^H NMR (500 MHz, CDCl_3_) spectrum of compound **3**

**Figure S16.** ^13^C NMR (125 MHz, CDCl_3_) spectrum of compound **3**

**Figure S17.** HMQC (CDCl_3_) spectrum of compound **3**

**Figure S18.** COSY (CDCl_3_) spectrum of compound **3**

**Figure S19.** HMBC (CDCl_3_) spectrum of compound **3**

**Figure S20.** NOESY (CDCl3) spectrum of compound **3**

**Figure S21.** HRESIMS spectrum of compound **3**

**Figure S22.** ^1^H NMR (500 MHz, CDCl_3_) spectrum of compound **4**

**Figure S23.** ^13^C NMR (125 MHz, CDCl_3_) spectrum of compound **4**

**Figure S24.** HMQC (CDCl_3_) spectrum of compound **4**

**Figure S25.** COSY (CDCl_3_) spectrum of compound **4**

**Figure S26.** HMBC (CDCl_3_) spectrum of compound **4**

**Figure S27.** NOESY (CDCl3) spectrum of compound **4**

**Figure S28.** HRESIMS spectrum of compound **4**

**Figure S29.** ^1^H NMR (500 MHz, CDCl_3_) spectrum of compound **5**

**Figure S30.** ^13^C NMR (125 MHz, CDCl_3_) spectrum of compound **5**

**Figure S31.** HMQC (CDCl_3_) spectrum of compound **5**

**Figure S32.** COSY (CDCl_3_) spectrum of compound **5**

**Figure S33.** HMBC (CDCl_3_) spectrum of compound **5**

**Figure S34.** NOESY (CDCl3) spectrum of compound **5**

**Figure S35.** HRESIMS spectrum of compound **5**

**Figure S36.** ^1^H NMR (500 MHz, CDCl_3_) spectrum of compound **6**

**Figure S37.** ^13^C NMR (125 MHz, CDCl_3_) spectrum of compound **6**

**Figure S38.** HMQC (CDCl_3_) spectrum of compound **6**

**Figure S39.** COSY (CDCl_3_) spectrum of compound **6**

**Figure S40.** HMBC (CDCl_3_) spectrum of compound **6**

**Figure S41.** NOESY (CDCl3) spectrum of compound **6**

**Figure S42.** HRESIMS spectrum of compound **6**

**Figure S43.** ^1^H NMR (500 MHz, CDCl_3_) spectrum of compound **7**

**Figure S44.** ^13^C NMR (125 MHz, CDCl_3_) spectrum of compound **7**

**Figure S45.** HMQC (CDCl_3_) spectrum of compound **7**

**Figure S46.** COSY (CDCl_3_) spectrum of compound **7**

**Figure S47.** HMBC (CDCl_3_) spectrum of compound **7**

**Figure S48.** NOESY (CDCl3) spectrum of compound **7**

**Figure S49.** HRESIMS spectrum of compound **7**

**ECD and OR simulation of Compounds 1 and 2**


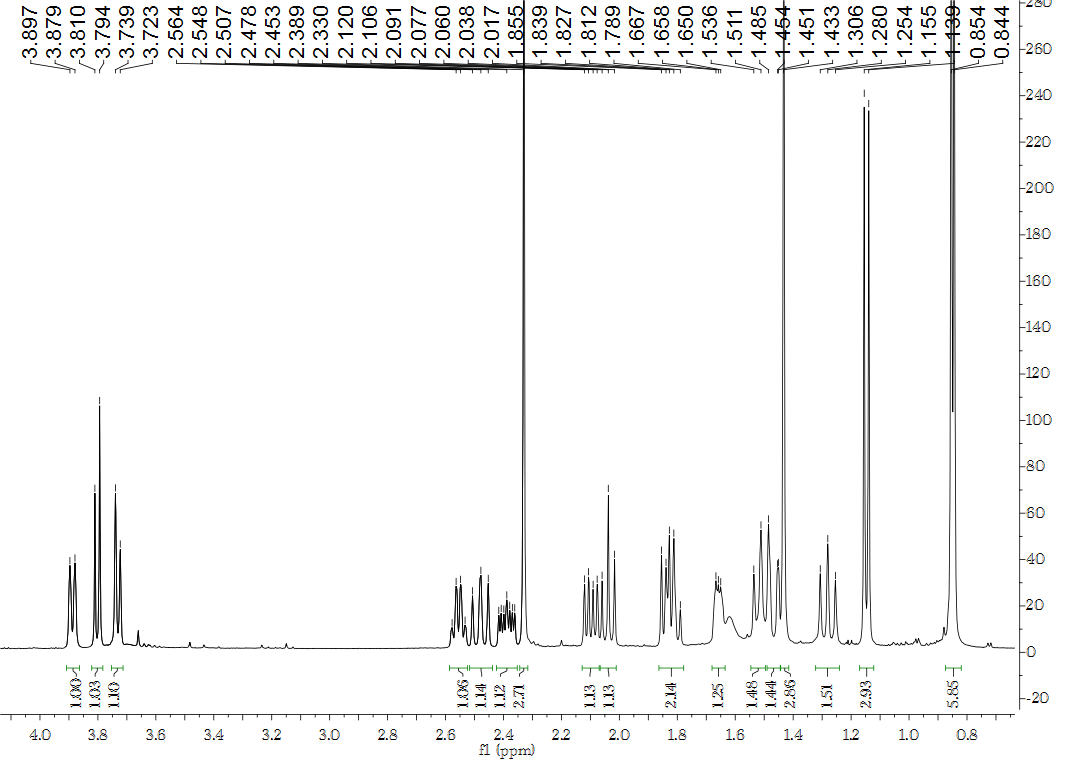


**Figure S1.** ^1^H NMR (500 MHz, CDCl_3_) spectrum of compound **1**


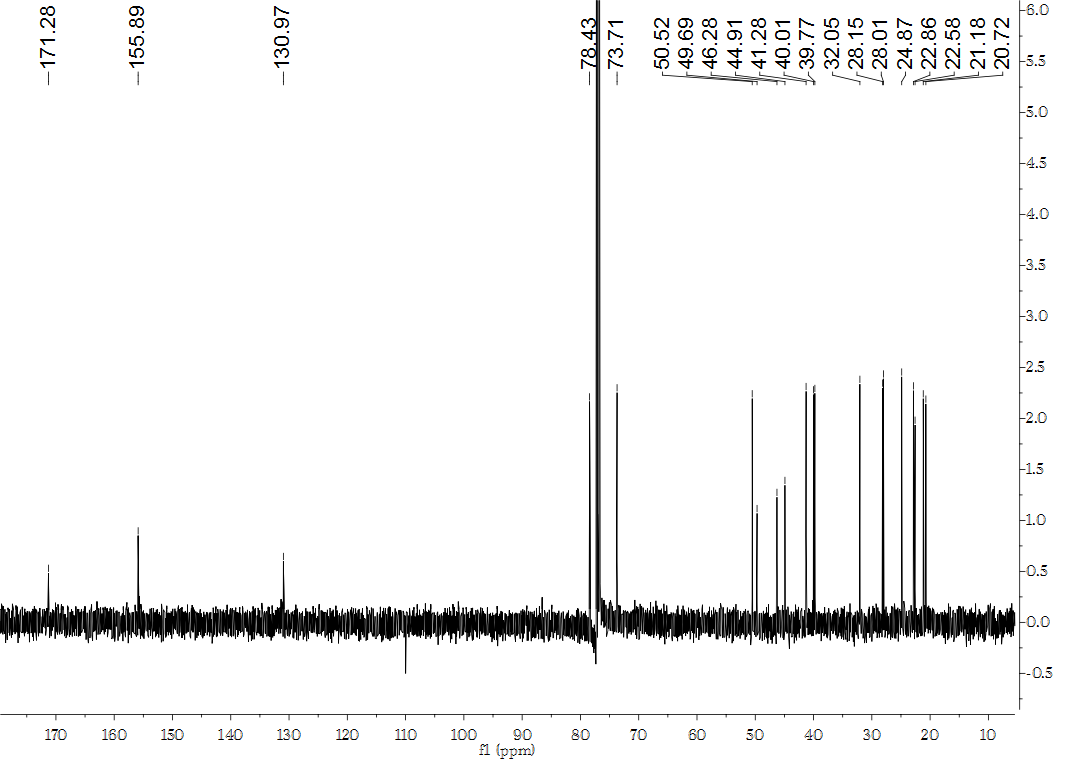
**Figure S2.** ^13^C NMR (125 MHz, CDCl_3_) spectrum of compound **1**


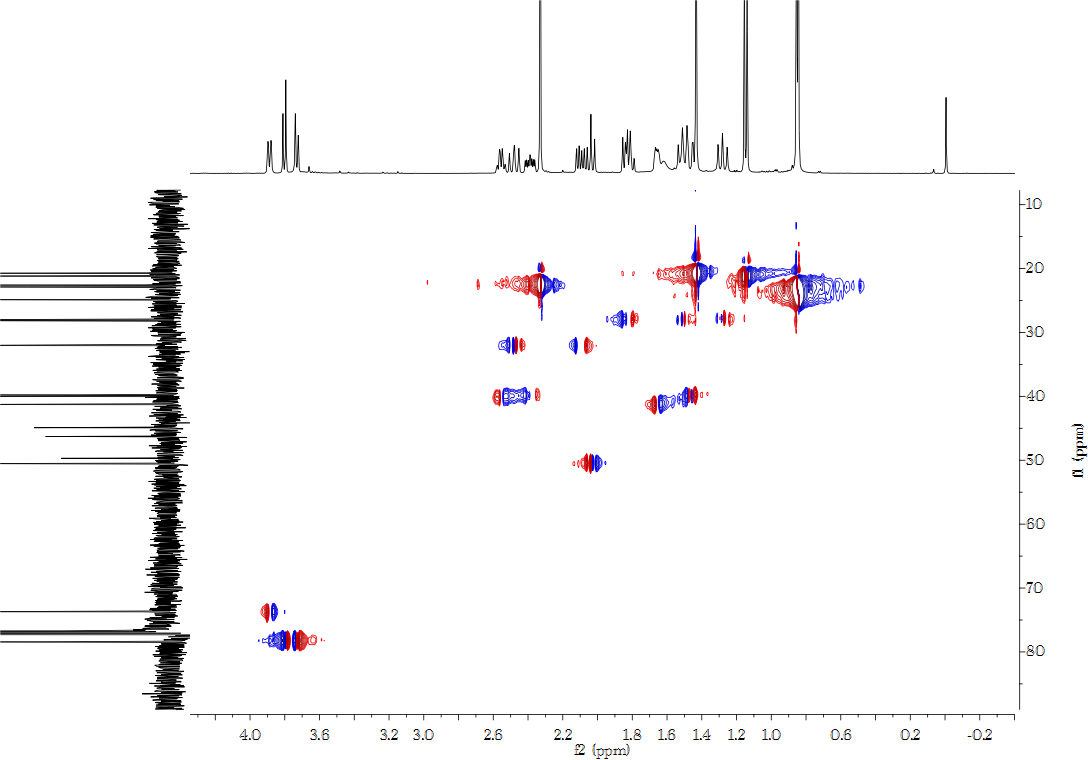


**Figure S3.** HMQC (CDCl_3_) spectrum of compound **1**


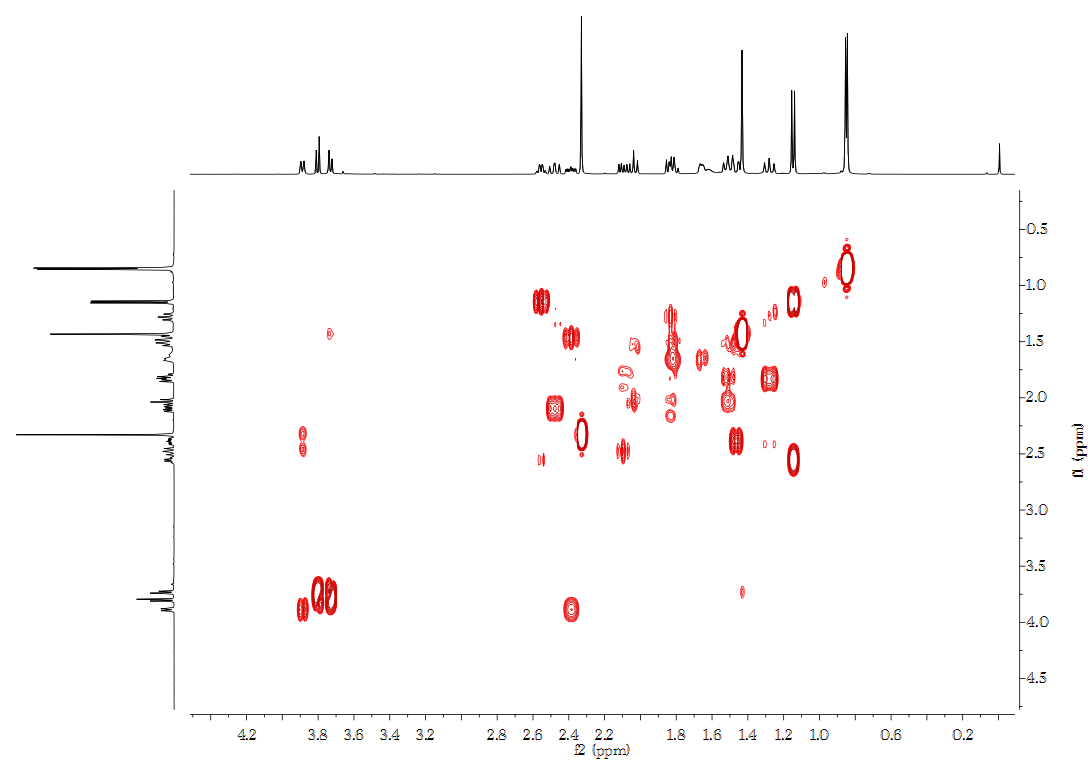
**Figure S4.** COSY (CDCl_3_) spectrum of compound **1**


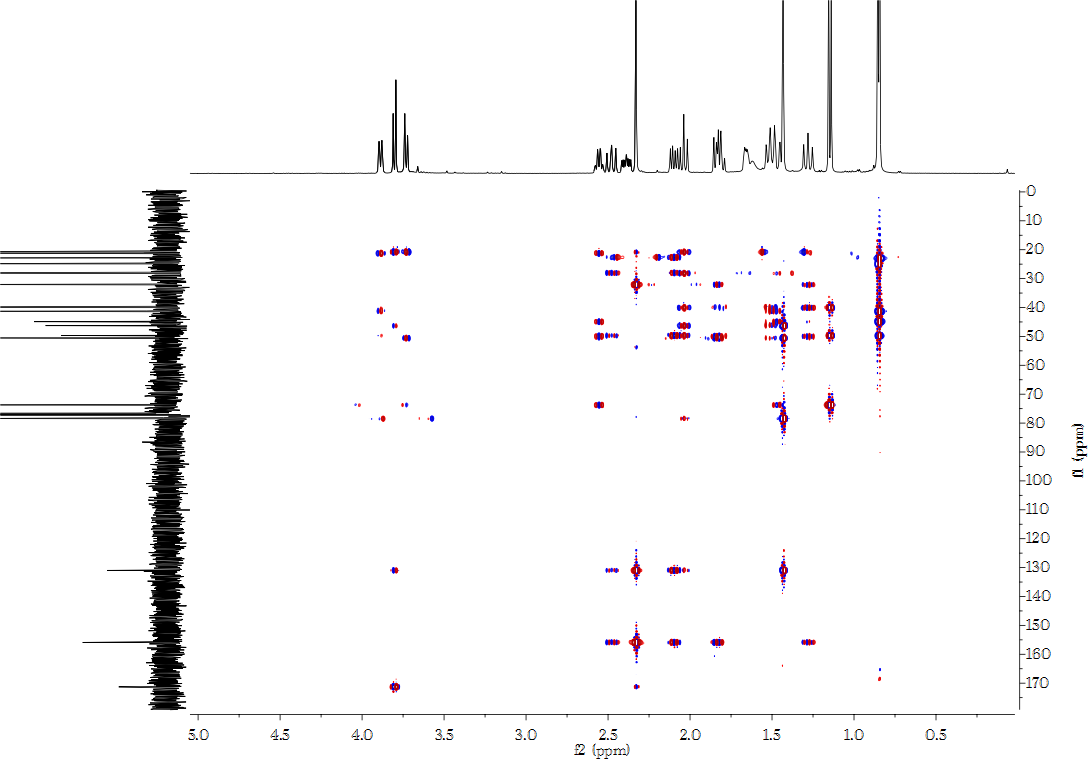


**Figure S5.** HMBC (CDCl_3_) spectrum of compound **1**


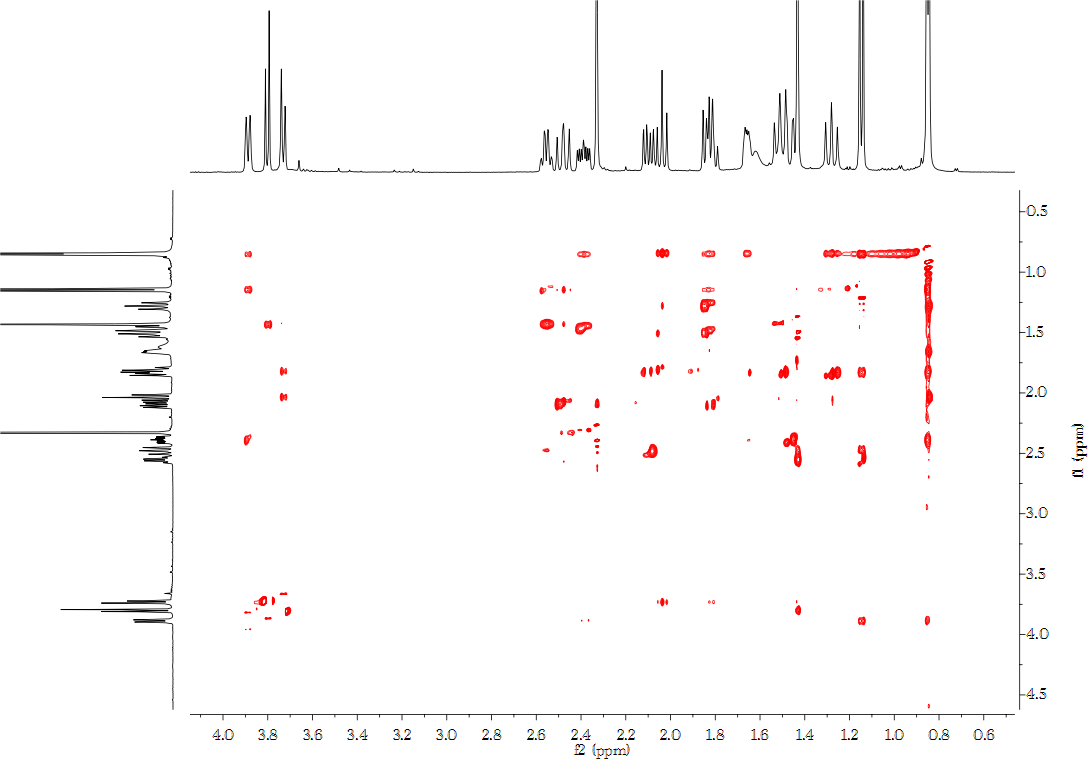


**Figure S6.** NOESY (CDCl_3_) spectrum of compound **1**


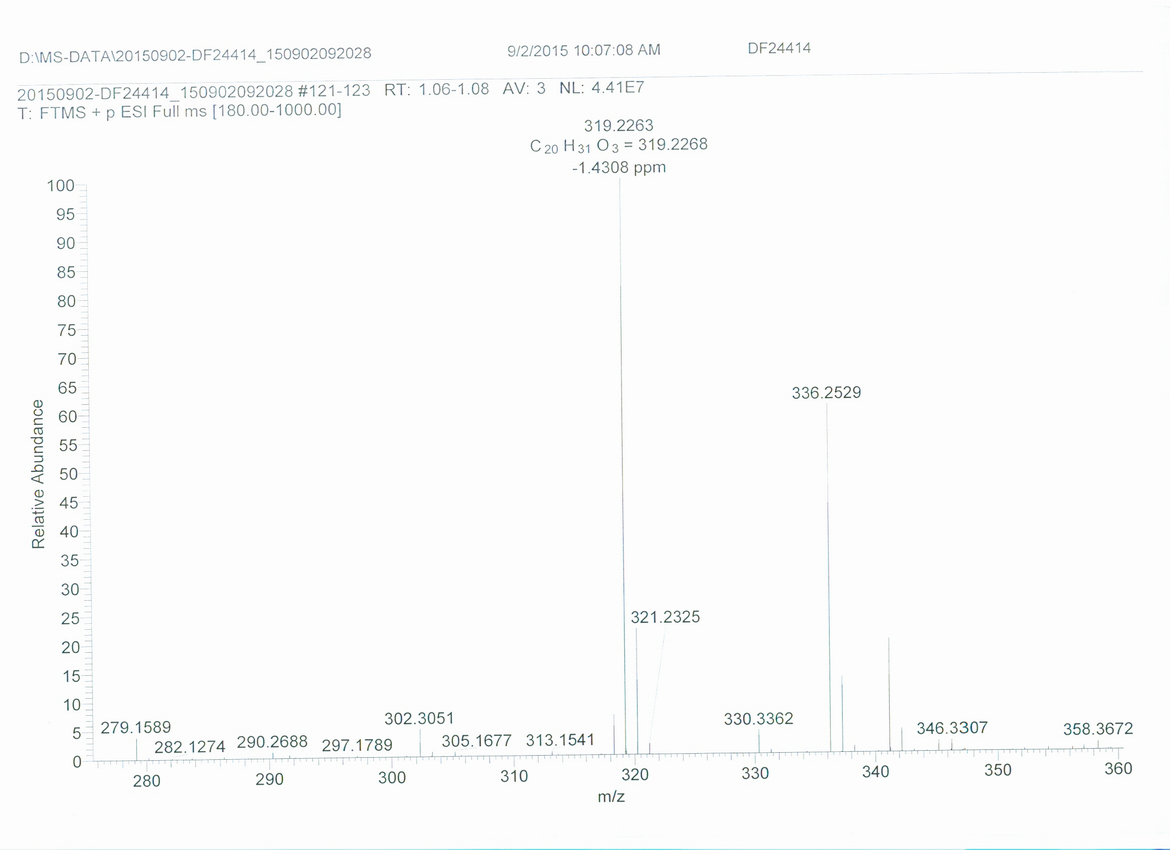


**Figure S7.** HRESIMS spectrum of compound **1**


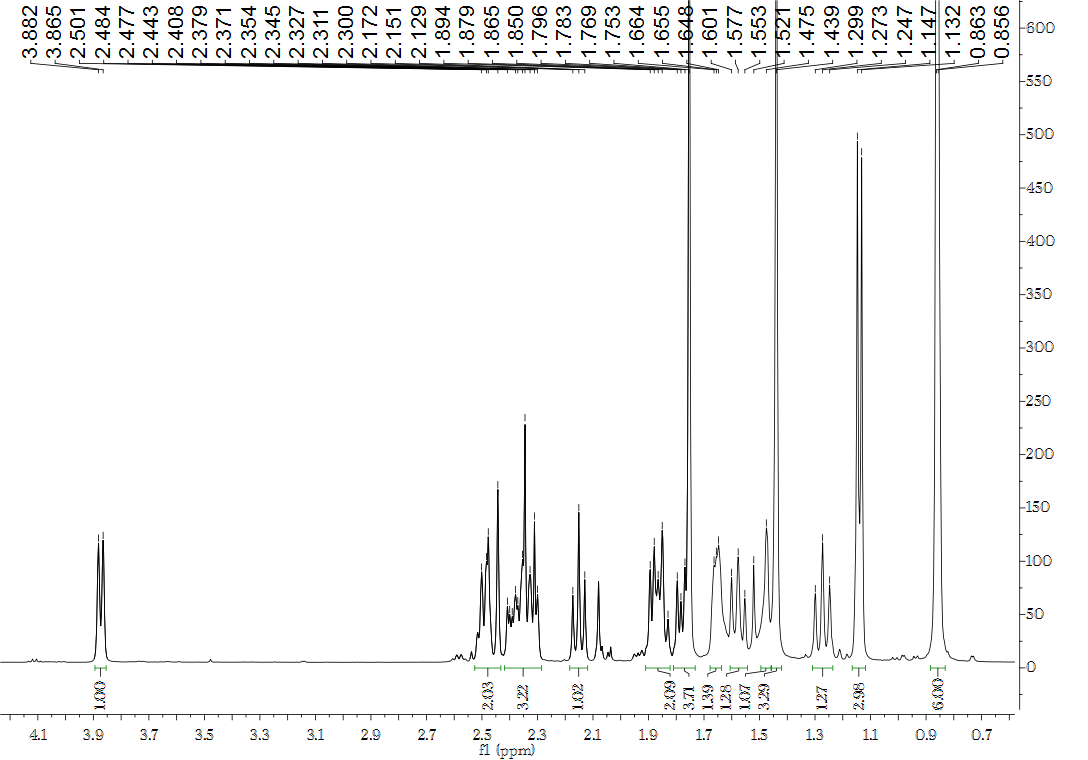


**Figure S8.** ^1^H NMR (500 MHz, CDCl_3_) spectrum of compound **2**


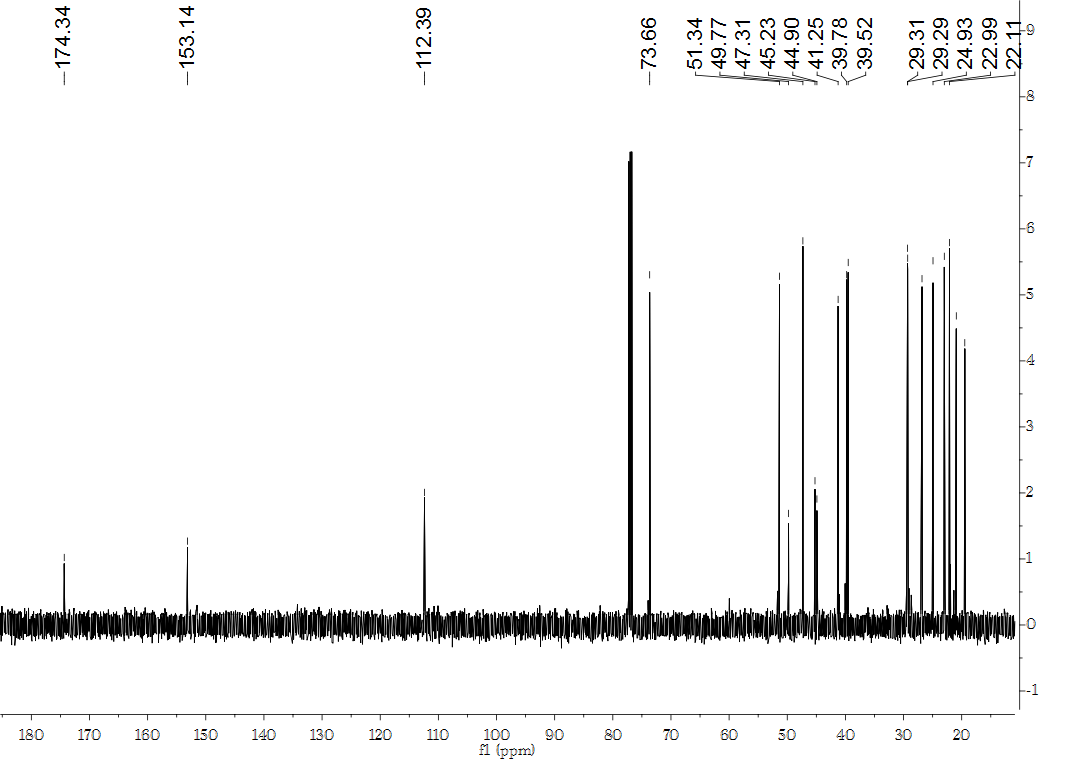


**Figure S9** ^13^C NMR (125 MHz, CDCl_3_) spectrum of compound **2**


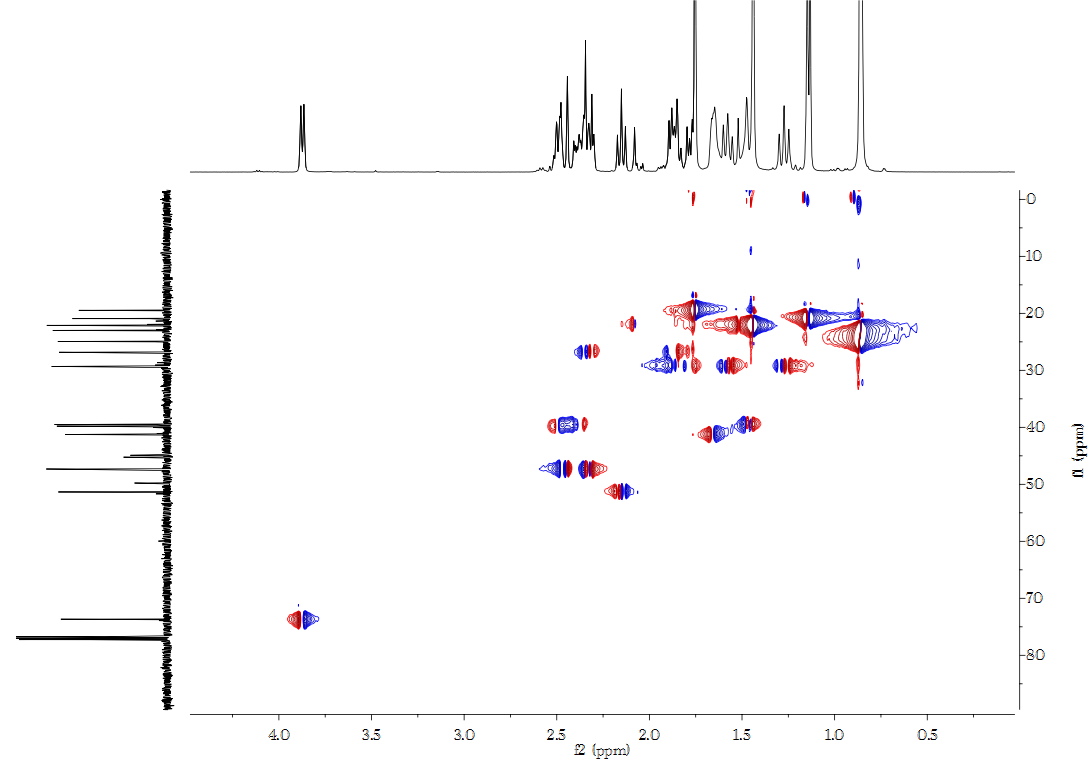


**Figure S10.** HMQC (CDCl_3_) spectrum of compound **2**


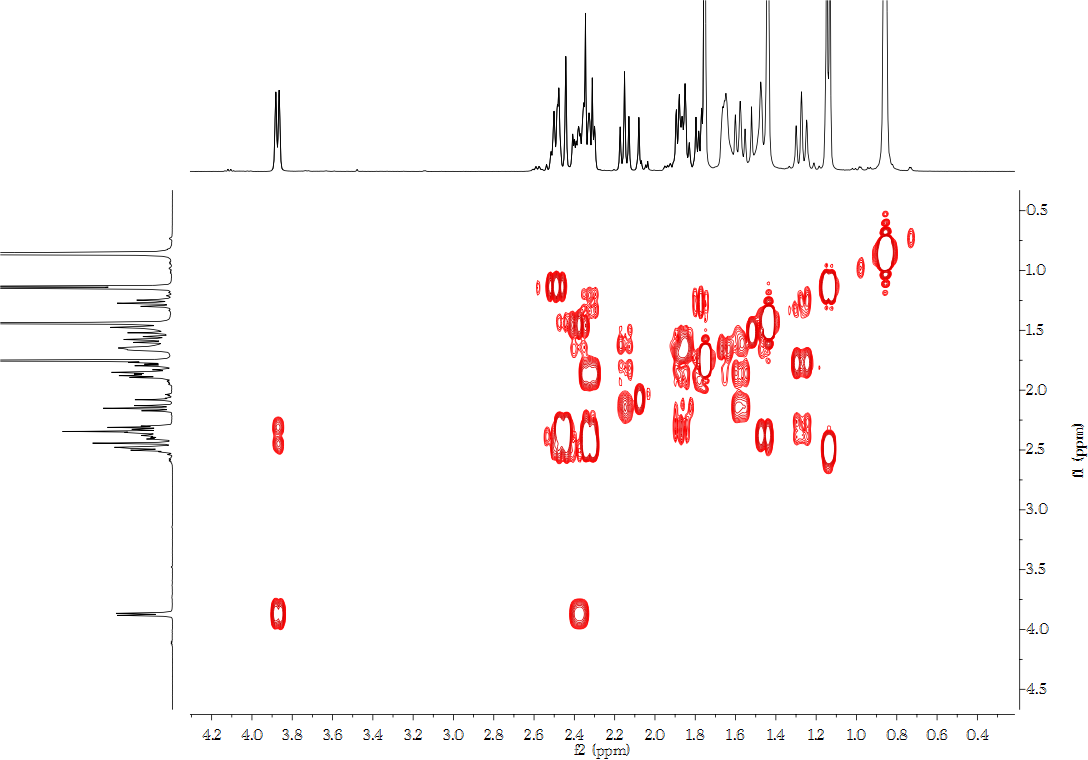


**Figure S11.** COSY (CDCl_3_) spectrum of compound **2**


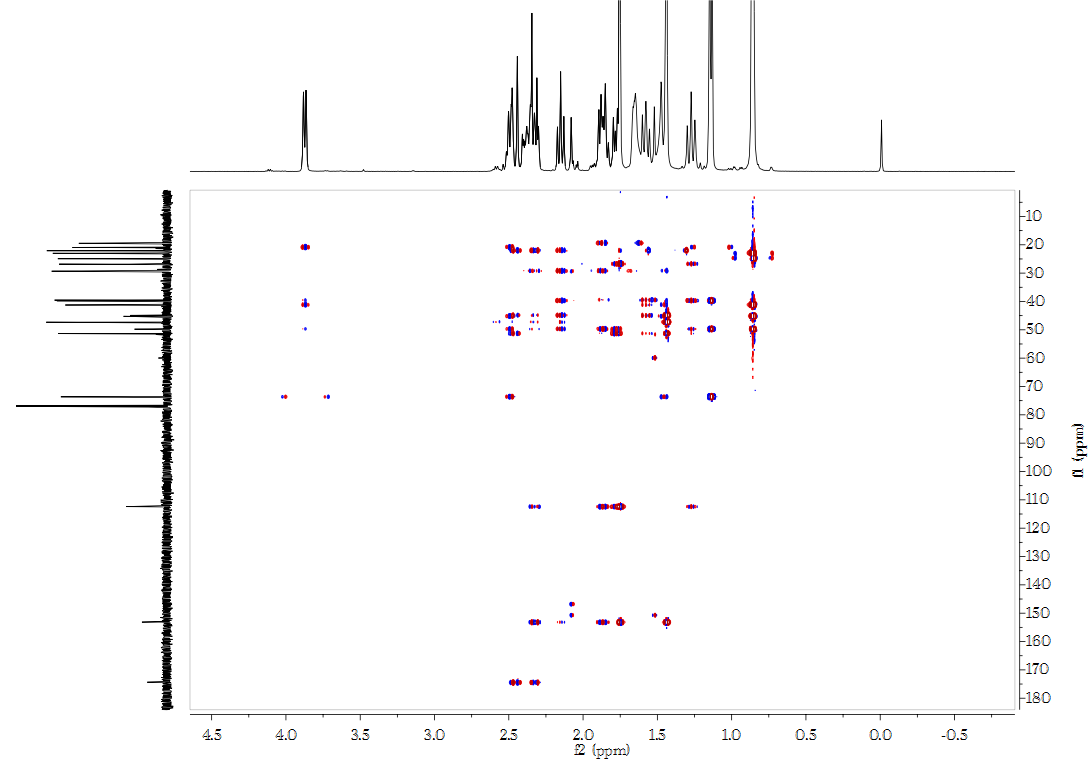


**Figure S12.** HMBC (CDCl_3_) spectrum of compound **2**


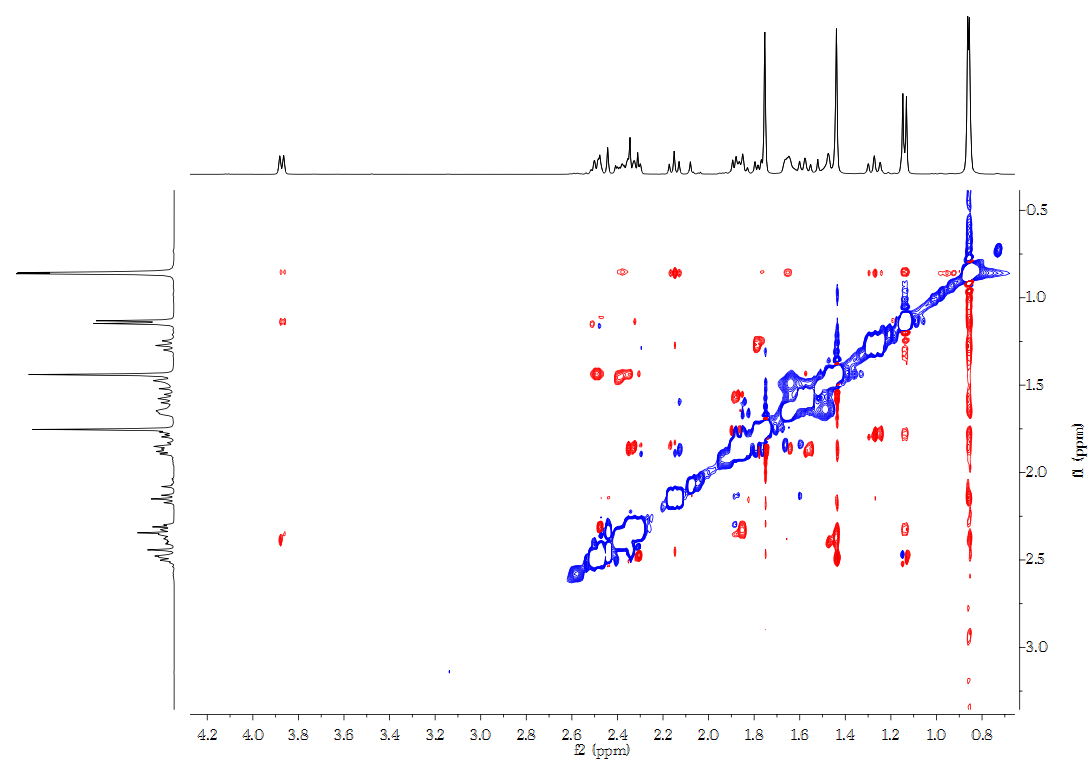


**Figure S13.** NOESY (CDCl_3_) spectrum of compound **2**


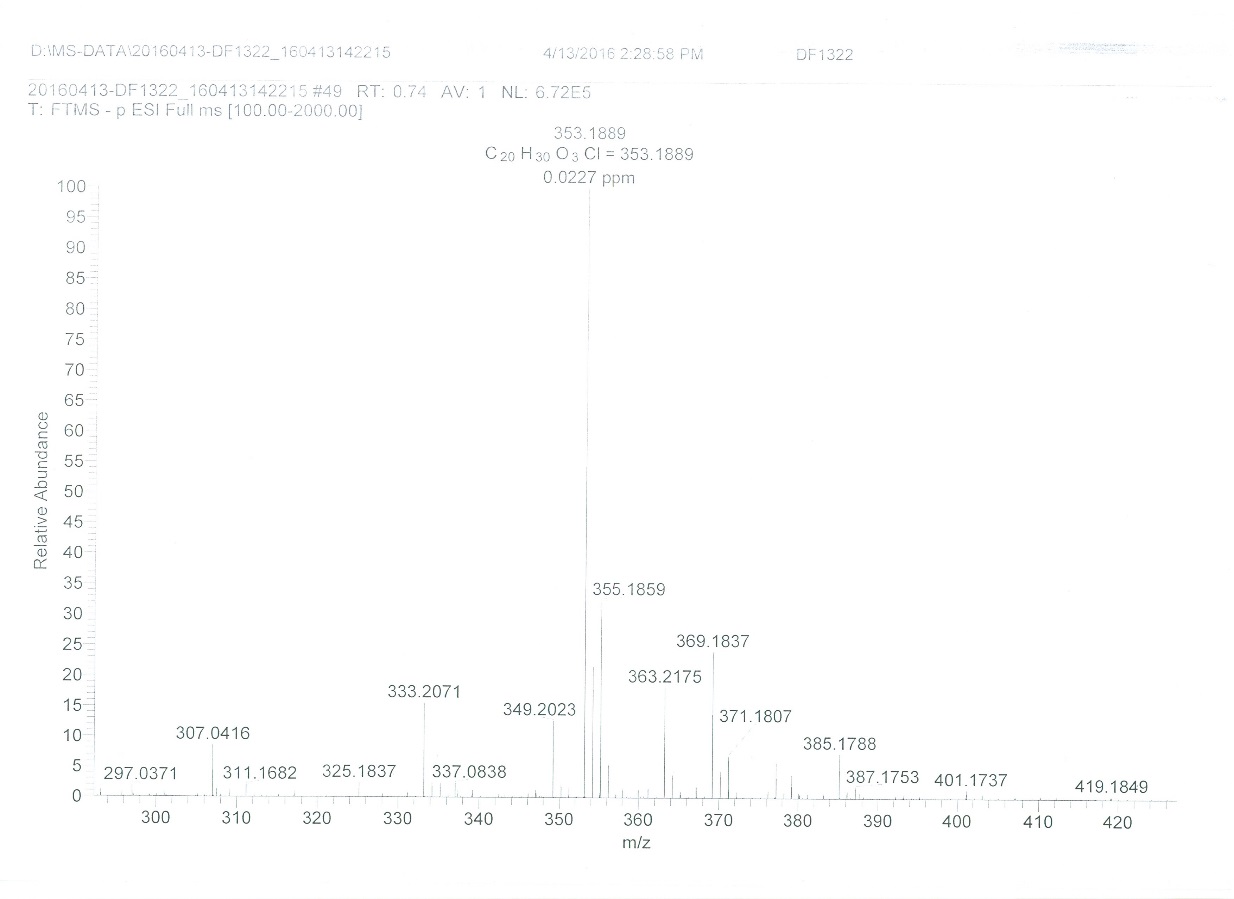


**Figure S14.** HRESIMS spectrum of compound **2**


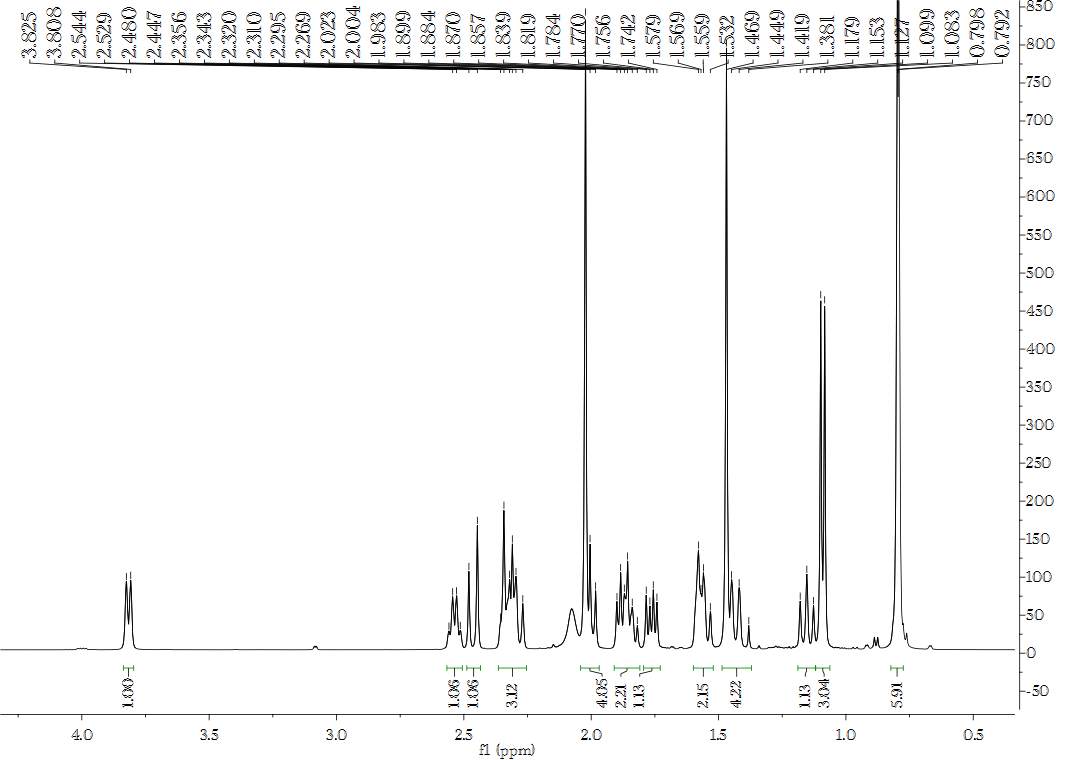


**Figure S15.** ^1^H NMR (500 MHz, CDCl_3_) spectrum of compound **3**


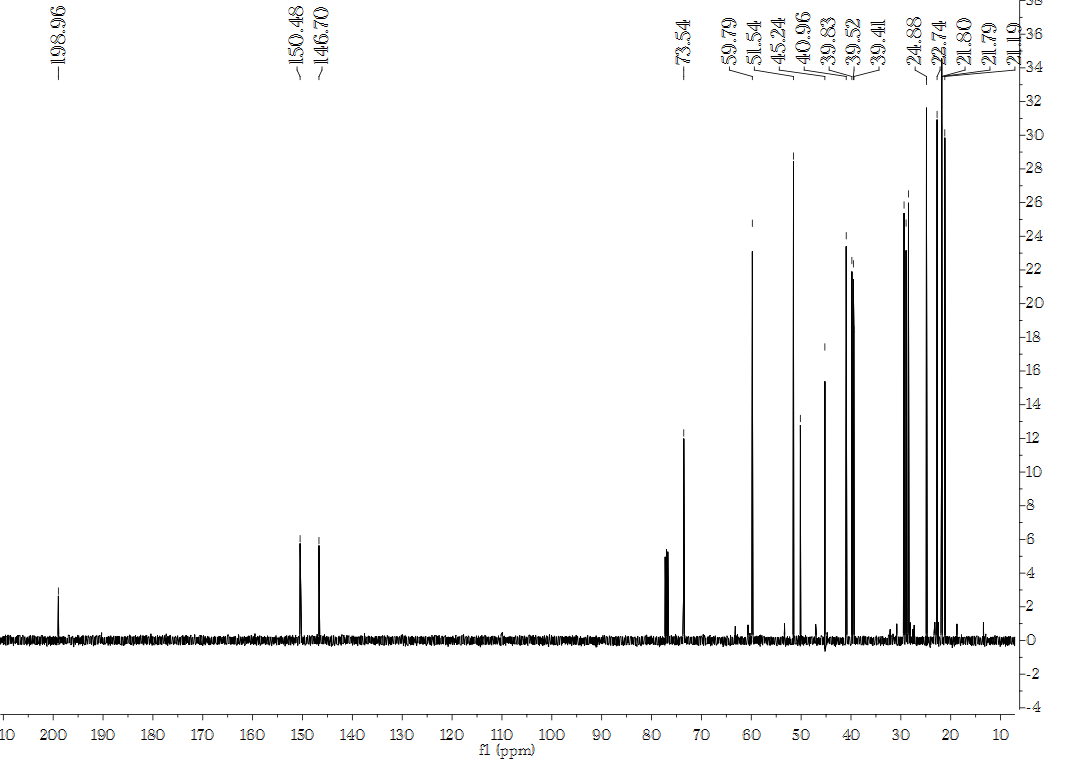


**Figure S16.** ^13^C NMR (125 MHz, CDCl_3_) spectrum of compound **3**


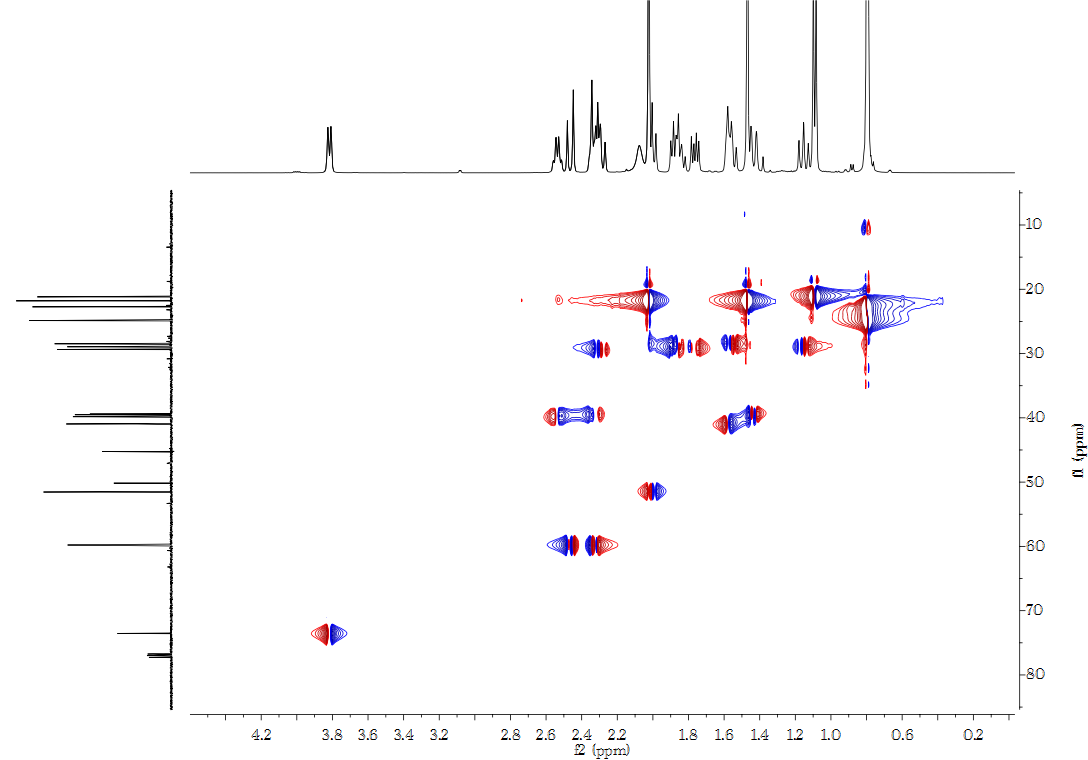


**Figure S17.** HMQC (CDCl_3_) spectrum of compound **3**


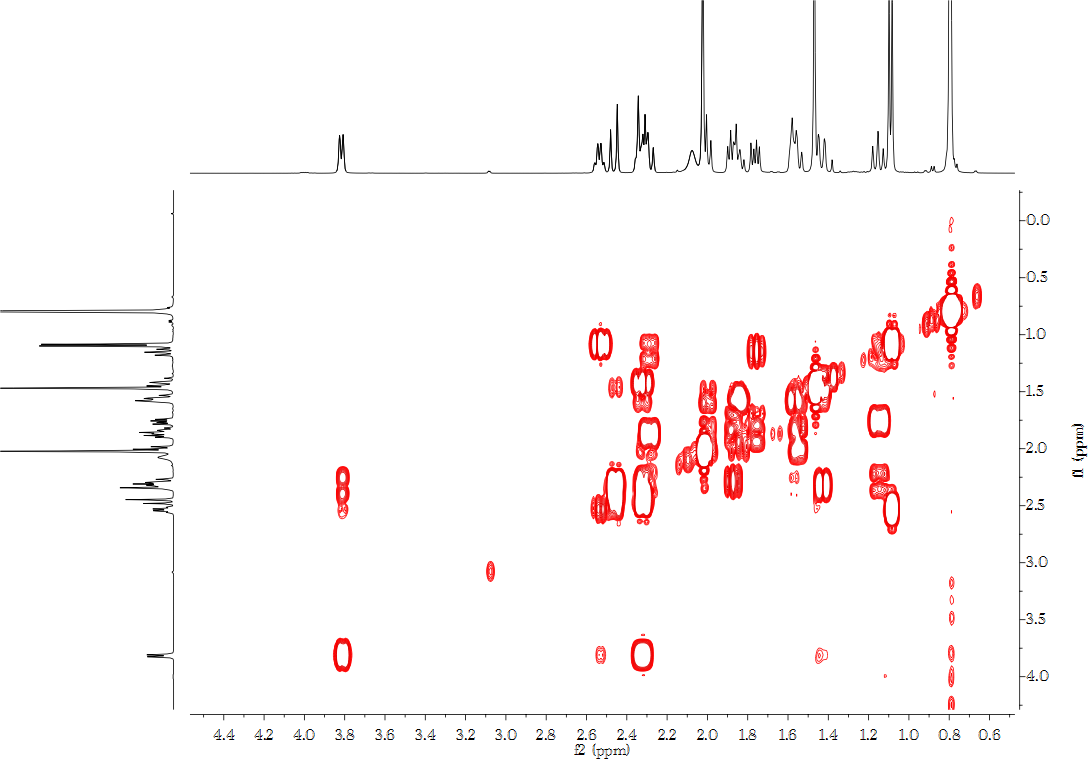


**Figure S18.** COSY (CDCl_3_) spectrum of compound **3**


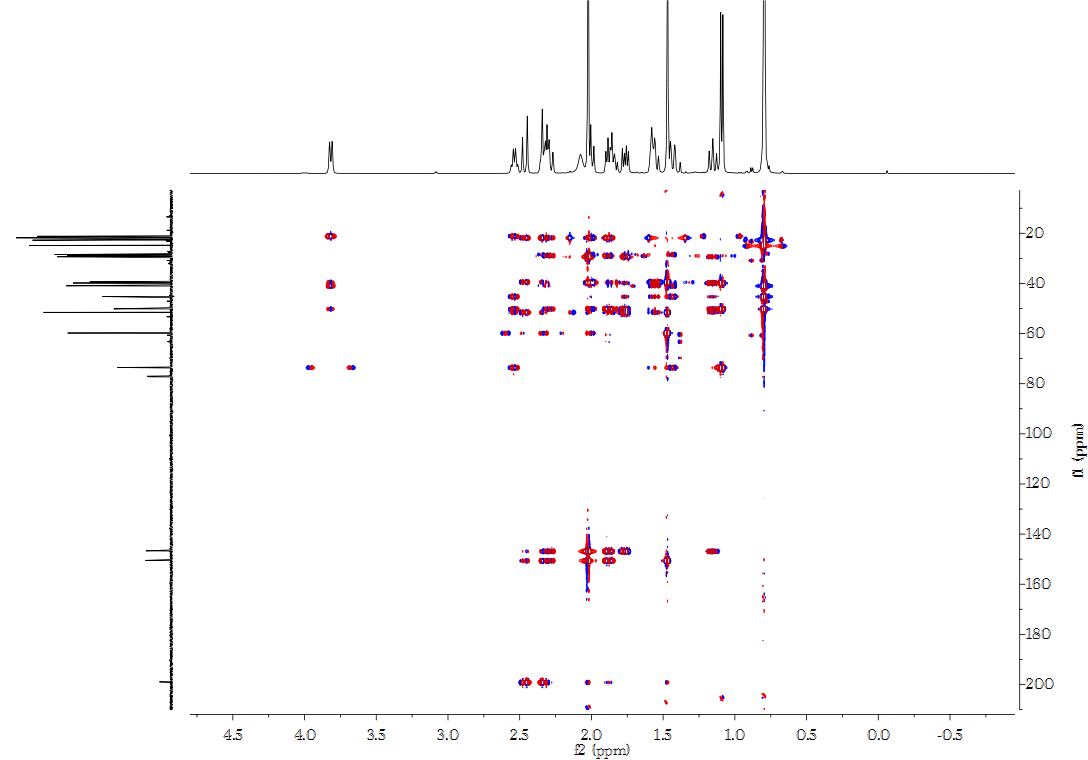


**Figure S19.** HMBC (CDCl_3_) spectrum of compound **3**


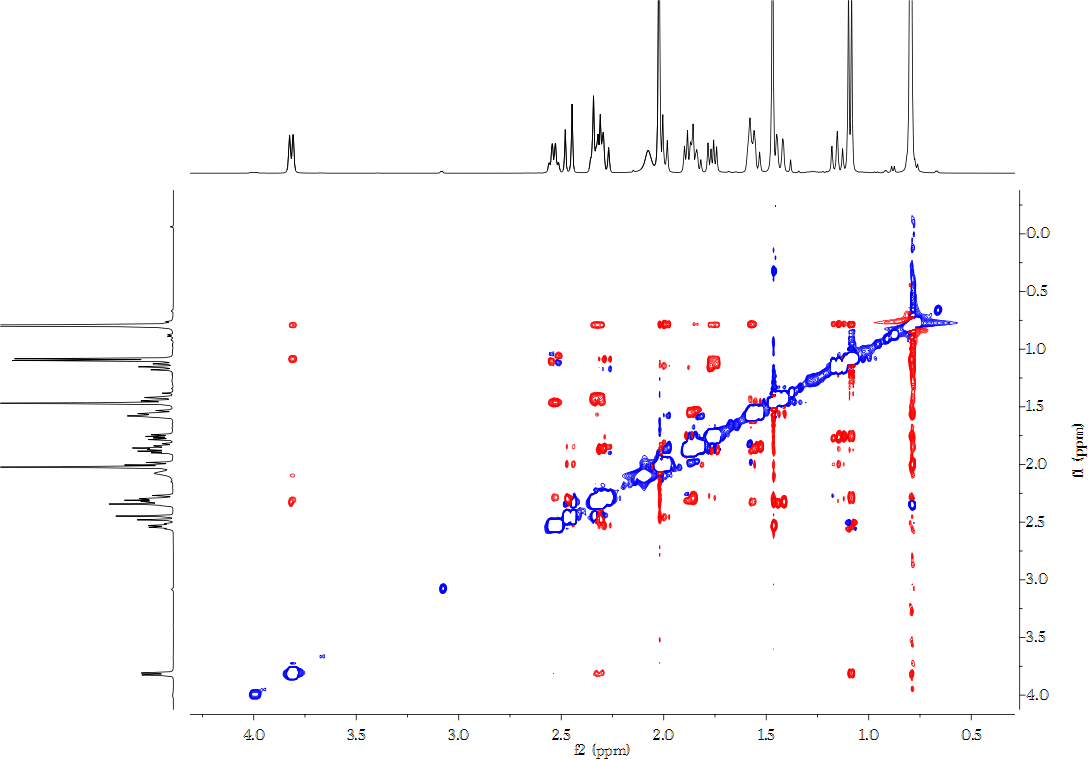


**Figure S20.** NOESY (CDCl_3_) spectrum of compound **3**


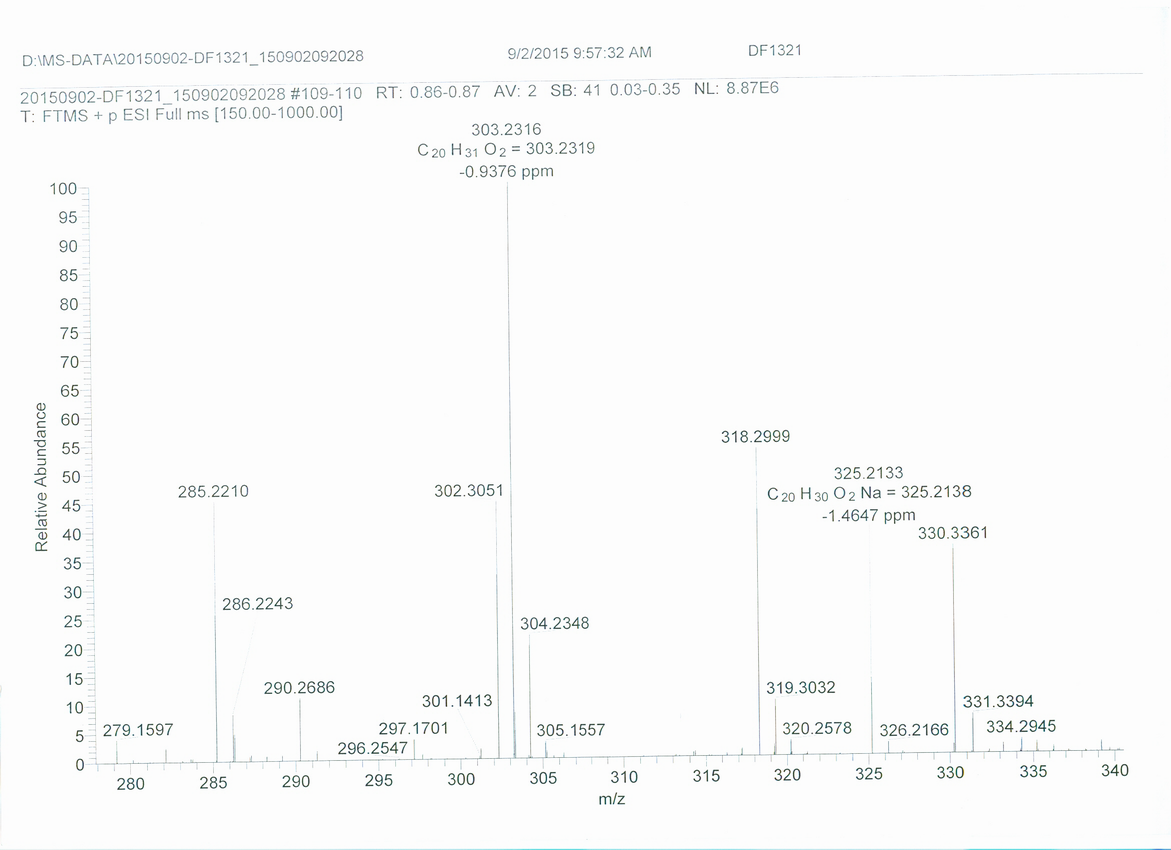


**Figure S21.** HRESIMS spectrum of compound **3**


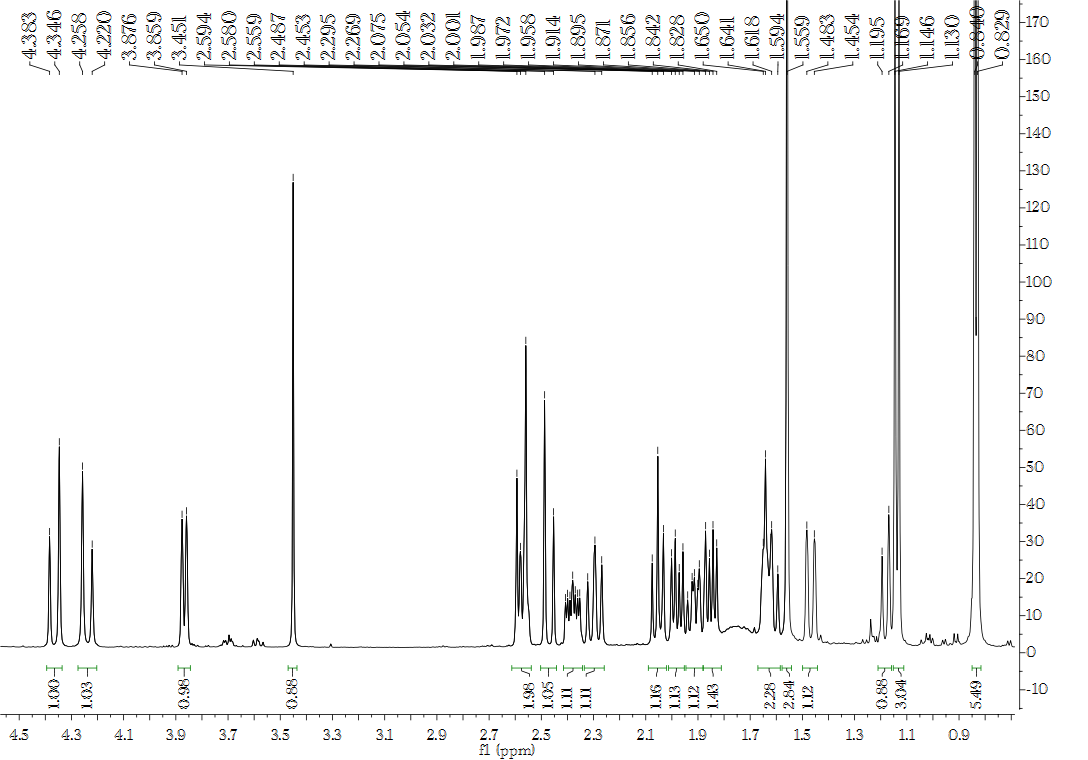


**Figure S22.** ^1^H NMR (500 MHz, CDCl_3_) spectrum of compound **4**


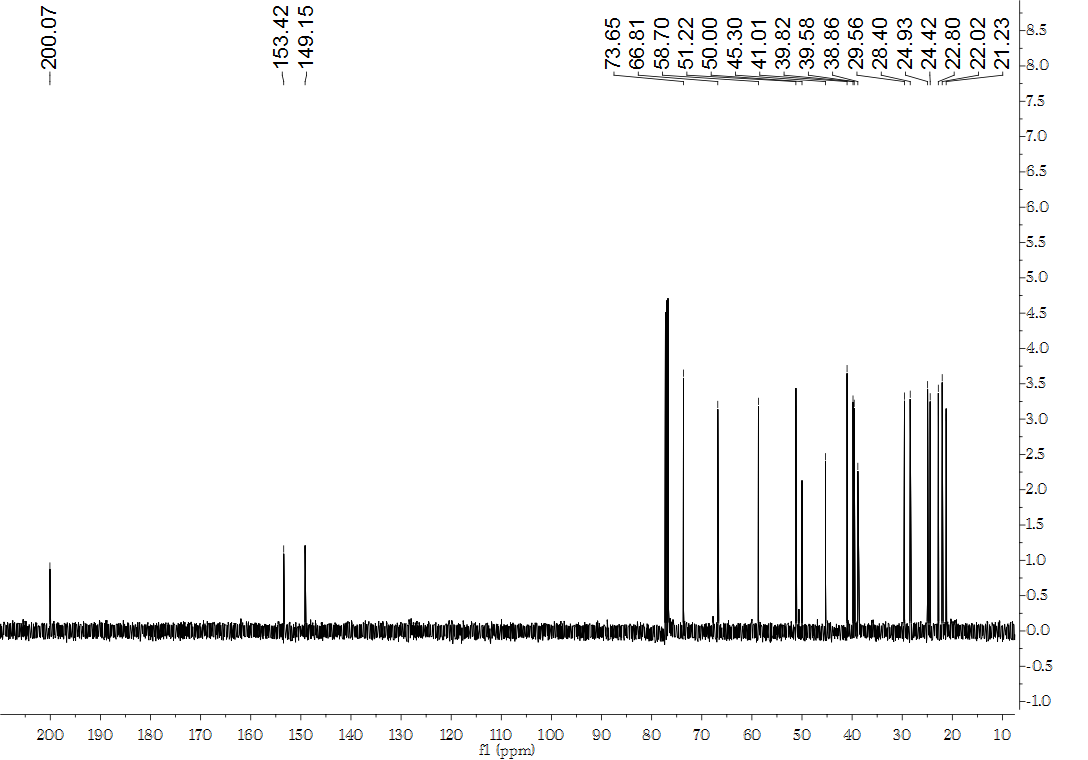


**Figure S23.** ^13^C NMR (125 MHz, CDCl_3_) spectrum of compound **4**


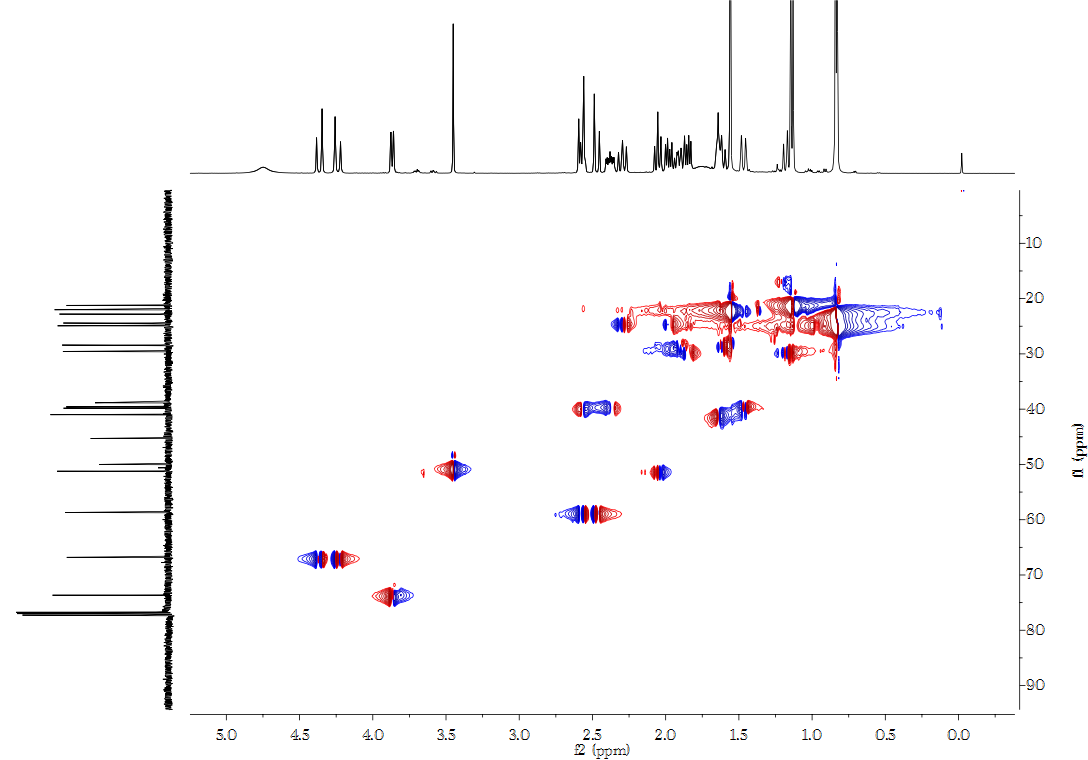


**Figure S24.** HMQC (CDCl_3_) spectrum of compound **4**


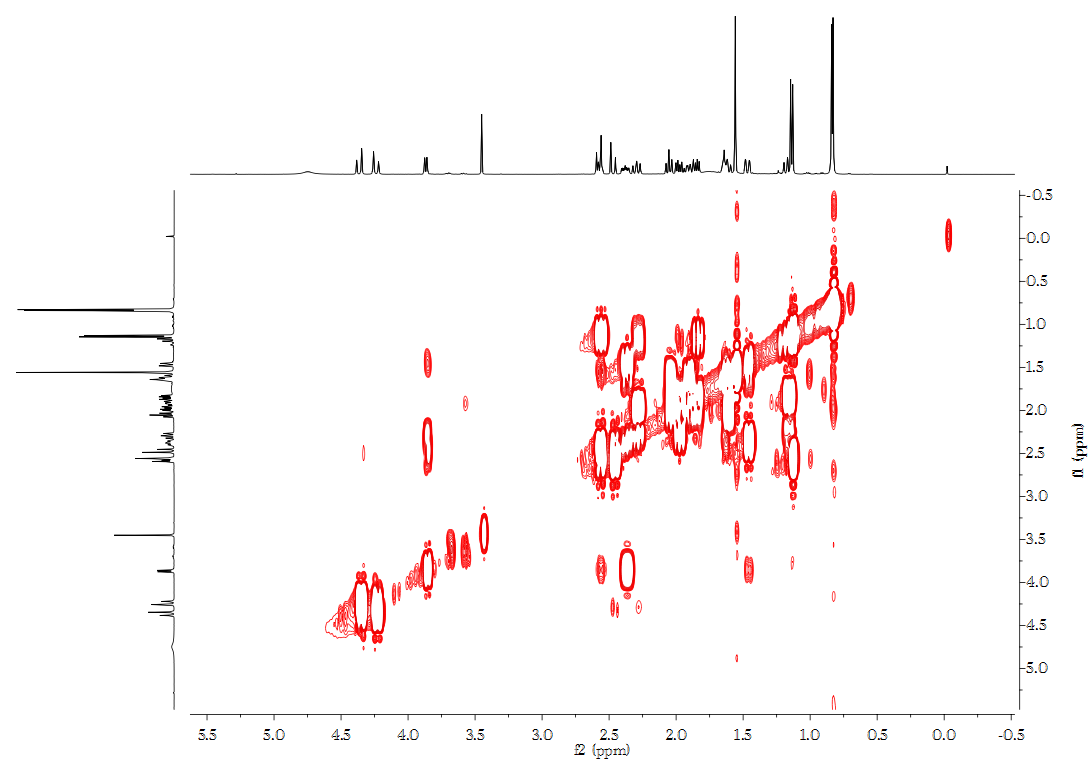


**Figure S25.** COSY (CDCl_3_) spectrum of compound **4**


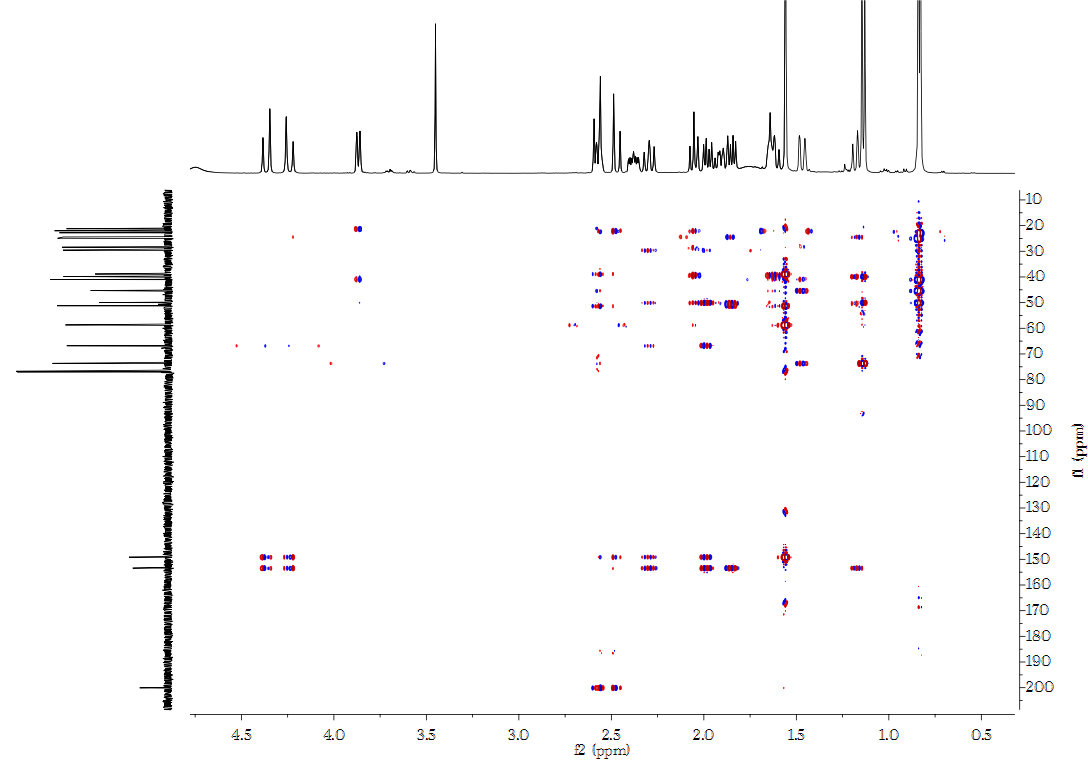


**Figure S26.** HMBC spectrum of compound **4**


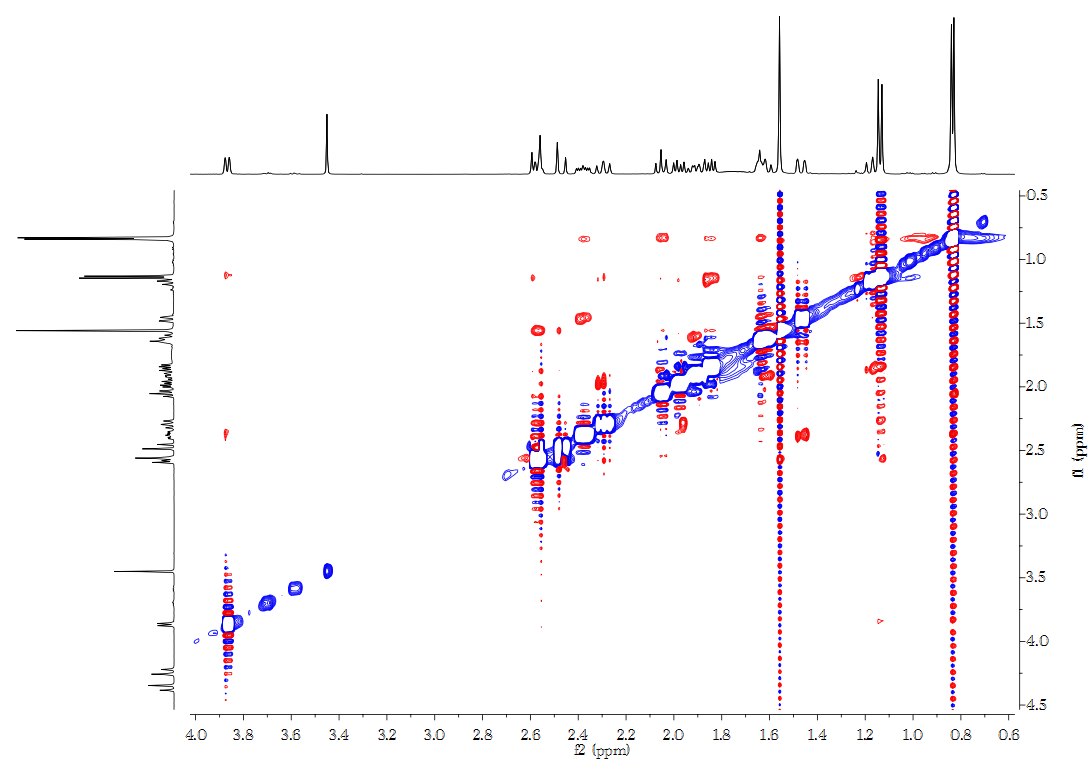


**Figure S27.** NOESY (CDCl_3_) spectrum of compound **4**


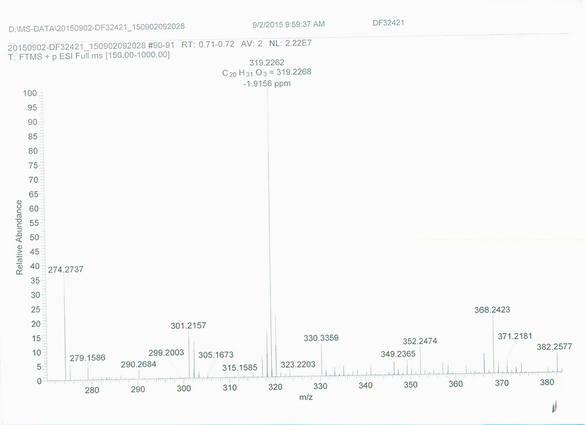


**Figure S28.** HRESIMS spectrum of compound **4**


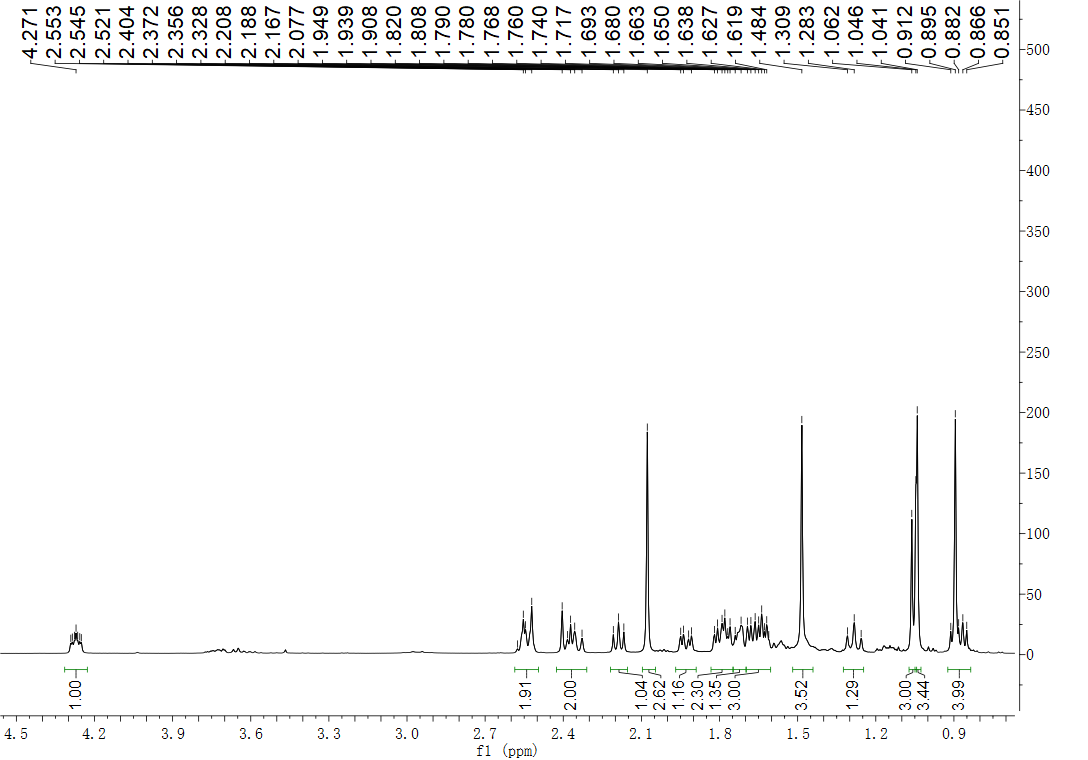


**Figure S29.** ^1^H NMR (500 MHz, CDCl_3_) spectrum of compound **5**


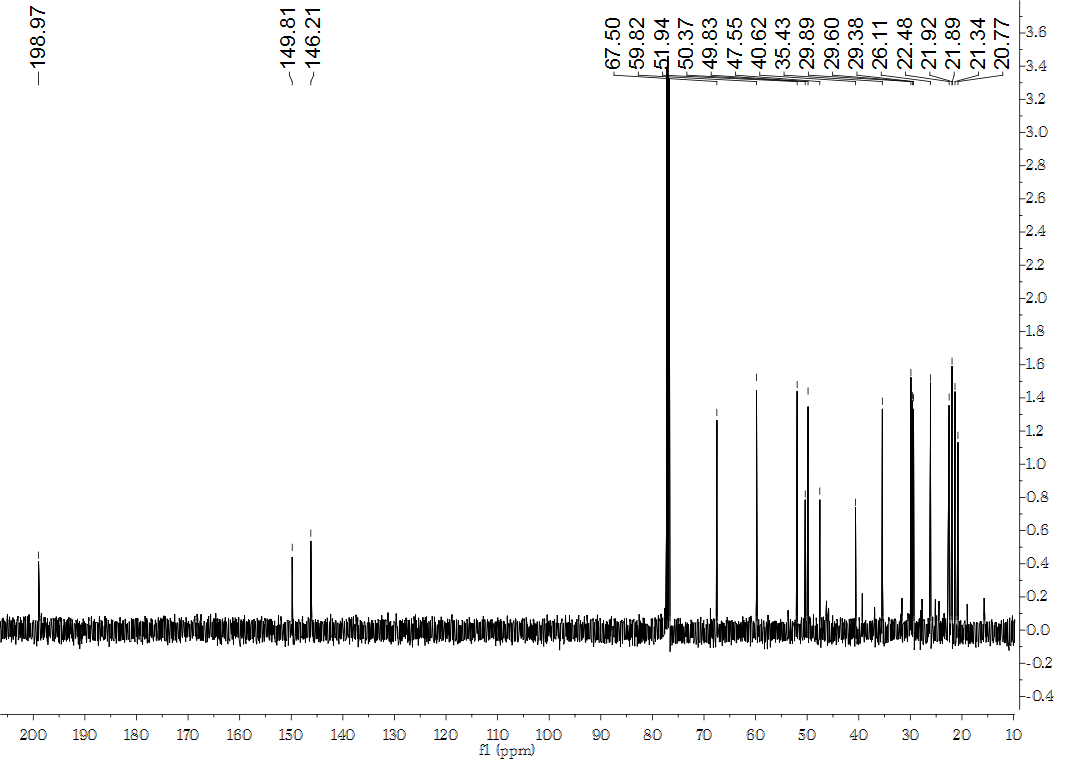


**Figure S30.** ^13^C NMR (125 MHz, CDCl_3_) spectrum of compound **5**


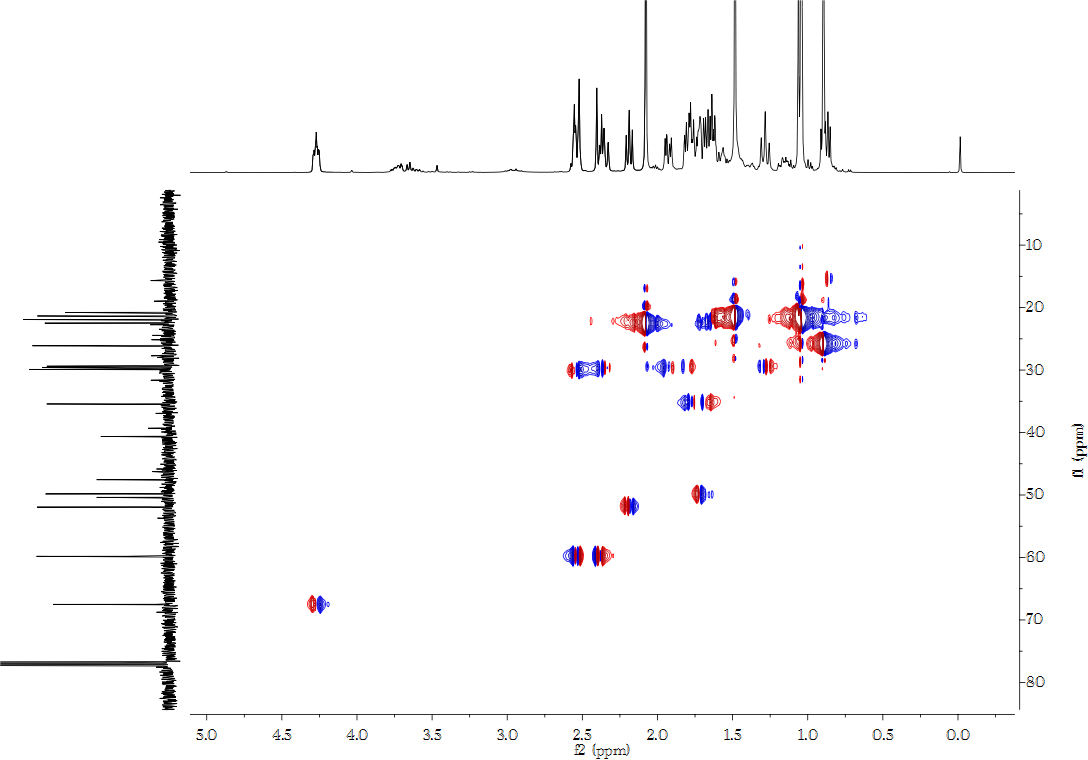


**Figure S31.** HMQC (CDCl_3_) spectrum of compound **5**


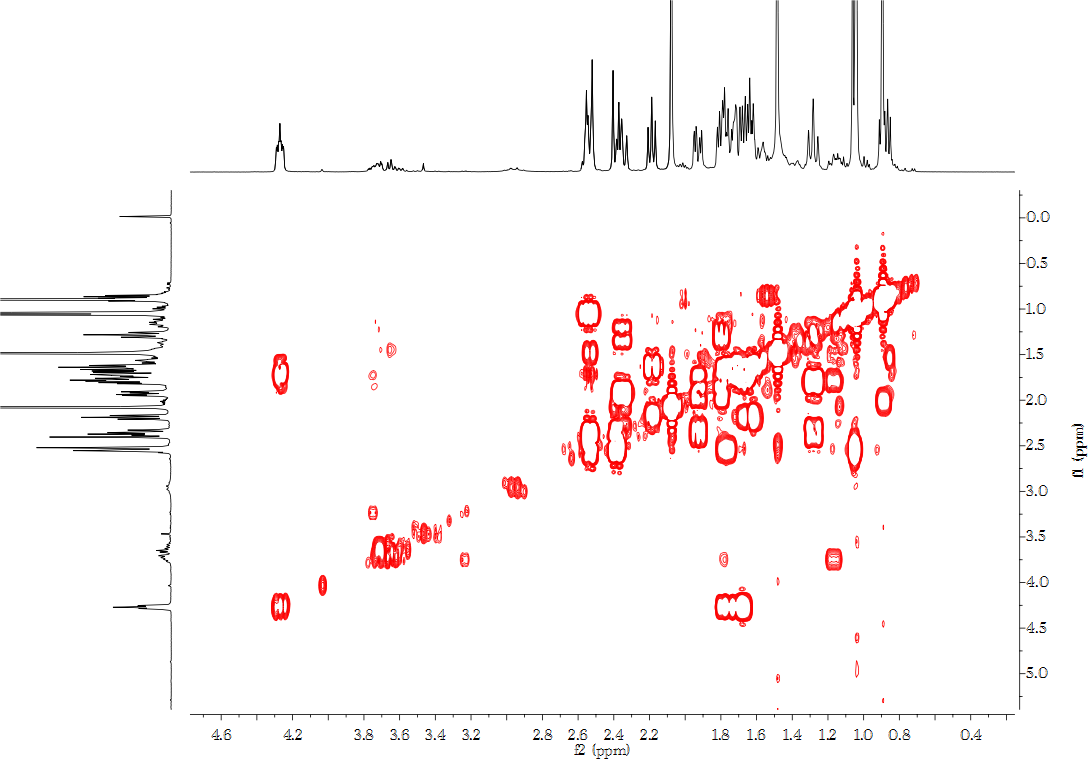


**Figure S32.** COSY (CDCl_3_) spectrum of compound **5**


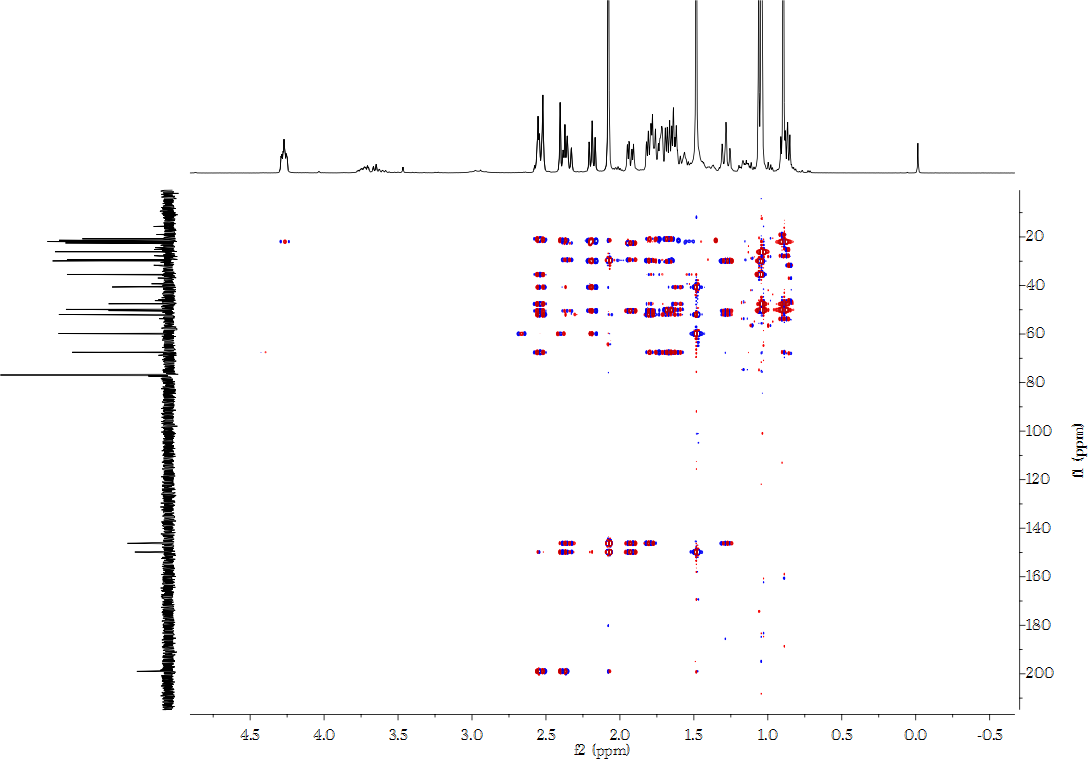


**Figure S33.** HMBC (CDCl_3_) spectrum of compound **5**


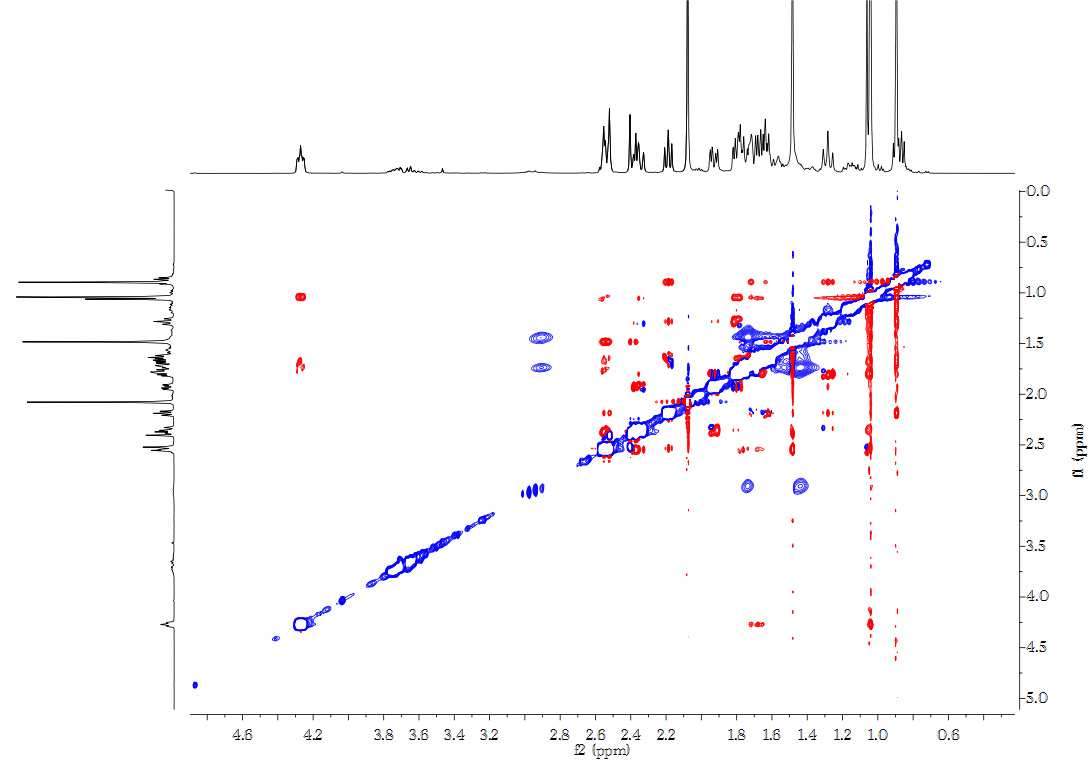


**Figure S34.** NOESY (CDCl_3_) spectrum of compound **5**

**
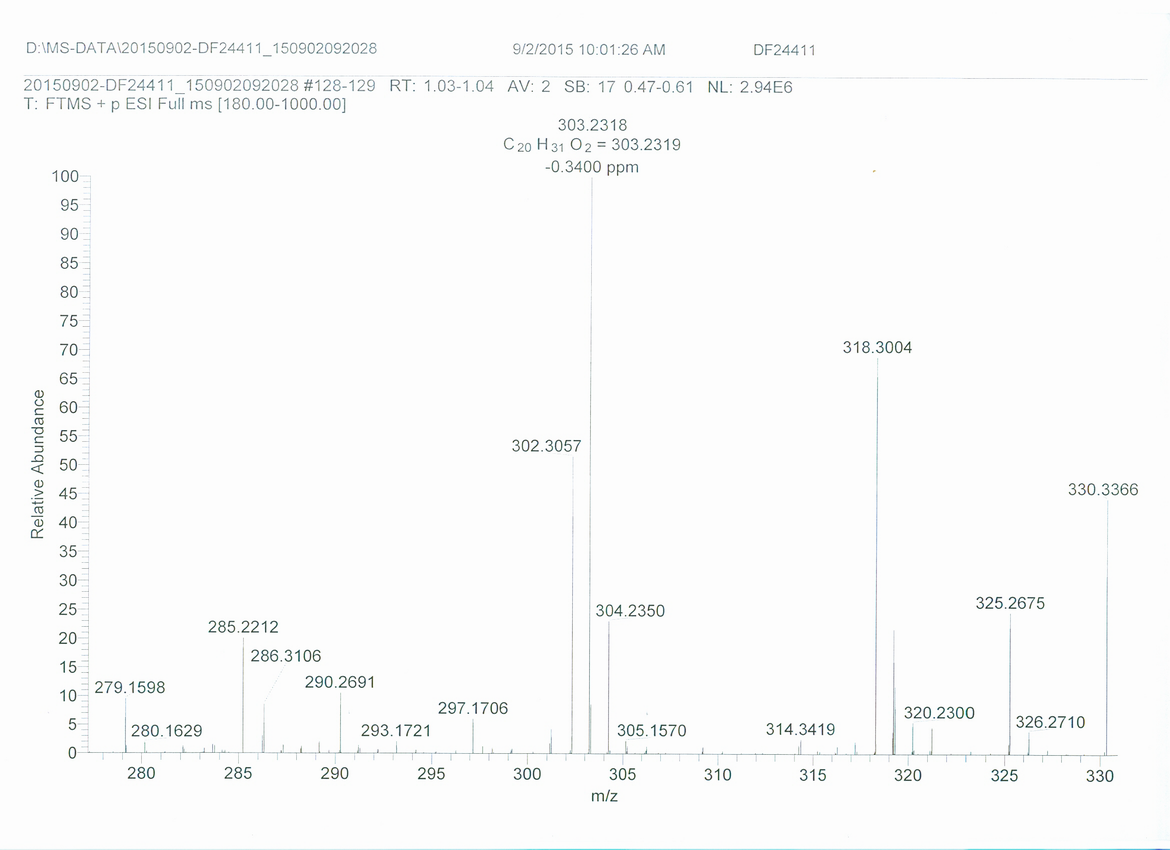
**

**Figure S35.** HRESIMS spectrum of compound **5**

**
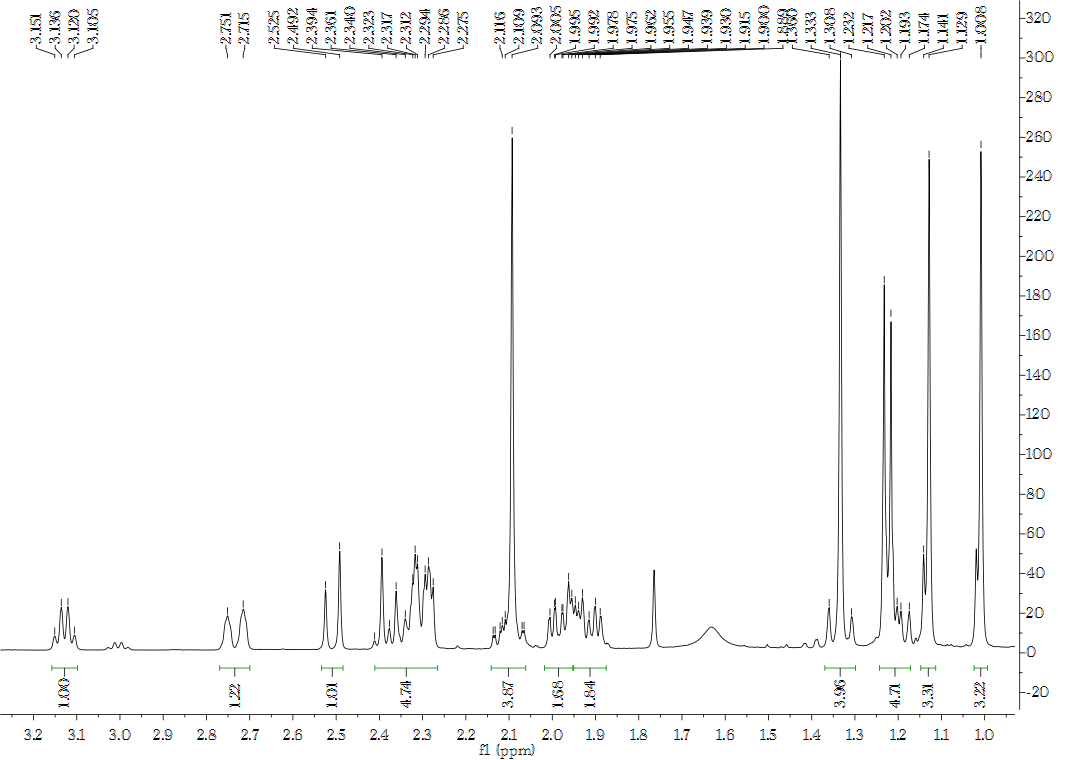
**

**Figure S36.** ^1^H NMR (500 MHz, CDCl_3_) spectrum of compound **6**


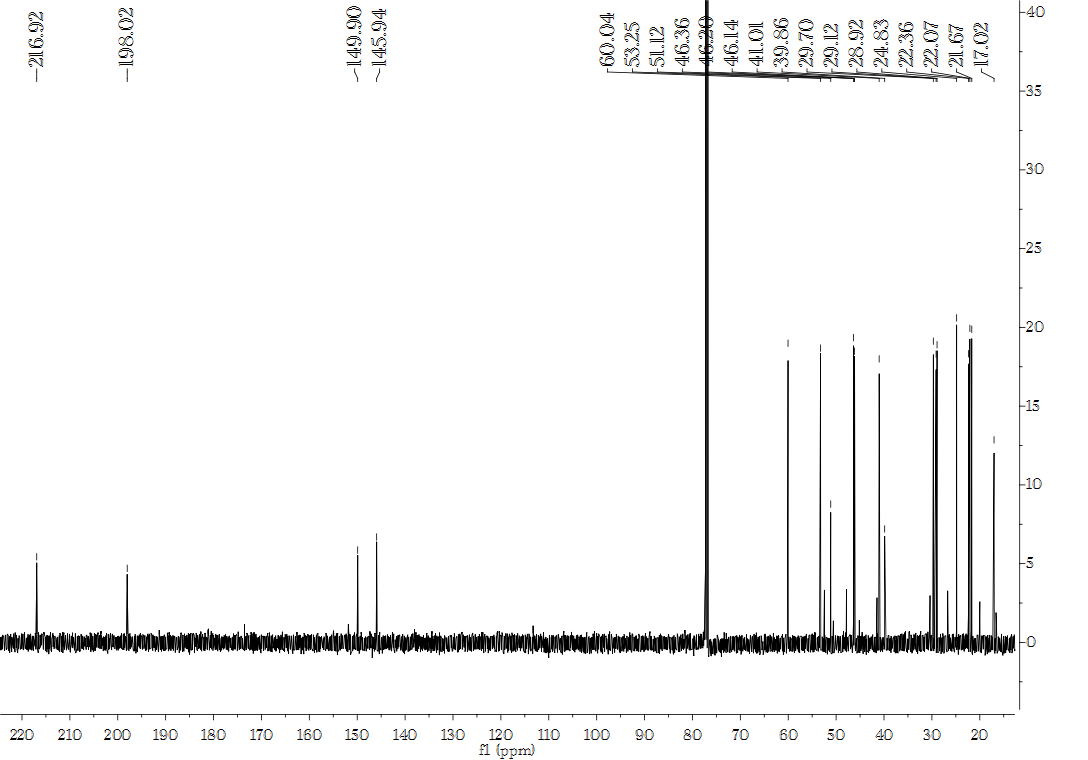


**Figure S37.** ^13^C NMR (125 MHz, CDCl_3_) spectrum of compound **6**


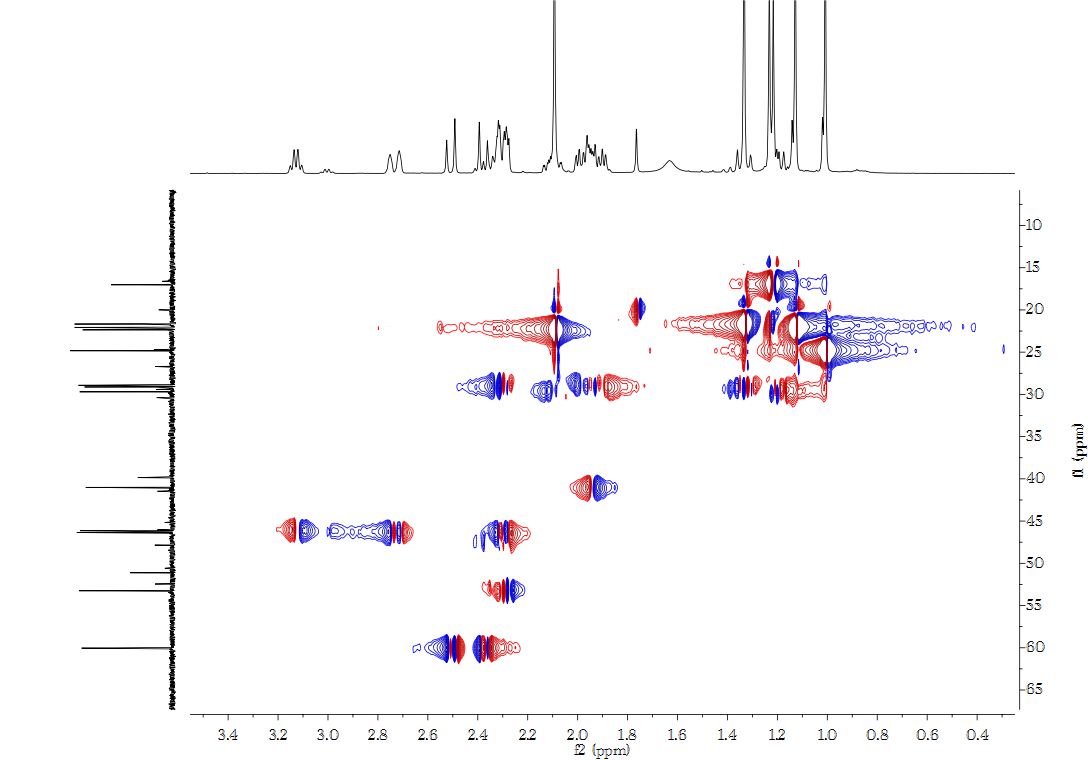


**Figure S38.** HMQC (CDCl_3_) spectrum of compound **6**

**
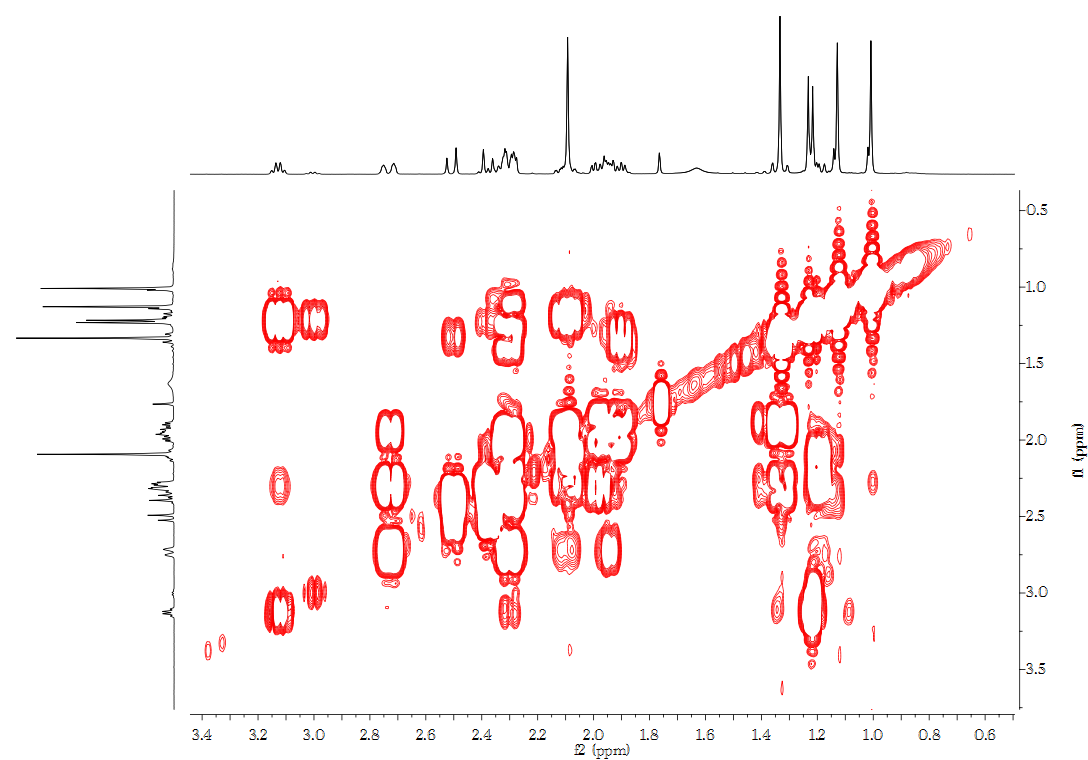
**

**Figure S39.** COSY (CDCl_3_) spectrum of compound **6**

**
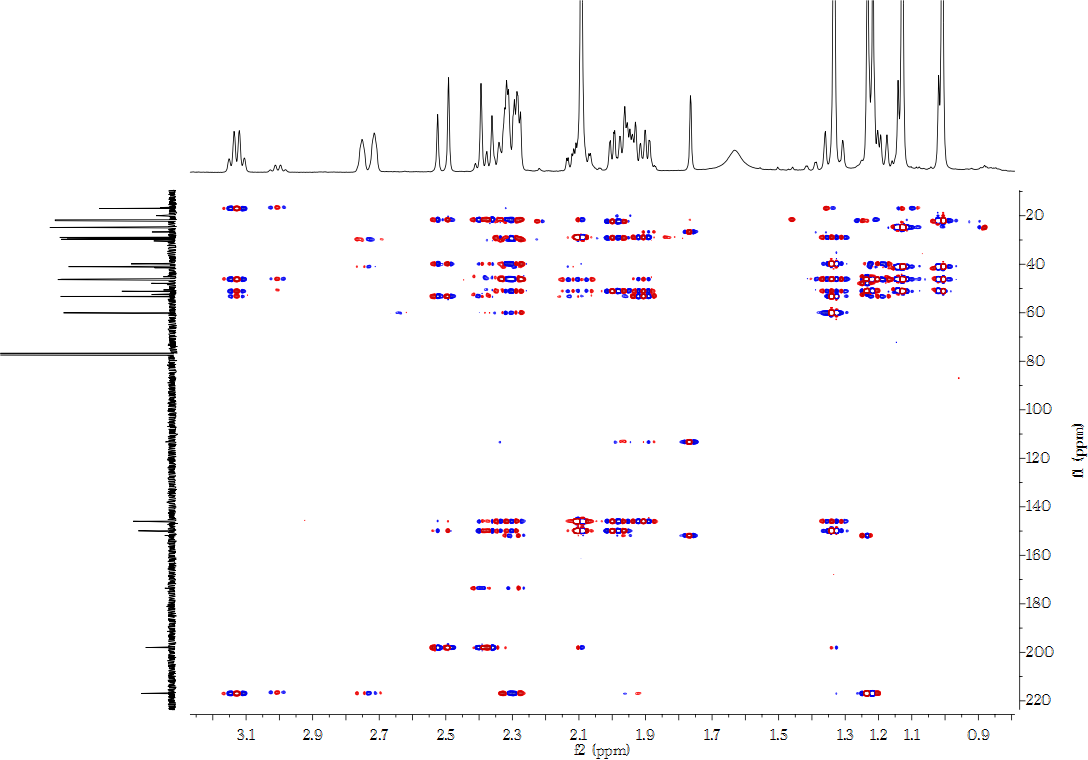
**

**Figure S40.** HMBC (CDCl_3_) spectrum of compound **6**


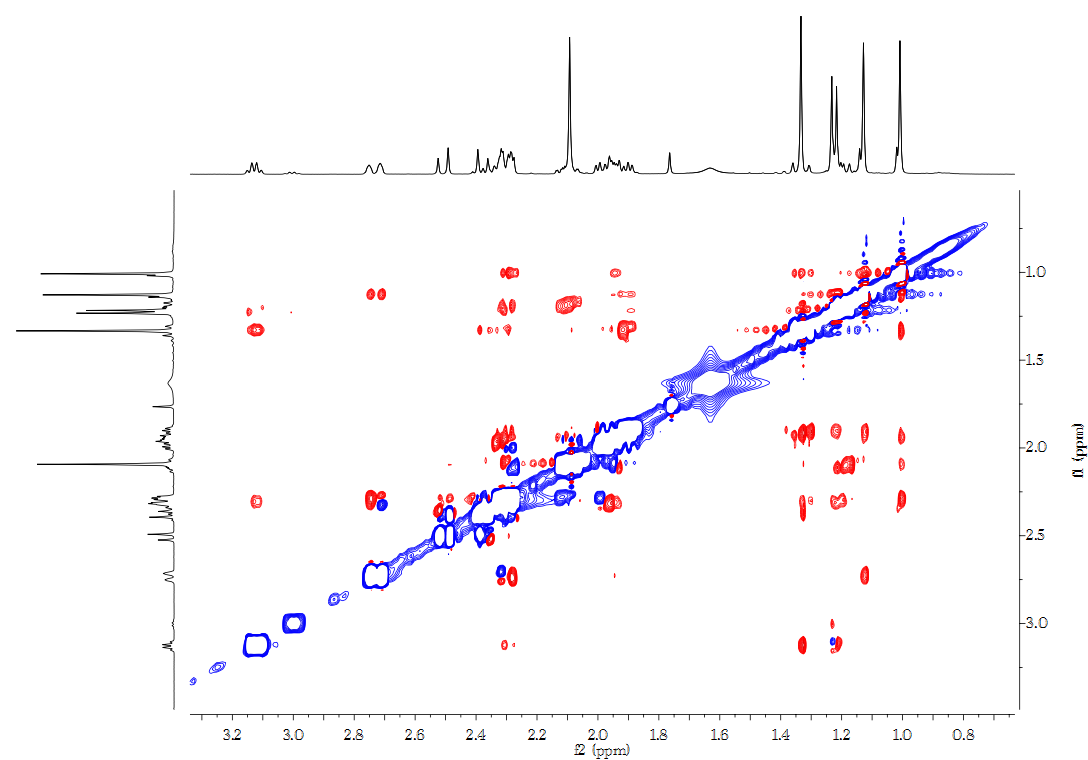


**Figure S41.** NOESY (CDCl_3_) spectrum of compound **6**


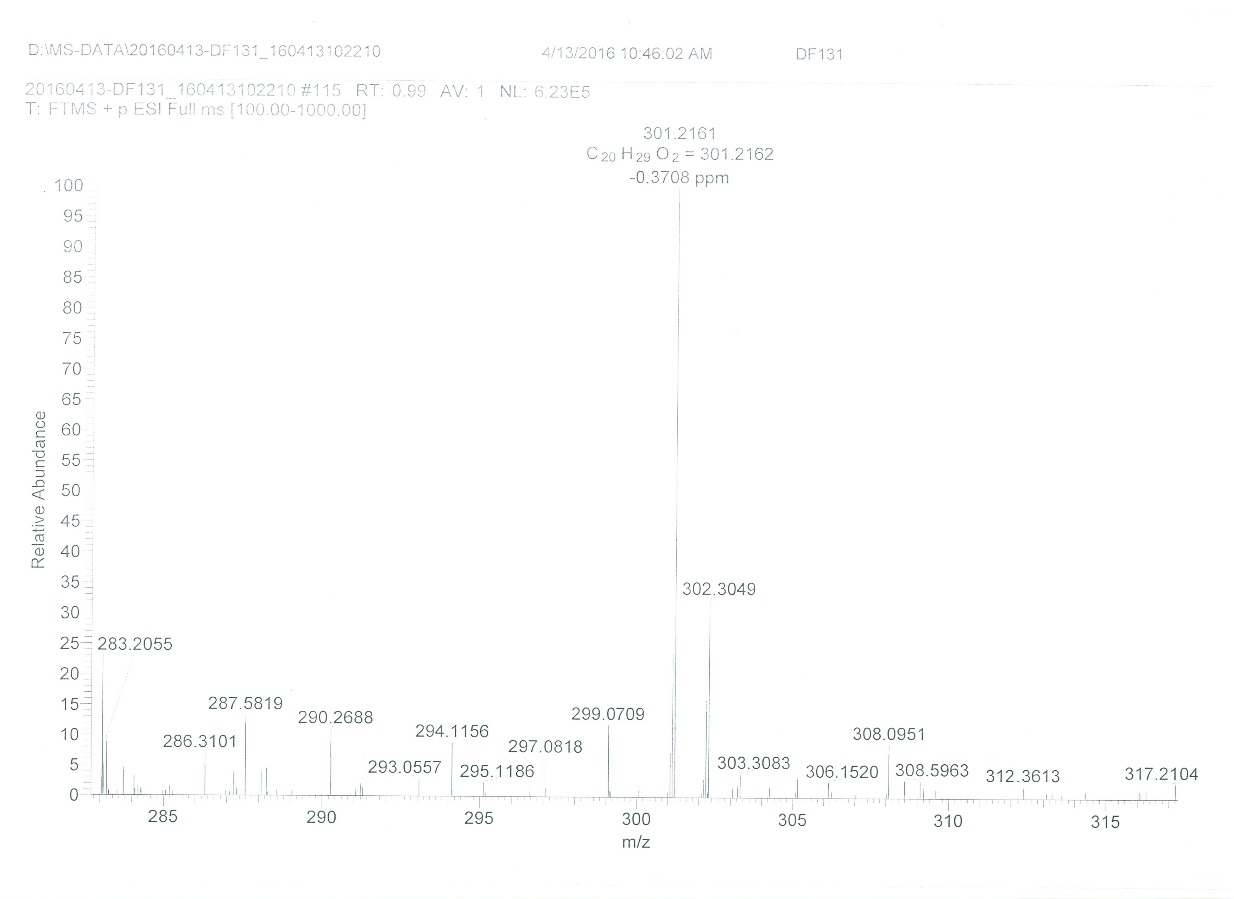


**Figure S42.** HRESIMS spectrum of compound **6**


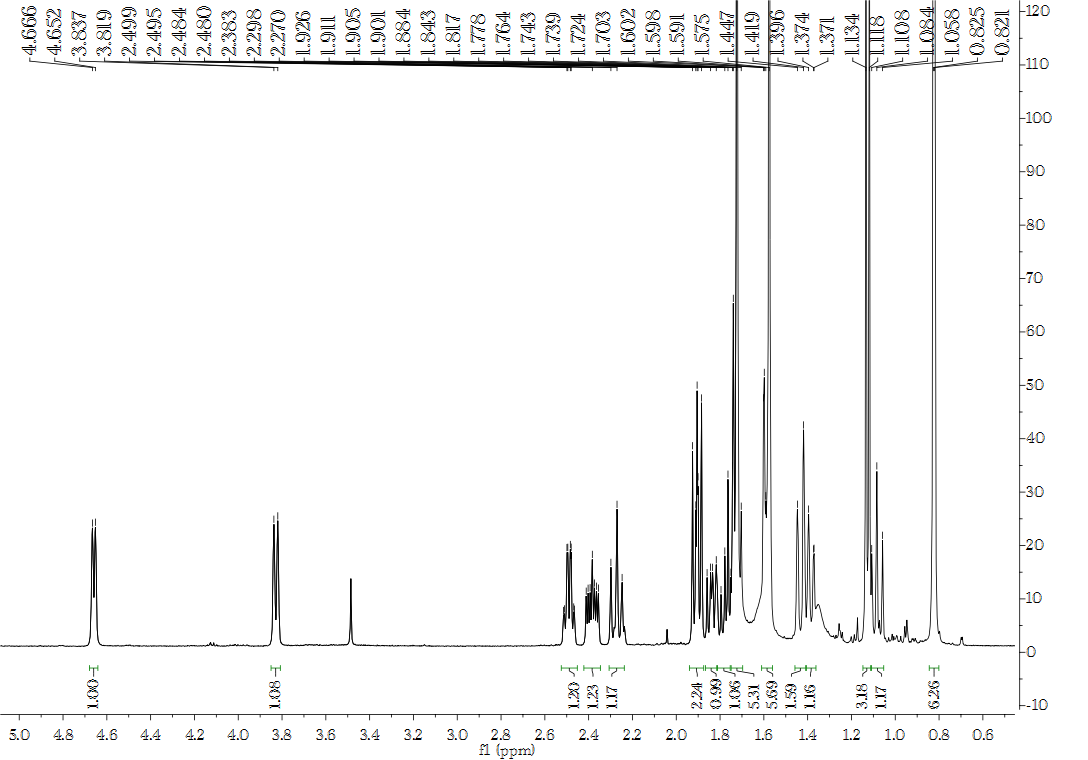


**Figure S43.** ^1^H NMR (500 MHz, CDCl_3_) spectrum of compound **7**


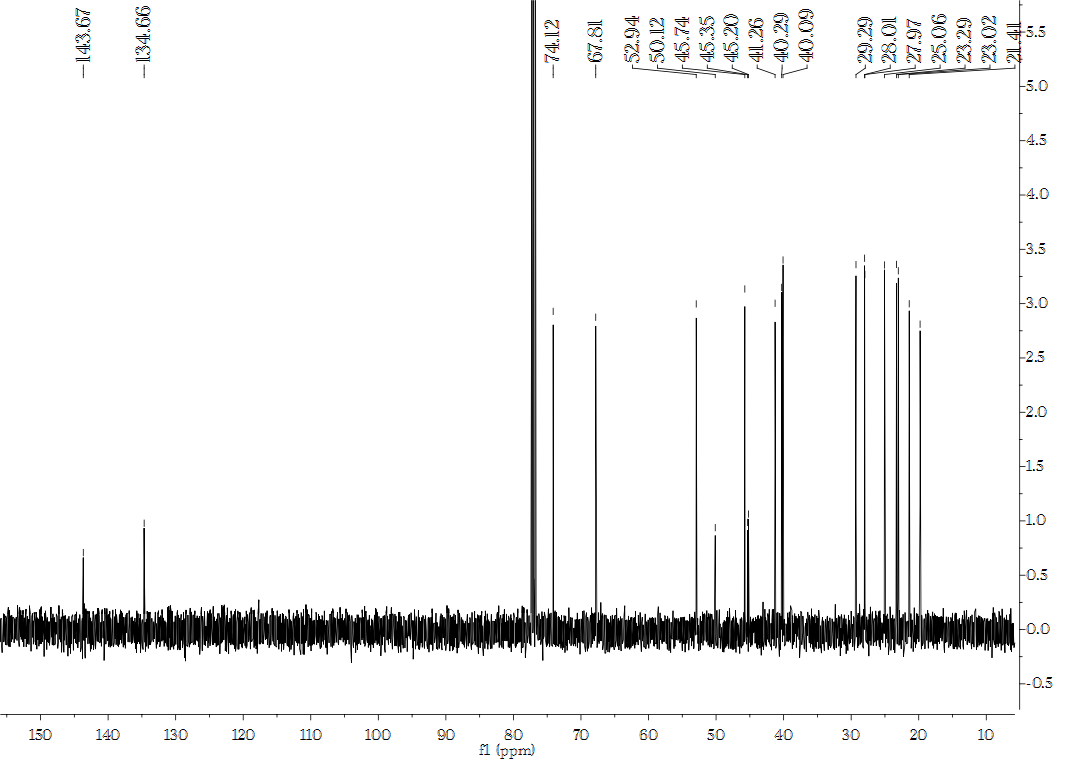


**Figure S44.** ^13^C NMR (125 MHz, CDCl_3_) spectrum of compound **7**


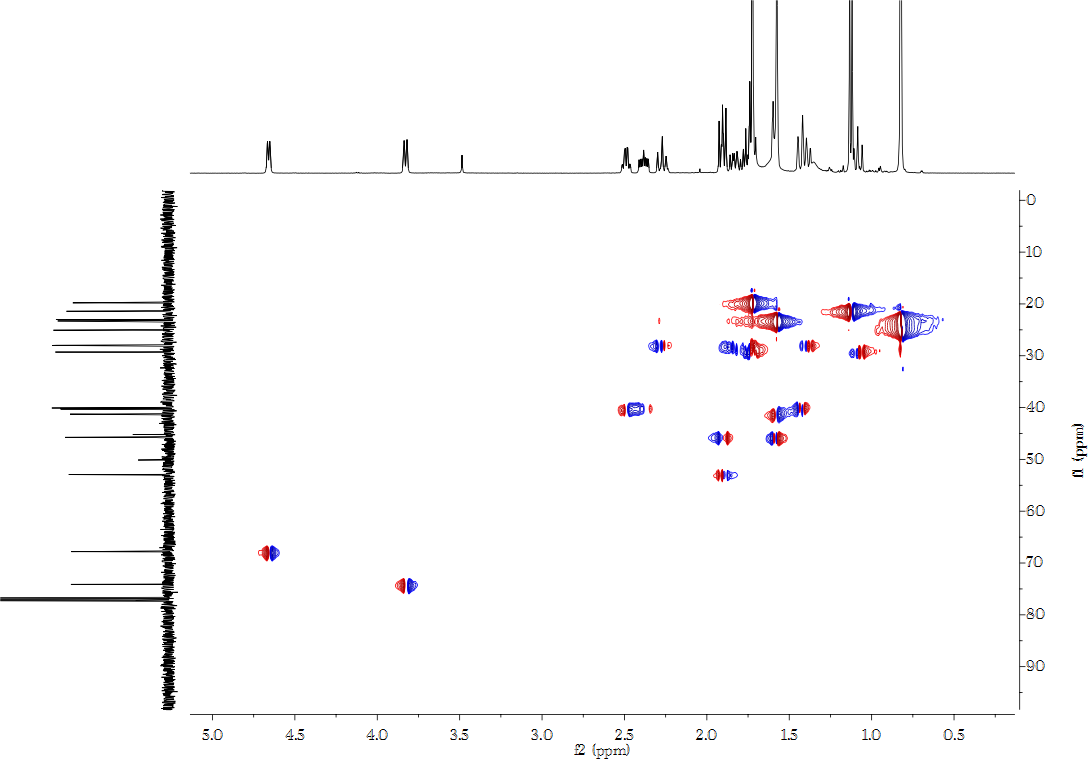


**Figure 45.** HMQC (CDCl_3_) spectrum of compound **7**


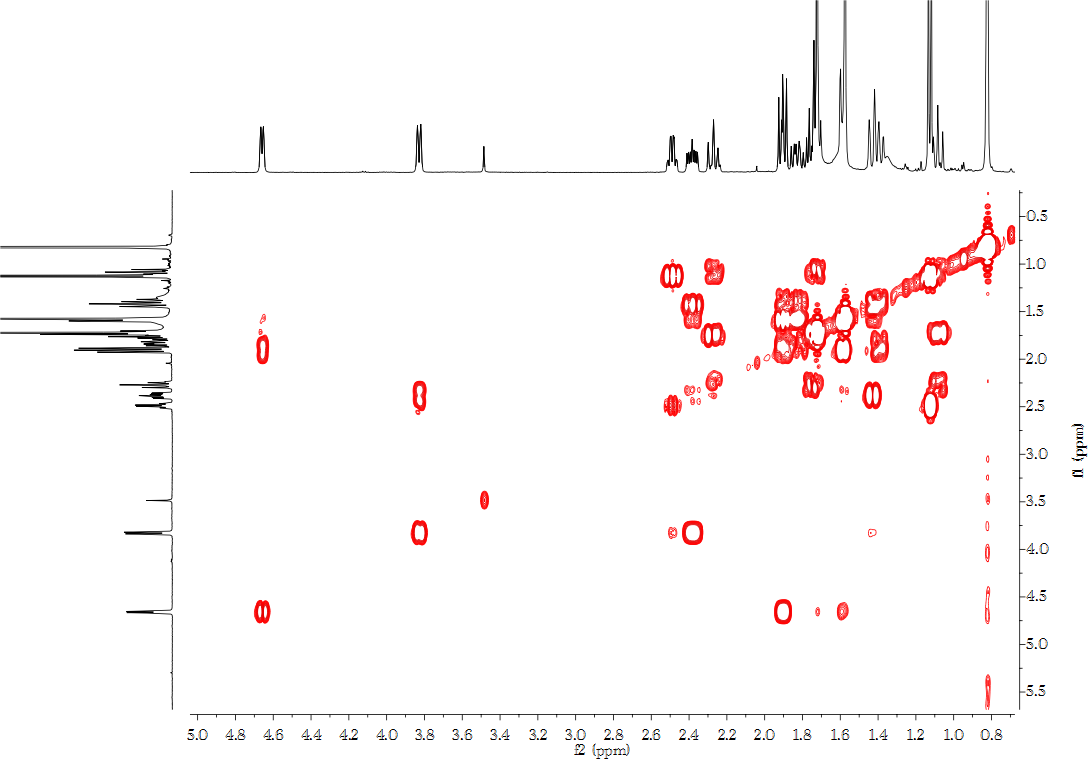


**Figure S46.** COSY spectrum of compound **7**


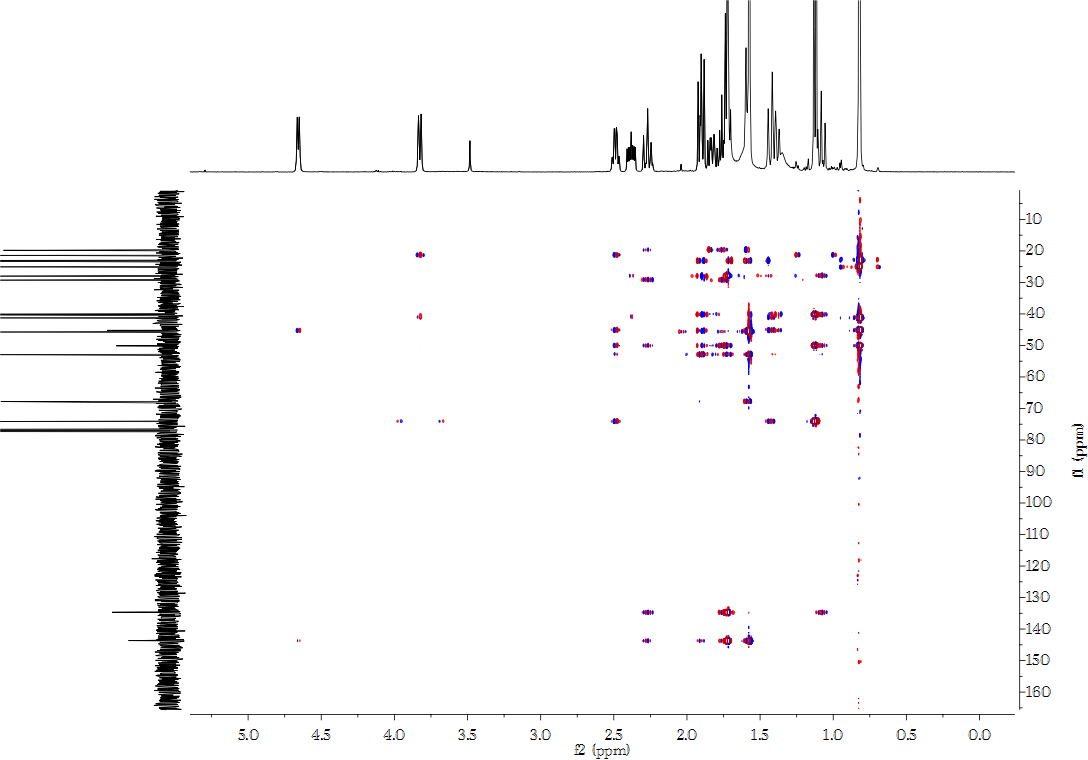


**Figure S47.** HMBC (CDCl_3_) spectrum of compound **7**


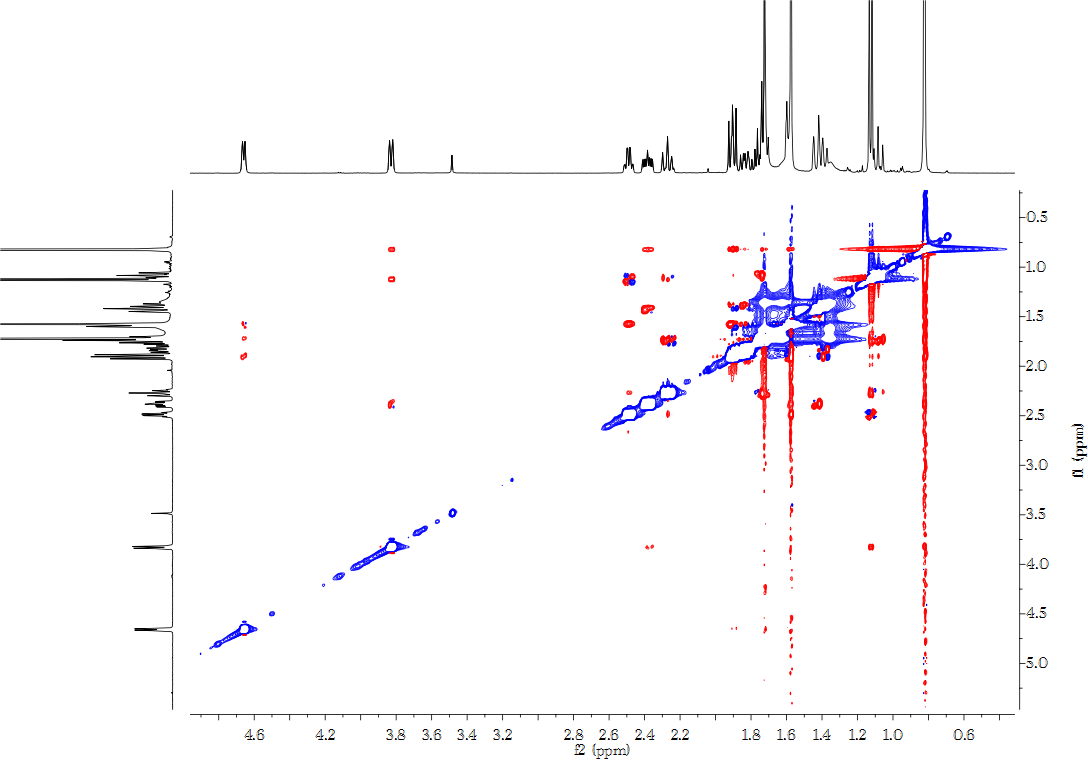


**Figure S48.** NOESY (CDCl_3_) spectrum of compound **7**

**
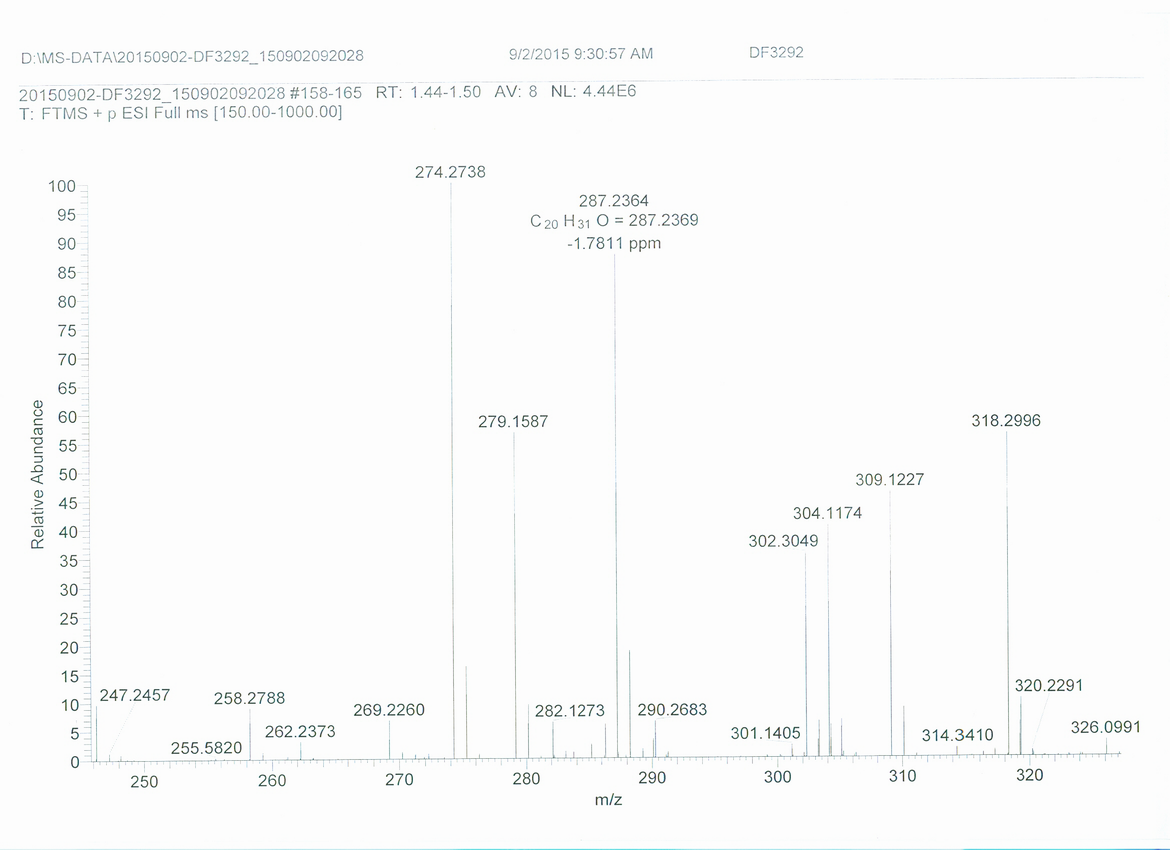
**

**Figure S49.** HRESIMS spectrum of compound **7**

**ECD simulation of Compounds 1 and 2**

**(2*R*,4*S*,5*S*,6*S*,14*S*,15*R*)-1**

**Conf.1**


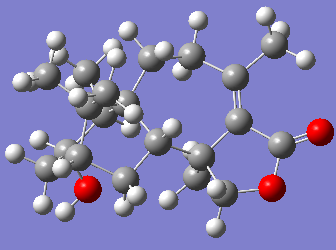


Standard orientation

| Center Number | Atomic Number | Atomic Type | Coordinates (Angstroms) | | |
| --- | --- | --- | --- | --- | --- |
|  |  |  | X | Y | Z |
| 1 | 6 | 0 | -0.025186 | -0.476186 | 0.654129 |
| 2 | 6 | 0 | 1.264791 | -0.924889 | -0.096594 |
| 3 | 6 | 0 | 2.373180 | 0.130057 | -0.094564 |
| 4 | 6 | 0 | 2.274705 | 1.439612 | -0.396068 |
| 5 | 6 | 0 | 0.971821 | 2.072637 | -0.854055 |
| 6 | 6 | 0 | -0.218781 | 2.002577 | 0.128985 |
| 7 | 6 | 0 | -0.978844 | 0.665567 | 0.161886 |
| 8 | 6 | 0 | -1.634062 | 0.346345 | -1.246359 |
| 9 | 6 | 0 | -2.716624 | -0.786492 | -1.282456 |
| 10 | 6 | 0 | -3.287847 | -1.263872 | 0.072080 |
| 11 | 6 | 0 | -2.381079 | -0.937562 | 1.257515 |
| 12 | 6 | 0 | -0.989310 | -1.612001 | 1.087951 |
| 13 | 6 | 0 | -2.152083 | 1.565103 | -2.043510 |
| 14 | 8 | 0 | -2.114355 | -1.881906 | -2.008187 |
| 15 | 6 | 0 | -2.080317 | 0.590954 | 1.301393 |
| 16 | 6 | 0 | -1.576079 | 0.967289 | 2.716084 |
| 17 | 6 | 0 | -3.317649 | 1.477408 | 1.071389 |
| 18 | 6 | 0 | 1.941938 | -2.009547 | 0.786758 |
| 19 | 8 | 0 | 3.359396 | -1.847784 | 0.627493 |
| 20 | 6 | 0 | 3.649360 | -0.566328 | 0.251260 |
| 21 | 8 | 0 | 4.791287 | -0.185457 | 0.205962 |
| 22 | 6 | 0 | 3.431670 | 2.402353 | -0.348021 |
| 23 | 6 | 0 | 1.122676 | -1.510382 | -1.521766 |
| 24 | 1 | 0 | 0.397993 | -0.068067 | 1.574806 |
| 25 | 1 | 0 | 0.692104 | 1.631961 | -1.816278 |
| 26 | 1 | 0 | 1.170716 | 3.126824 | -1.067034 |
| 27 | 1 | 0 | 0.160204 | 2.223306 | 1.130776 |
| 28 | 1 | 0 | -0.908827 | 2.815671 | -0.112265 |
| 29 | 1 | 0 | -0.837670 | -0.061617 | -1.862630 |
| 30 | 1 | 0 | -3.554611 | -0.420514 | -1.887910 |
| 31 | 1 | 0 | -3.449376 | -2.346991 | 0.018569 |
| 32 | 1 | 0 | -4.277895 | -0.825958 | 0.228662 |
| 33 | 1 | 0 | -2.872776 | -1.261911 | 2.181029 |
| 34 | 1 | 0 | -0.656622 | -2.075807 | 2.020375 |
| 35 | 1 | 0 | -1.039524 | -2.408069 | 0.342584 |
| 36 | 1 | 0 | -2.503794 | 1.224314 | -3.021692 |
| 37 | 1 | 0 | -1.373029 | 2.303842 | -2.235103 |
| 38 | 1 | 0 | -2.981245 | 2.087707 | -1.564483 |
| 39 | 1 | 0 | -2.788245 | -2.553296 | -2.171488 |
| 40 | 1 | 0 | -1.260056 | 2.011731 | 2.776898 |
| 41 | 1 | 0 | -2.398447 | 0.844828 | 3.427382 |
| 42 | 1 | 0 | -0.754881 | 0.349558 | 3.080464 |
| 43 | 1 | 0 | -3.817884 | 1.316777 | 0.119287 |
| 44 | 1 | 0 | -3.052264 | 2.537509 | 1.124249 |
| 45 | 1 | 0 | -4.059809 | 1.296638 | 1.855917 |
| 46 | 1 | 0 | 1.690415 | -3.027597 | 0.489477 |
| 47 | 1 | 0 | 1.710046 | -1.879568 | 1.847802 |
| 48 | 1 | 0 | 4.355326 | 1.954411 | 0.003168 |
| 49 | 1 | 0 | 3.607252 | 2.818700 | -1.347896 |
| 50 | 1 | 0 | 3.171697 | 3.253923 | 0.292797 |
| 51 | 1 | 0 | 2.038124 | -2.046737 | -1.788683 |
| 52 | 1 | 0 | 0.986159 | -0.735162 | -2.276722 |
| 53 | 1 | 0 | 0.282610 | -2.201090 | -1.600430 |

HF= -1006.133380

**Conf.2**


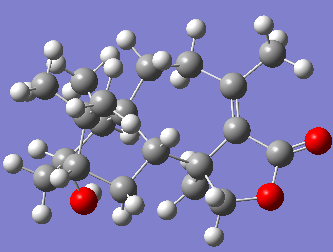


Standard orientation

| Center Number | Atomic Number | Atomic Type | Coordinates (Angstroms) | | |
| --- | --- | --- | --- | --- | --- |
|  |  |  | X | Y | Z |
| 1 | 6 | 0 | -0.029237 | -0.481016 | 0.655386 |
| 2 | 6 | 0 | 1.266310 | -0.922384 | -0.088441 |
| 3 | 6 | 0 | 2.371294 | 0.136304 | -0.093992 |
| 4 | 6 | 0 | 2.270566 | 1.447969 | -0.385873 |
| 5 | 6 | 0 | 0.965896 | 2.088808 | -0.828196 |
| 6 | 6 | 0 | -0.222967 | 2.002464 | 0.155433 |
| 7 | 6 | 0 | -0.981956 | 0.664389 | 0.168458 |
| 8 | 6 | 0 | -1.630655 | 0.363188 | -1.247136 |
| 9 | 6 | 0 | -2.703735 | -0.790053 | -1.303720 |
| 10 | 6 | 0 | -3.278919 | -1.280786 | 0.037185 |
| 11 | 6 | 0 | -2.387199 | -0.955653 | 1.234678 |
| 12 | 6 | 0 | -0.991512 | -1.623372 | 1.075291 |
| 13 | 6 | 0 | -2.163047 | 1.595149 | -2.015636 |
| 14 | 8 | 0 | -2.174694 | -1.962095 | -1.960706 |
| 15 | 6 | 0 | -2.092069 | 0.572819 | 1.299204 |
| 16 | 6 | 0 | -1.596542 | 0.937470 | 2.719980 |
| 17 | 6 | 0 | -3.333644 | 1.454735 | 1.074213 |
| 18 | 6 | 0 | 1.948454 | -1.997984 | 0.802329 |
| 19 | 8 | 0 | 3.364685 | -1.837696 | 0.630845 |
| 20 | 6 | 0 | 3.651410 | -0.559532 | 0.242196 |
| 21 | 8 | 0 | 4.792770 | -0.179927 | 0.179961 |
| 22 | 6 | 0 | 3.429010 | 2.409362 | -0.339867 |
| 23 | 6 | 0 | 1.129015 | -1.524772 | -1.506965 |
| 24 | 1 | 0 | 0.388046 | -0.077418 | 1.580575 |
| 25 | 1 | 0 | 0.684417 | 1.666381 | -1.798504 |
| 26 | 1 | 0 | 1.164905 | 3.146410 | -1.023213 |
| 27 | 1 | 0 | 0.157365 | 2.208300 | 1.159925 |
| 28 | 1 | 0 | -0.913556 | 2.819188 | -0.072002 |
| 29 | 1 | 0 | -0.814953 | -0.010119 | -1.864046 |
| 30 | 1 | 0 | -3.537955 | -0.426693 | -1.915692 |
| 31 | 1 | 0 | -3.421109 | -2.361355 | -0.047923 |
| 32 | 1 | 0 | -4.274937 | -0.856470 | 0.187292 |
| 33 | 1 | 0 | -2.888094 | -1.291365 | 2.149069 |
| 34 | 1 | 0 | -0.663722 | -2.088838 | 2.008761 |
| 35 | 1 | 0 | -1.034389 | -2.417182 | 0.327299 |
| 36 | 1 | 0 | -2.528708 | 1.278650 | -2.998043 |
| 37 | 1 | 0 | -1.389615 | 2.341369 | -2.201142 |
| 38 | 1 | 0 | -2.988879 | 2.103985 | -1.517807 |
| 39 | 1 | 0 | -1.930615 | -1.731553 | -2.865712 |
| 40 | 1 | 0 | -1.292737 | 1.984714 | 2.794779 |
| 41 | 1 | 0 | -2.420223 | 0.797144 | 3.426280 |
| 42 | 1 | 0 | -0.770260 | 0.324479 | 3.080736 |
| 43 | 1 | 0 | -3.838498 | 1.290535 | 0.125329 |
| 44 | 1 | 0 | -3.074713 | 2.516545 | 1.127674 |
| 45 | 1 | 0 | -4.071163 | 1.268182 | 1.861562 |
| 46 | 1 | 0 | 1.694900 | -3.019314 | 0.518700 |
| 47 | 1 | 0 | 1.722485 | -1.855222 | 1.862922 |
| 48 | 1 | 0 | 4.355692 | 1.958049 | -0.001401 |
| 49 | 1 | 0 | 3.595707 | 2.834627 | -1.337525 |
| 50 | 1 | 0 | 3.175406 | 3.255241 | 0.310882 |
| 51 | 1 | 0 | 2.052364 | -2.046050 | -1.775703 |
| 52 | 1 | 0 | 0.972801 | -0.759080 | -2.268559 |
| 53 | 1 | 0 | 0.305114 | -2.238013 | -1.562465 |

HF = -1006.132944

**(2*S*,4*R*,5*R*,6*R*,14*R*,15*S*)-1**

**Conf.1**


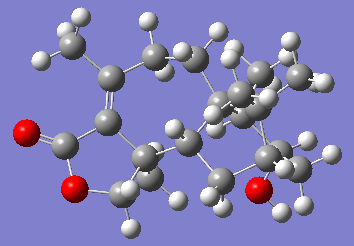


Standard orientation

| Center Number | Atomic Number | Atomic Type | Coordinates (Angstroms) | | |
| --- | --- | --- | --- | --- | --- |
|  |  |  | X | Y | Z |
| 1 | 6 | 0 | 0.025186 | -0.476186 | 0.654129 |
| 2 | 6 | 0 | -1.264791 | -0.924889 | -0.096594 |
| 3 | 6 | 0 | -2.373180 | 0.130057 | -0.094564 |
| 4 | 6 | 0 | -2.274705 | 1.439612 | -0.396068 |
| 5 | 6 | 0 | -0.971821 | 2.072637 | -0.854055 |
| 6 | 6 | 0 | 0.218781 | 2.002577 | 0.128985 |
| 7 | 6 | 0 | 0.978844 | 0.665567 | 0.161886 |
| 8 | 6 | 0 | 1.634062 | 0.346345 | -1.246359 |
| 9 | 6 | 0 | 2.716624 | -0.786492 | -1.282456 |
| 10 | 6 | 0 | 3.287847 | -1.263872 | 0.072080 |
| 11 | 6 | 0 | 2.381079 | -0.937562 | 1.257515 |
| 12 | 6 | 0 | 0.989310 | -1.612001 | 1.087951 |
| 13 | 6 | 0 | 2.152083 | 1.565103 | -2.043510 |
| 14 | 8 | 0 | 2.114355 | -1.881906 | -2.008187 |
| 15 | 6 | 0 | 2.080317 | 0.590954 | 1.301393 |
| 16 | 6 | 0 | 1.576079 | 0.967289 | 2.716084 |
| 17 | 6 | 0 | 3.317649 | 1.477408 | 1.071389 |
| 18 | 6 | 0 | -1.941938 | -2.009547 | 0.786758 |
| 19 | 8 | 0 | -3.359396 | -1.847784 | 0.627493 |
| 20 | 6 | 0 | -3.649360 | -0.566328 | 0.251260 |
| 21 | 8 | 0 | -4.791287 | -0.185457 | 0.205962 |
| 22 | 6 | 0 | -3.431670 | 2.402353 | -0.348021 |
| 23 | 6 | 0 | -1.122676 | -1.510382 | -1.521766 |
| 24 | 1 | 0 | -0.397993 | -0.068067 | 1.574806 |
| 25 | 1 | 0 | -0.692104 | 1.631961 | -1.816278 |
| 26 | 1 | 0 | -1.170716 | 3.126824 | -1.067034 |
| 27 | 1 | 0 | -0.160204 | 2.223306 | 1.130776 |
| 28 | 1 | 0 | 0.908827 | 2.815671 | -0.112265 |
| 29 | 1 | 0 | 0.837670 | -0.061617 | -1.862630 |
| 30 | 1 | 0 | 3.554611 | -0.420514 | -1.887910 |
| 31 | 1 | 0 | 3.449376 | -2.346991 | 0.018569 |
| 32 | 1 | 0 | 4.277895 | -0.825958 | 0.228662 |
| 33 | 1 | 0 | 2.872776 | -1.261911 | 2.181029 |
| 34 | 1 | 0 | 0.656622 | -2.075807 | 2.020375 |
| 35 | 1 | 0 | 1.039524 | -2.408069 | 0.342584 |
| 36 | 1 | 0 | 2.503794 | 1.224314 | -3.021692 |
| 37 | 1 | 0 | 1.373029 | 2.303842 | -2.235103 |
| 38 | 1 | 0 | 2.981245 | 2.087707 | -1.564483 |
| 39 | 1 | 0 | 2.788245 | -2.553296 | -2.171488 |
| 40 | 1 | 0 | 1.260056 | 2.011731 | 2.776898 |
| 41 | 1 | 0 | 2.398447 | 0.844828 | 3.427382 |
| 42 | 1 | 0 | 0.754881 | 0.349558 | 3.080464 |
| 43 | 1 | 0 | 3.817884 | 1.316777 | 0.119287 |
| 44 | 1 | 0 | 3.052264 | 2.537509 | 1.124249 |
| 45 | 1 | 0 | 4.059809 | 1.296638 | 1.855917 |
| 46 | 1 | 0 | -1.690415 | -3.027597 | 0.489477 |
| 47 | 1 | 0 | -1.710046 | -1.879568 | 1.847802 |
| 48 | 1 | 0 | -4.355326 | 1.954411 | 0.003168 |
| 49 | 1 | 0 | -3.171697 | 3.253923 | 0.292797 |
| 50 | 1 | 0 | -3.607252 | 2.818700 | -1.347896 |
| 51 | 1 | 0 | -2.038124 | -2.046737 | -1.788683 |
| 52 | 1 | 0 | -0.986159 | -0.735162 | -2.276722 |
| 53 | 1 | 0 | -0.282610 | -2.201090 | -1.600430 |

HF = -1006.133380

**Conf.2**


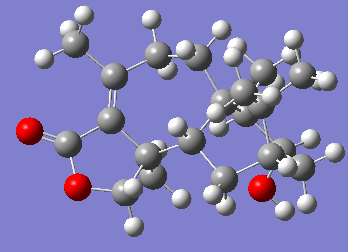


Standard orientation

| Center Number | Atomic Number | Atomic Type | Coordinates (Angstroms) | | |
| --- | --- | --- | --- | --- | --- |
|  |  |  | X | Y | Z |
| 1 | 6 | 0 | 0.029237 | -0.481016 | 0.655386 |
| 2 | 6 | 0 | -1.266310 | -0.922384 | -0.088441 |
| 3 | 6 | 0 | -2.371294 | 0.136304 | -0.093992 |
| 4 | 6 | 0 | -2.270566 | 1.447969 | -0.385873 |
| 5 | 6 | 0 | -0.965896 | 2.088808 | -0.828196 |
| 6 | 6 | 0 | 0.222967 | 2.002464 | 0.155433 |
| 7 | 6 | 0 | 0.981956 | 0.664389 | 0.168458 |
| 8 | 6 | 0 | 1.630655 | 0.363188 | -1.247136 |
| 9 | 6 | 0 | 2.703735 | -0.790053 | -1.303720 |
| 10 | 6 | 0 | 3.278919 | -1.280786 | 0.037185 |
| 11 | 6 | 0 | 2.387199 | -0.955653 | 1.234678 |
| 12 | 6 | 0 | 0.991512 | -1.623372 | 1.075291 |
| 13 | 6 | 0 | 2.163047 | 1.595149 | -2.015636 |
| 14 | 8 | 0 | 2.174694 | -1.962095 | -1.960706 |
| 15 | 6 | 0 | 2.092069 | 0.572819 | 1.299204 |
| 16 | 6 | 0 | 1.596542 | 0.937470 | 2.719980 |
| 17 | 6 | 0 | 3.333644 | 1.454735 | 1.074213 |
| 18 | 6 | 0 | -1.948454 | -1.997984 | 0.802329 |
| 19 | 8 | 0 | -3.364685 | -1.837696 | 0.630845 |
| 20 | 6 | 0 | -3.651410 | -0.559532 | 0.242196 |
| 21 | 8 | 0 | -4.792770 | -0.179927 | 0.179961 |
| 22 | 6 | 0 | -3.429010 | 2.409362 | -0.339867 |
| 23 | 6 | 0 | -1.129015 | -1.524772 | -1.506965 |
| 24 | 1 | 0 | -0.388046 | -0.077418 | 1.580575 |
| 25 | 1 | 0 | -0.684417 | 1.666381 | -1.798504 |
| 26 | 1 | 0 | -1.164905 | 3.146410 | -1.023213 |
| 27 | 1 | 0 | -0.157365 | 2.208300 | 1.159925 |
| 28 | 1 | 0 | 0.913556 | 2.819188 | -0.072002 |
| 29 | 1 | 0 | 0.814953 | -0.010119 | -1.864046 |
| 30 | 1 | 0 | 3.537955 | -0.426693 | -1.915692 |
| 31 | 1 | 0 | 3.421109 | -2.361355 | -0.047923 |
| 32 | 1 | 0 | 4.274937 | -0.856470 | 0.187292 |
| 33 | 1 | 0 | 2.888094 | -1.291365 | 2.149069 |
| 34 | 1 | 0 | 0.663722 | -2.088838 | 2.008761 |
| 35 | 1 | 0 | 1.034389 | -2.417182 | 0.327299 |
| 36 | 1 | 0 | 2.528708 | 1.278650 | -2.998043 |
| 37 | 1 | 0 | 1.389615 | 2.341369 | -2.201142 |
| 38 | 1 | 0 | 2.988879 | 2.103985 | -1.517807 |
| 39 | 1 | 0 | 1.930615 | -1.731553 | -2.865712 |
| 40 | 1 | 0 | 1.292737 | 1.984714 | 2.794779 |
| 41 | 1 | 0 | 2.420223 | 0.797144 | 3.426280 |
| 42 | 1 | 0 | 0.770260 | 0.324479 | 3.080736 |
| 43 | 1 | 0 | 3.838498 | 1.290535 | 0.125329 |
| 44 | 1 | 0 | 3.074713 | 2.516545 | 1.127674 |
| 45 | 1 | 0 | 4.071163 | 1.268182 | 1.861562 |
| 46 | 1 | 0 | -1.694900 | -3.019314 | 0.518700 |
| 47 | 1 | 0 | -1.722485 | -1.855222 | 1.862922 |
| 48 | 1 | 0 | -4.355692 | 1.958049 | -0.001401 |
| 49 | 1 | 0 | -3.175406 | 3.255241 | 0.310882 |
| 50 | 1 | 0 | -3.595707 | 2.834627 | -1.337525 |
| 51 | 1 | 0 | -2.052364 | -2.046050 | -1.775703 |
| 52 | 1 | 0 | -0.972801 | -0.759080 | -2.268559 |
| 53 | 1 | 0 | -0.305114 | -2.238013 | -1.562465 |

HF = -1006.132944

**(2*R*,4*S*,5*S*,6*S*,13*S*,14*R*)-2**

**Conf.1**


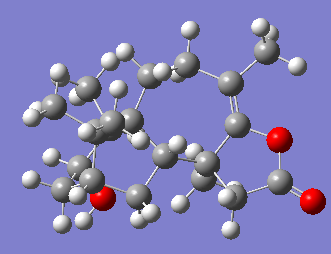


Standard orientation

| Center Number | Atomic Number | Atomic Type | Coordinates (Angstroms) | | |
| --- | --- | --- | --- | --- | --- |
|  |  |  | X | Y | Z |
| 1 | 6 | 0 | 0.038551 | -0.469992 | 0.593103 |
| 2 | 6 | 0 | -1.036517 | 0.615972 | 0.235229 |
| 3 | 6 | 0 | -1.682901 | 0.395859 | -1.195400 |
| 4 | 6 | 0 | -2.624714 | -0.845369 | -1.359253 |
| 5 | 6 | 0 | -3.126003 | -1.527224 | -0.066163 |
| 6 | 6 | 0 | -2.244639 | -1.243093 | 1.149123 |
| 7 | 6 | 0 | -0.792090 | -1.741128 | 0.907691 |
| 8 | 6 | 0 | 1.353273 | -0.710647 | -0.207715 |
| 9 | 6 | 0 | 2.300355 | 0.488252 | -0.153132 |
| 10 | 6 | 0 | 2.088475 | 1.795991 | -0.308022 |
| 11 | 6 | 0 | 0.717030 | 2.373697 | -0.614612 |
| 12 | 6 | 0 | -0.429126 | 2.025566 | 0.359256 |
| 13 | 6 | 0 | -2.105481 | 0.293191 | 1.366627 |
| 14 | 6 | 0 | -1.617261 | 0.558883 | 2.811812 |
| 15 | 6 | 0 | -3.435158 | 1.060978 | 1.253160 |
| 16 | 6 | 0 | -2.361516 | 1.633033 | -1.827359 |
| 17 | 6 | 0 | 3.219028 | 2.792678 | -0.251871 |
| 18 | 6 | 0 | 2.200960 | -1.765257 | 0.552639 |
| 19 | 6 | 0 | 3.630097 | -1.291860 | 0.408210 |
| 20 | 8 | 0 | 3.613874 | 0.026229 | 0.048470 |
| 21 | 8 | 0 | -1.906336 | -1.781030 | -2.194872 |
| 22 | 8 | 0 | 4.651846 | -1.891579 | 0.582734 |
| 23 | 6 | 0 | 1.240738 | -1.173589 | -1.680444 |
| 24 | 1 | 0 | -0.859743 | 0.166938 | -1.867037 |
| 25 | 1 | 0 | -3.504290 | -0.517283 | -1.926209 |
| 26 | 1 | 0 | -3.176425 | -2.608459 | -0.241031 |
| 27 | 1 | 0 | -4.154033 | -1.214599 | 0.138863 |
| 28 | 1 | 0 | -2.689666 | -1.720094 | 2.029220 |
| 29 | 1 | 0 | -0.397372 | -2.259151 | 1.785671 |
| 30 | 1 | 0 | -0.763706 | -2.456694 | 0.084016 |
| 31 | 1 | 0 | 0.437412 | 2.117676 | -1.642774 |
| 32 | 1 | 0 | 0.821293 | 3.463363 | -0.616693 |
| 33 | 1 | 0 | -0.045603 | 2.156305 | 1.374929 |
| 34 | 1 | 0 | -1.218123 | 2.774151 | 0.241197 |
| 35 | 1 | 0 | -1.416761 | 1.618073 | 2.990376 |
| 36 | 1 | 0 | -2.406668 | 0.265379 | 3.510389 |
| 37 | 1 | 0 | -0.726374 | -0.000836 | 3.097611 |
| 38 | 1 | 0 | -3.281620 | 2.132479 | 1.411425 |
| 39 | 1 | 0 | -3.947227 | 0.944485 | 0.300936 |
| 40 | 1 | 0 | -4.128602 | 0.721873 | 2.029813 |
| 41 | 1 | 0 | -3.237350 | 1.984051 | -1.281160 |
| 42 | 1 | 0 | -2.689116 | 1.378538 | -2.839623 |
| 43 | 1 | 0 | -1.681441 | 2.479259 | -1.924312 |
| 44 | 1 | 0 | 3.307020 | 3.329797 | -1.204197 |
| 45 | 1 | 0 | 3.025445 | 3.551748 | 0.515548 |
| 46 | 1 | 0 | 4.179064 | 2.326746 | -0.037596 |
| 47 | 1 | 0 | 2.091612 | -2.784283 | 0.181859 |
| 48 | 1 | 0 | 1.976938 | -1.780374 | 1.624773 |
| 49 | 1 | 0 | -2.501946 | -2.501832 | -2.433979 |
| 50 | 1 | 0 | 2.211884 | -1.539209 | -2.028822 |
| 51 | 1 | 0 | 0.956600 | -0.361157 | -2.348490 |
| 52 | 1 | 0 | 0.510219 | -1.973244 | -1.801058 |
| 53 | 1 | 0 | 0.434907 | -0.118259 | 1.548429 |

HF = -1006.136021

**Conf.2**


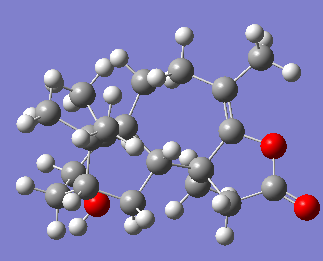


Standard orientation

| Center Number | Atomic Number | Atomic Type | Coordinates (Angstroms) | | |
| --- | --- | --- | --- | --- | --- |
|  |  |  | X | Y | Z |
| 1 | 6 | 0 | 0.038449 | -0.469972 | 0.593390 |
| 2 | 6 | 0 | -1.036550 | 0.615995 | 0.235376 |
| 3 | 6 | 0 | -1.682417 | 0.395866 | -1.195488 |
| 4 | 6 | 0 | -2.624117 | -0.845393 | -1.359652 |
| 5 | 6 | 0 | -3.125890 | -1.527162 | -0.066725 |
| 6 | 6 | 0 | -2.244896 | -1.243116 | 1.148855 |
| 7 | 6 | 0 | -0.792232 | -1.741105 | 0.907932 |
| 8 | 6 | 0 | 1.353145 | -0.710614 | -0.207445 |
| 9 | 6 | 0 | 2.300247 | 0.488286 | -0.152932 |
| 10 | 6 | 0 | 2.088398 | 1.796025 | -0.307851 |
| 11 | 6 | 0 | 0.716953 | 2.373832 | -0.614267 |
| 12 | 6 | 0 | -0.429153 | 2.025576 | 0.359607 |
| 13 | 6 | 0 | -2.105843 | 0.293148 | 1.366488 |
| 14 | 6 | 0 | -1.618094 | 0.558756 | 2.811861 |
| 15 | 6 | 0 | -3.435528 | 1.060884 | 1.252739 |
| 16 | 6 | 0 | -2.360666 | 1.633045 | -1.827844 |
| 17 | 6 | 0 | 3.219009 | 2.792665 | -0.251861 |
| 18 | 6 | 0 | 2.200877 | -1.765255 | 0.552826 |
| 19 | 6 | 0 | 3.630015 | -1.291864 | 0.408310 |
| 20 | 8 | 0 | 3.613779 | 0.026225 | 0.048573 |
| 21 | 8 | 0 | -1.905395 | -1.781124 | -2.194892 |
| 22 | 8 | 0 | 4.651756 | -1.891604 | 0.582746 |
| 23 | 6 | 0 | 1.240511 | -1.173518 | -1.680176 |
| 24 | 1 | 0 | -0.858993 | 0.166910 | -1.866799 |
| 25 | 1 | 0 | -3.503484 | -0.517387 | -1.926965 |
| 26 | 1 | 0 | -3.176395 | -2.608379 | -0.241657 |
| 27 | 1 | 0 | -4.153964 | -1.214439 | 0.137963 |
| 28 | 1 | 0 | -2.690195 | -1.720181 | 2.028781 |
| 29 | 1 | 0 | -0.397737 | -2.258975 | 1.786111 |
| 30 | 1 | 0 | -0.763572 | -2.456786 | 0.084371 |
| 31 | 1 | 0 | 0.437259 | 2.117991 | -1.642461 |
| 32 | 1 | 0 | 0.821232 | 3.463500 | -0.616193 |
| 33 | 1 | 0 | -0.045562 | 2.156156 | 1.375275 |
| 34 | 1 | 0 | -1.218136 | 2.774202 | 0.241724 |
| 35 | 1 | 0 | -1.417651 | 1.617934 | 2.990558 |
| 36 | 1 | 0 | -2.407746 | 0.265225 | 3.510157 |
| 37 | 1 | 0 | -0.727316 | -0.000993 | 3.097948 |
| 38 | 1 | 0 | -3.282065 | 2.132397 | 1.411014 |
| 39 | 1 | 0 | -3.947423 | 0.944346 | 0.300431 |
| 40 | 1 | 0 | -4.129105 | 0.721766 | 2.029273 |
| 41 | 1 | 0 | -3.236692 | 1.984253 | -1.282080 |
| 42 | 1 | 0 | -2.687875 | 1.378466 | -2.840218 |
| 43 | 1 | 0 | -1.680450 | 2.479182 | -1.924648 |
| 44 | 1 | 0 | 3.306846 | 3.329813 | -1.204187 |
| 45 | 1 | 0 | 3.025614 | 3.551721 | 0.515624 |
| 46 | 1 | 0 | 4.179069 | 2.326685 | -0.037782 |
| 47 | 1 | 0 | 2.091451 | -2.784276 | 0.182051 |
| 48 | 1 | 0 | 1.976896 | -1.780383 | 1.624975 |
| 49 | 1 | 0 | -2.501099 | -2.501587 | -2.434702 |
| 50 | 1 | 0 | 2.211713 | -1.538827 | -2.028727 |
| 51 | 1 | 0 | 0.955981 | -0.361157 | -2.348146 |
| 52 | 1 | 0 | 0.510212 | -1.973391 | -1.800678 |
| 53 | 1 | 0 | 0.434767 | -0.118237 | 1.548722 |

HF = -1006.136020

**Conf.3**


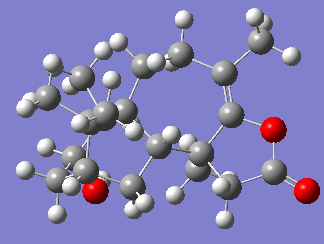


Standard orientation

| Center Number | Atomic Number | Atomic Type | Coordinates (Angstroms) | | |
| --- | --- | --- | --- | --- | --- |
|  |  |  | X | Y | Z |
| 1 | 6 | 0 | 0.034382 | -0.474752 | 0.594704 |
| 2 | 6 | 0 | -1.039594 | 0.613931 | 0.240119 |
| 3 | 6 | 0 | -1.680464 | 0.409150 | -1.195381 |
| 4 | 6 | 0 | -2.610473 | -0.849428 | -1.378707 |
| 5 | 6 | 0 | -3.116728 | -1.538398 | -0.098951 |
| 6 | 6 | 0 | -2.250443 | -1.256059 | 1.127861 |
| 7 | 6 | 0 | -0.794574 | -1.749437 | 0.899838 |
| 8 | 6 | 0 | 1.353747 | -0.709713 | -0.198761 |
| 9 | 6 | 0 | 2.295843 | 0.493412 | -0.153345 |
| 10 | 6 | 0 | 2.081779 | 1.801824 | -0.301519 |
| 11 | 6 | 0 | 0.709158 | 2.386320 | -0.589472 |
| 12 | 6 | 0 | -0.433979 | 2.022478 | 0.381724 |
| 13 | 6 | 0 | -2.115752 | 0.277541 | 1.361680 |
| 14 | 6 | 0 | -1.633465 | 0.533447 | 2.810858 |
| 15 | 6 | 0 | -3.447928 | 1.041453 | 1.251097 |
| 16 | 6 | 0 | -2.373904 | 1.651032 | -1.803057 |
| 17 | 6 | 0 | 3.214238 | 2.797132 | -0.253631 |
| 18 | 6 | 0 | 2.206508 | -1.753794 | 0.570780 |
| 19 | 6 | 0 | 3.633878 | -1.280816 | 0.409078 |
| 20 | 8 | 0 | 3.612428 | 0.033874 | 0.035084 |
| 21 | 8 | 0 | -1.953315 | -1.879491 | -2.148646 |
| 22 | 8 | 0 | 4.657998 | -1.877199 | 0.579127 |
| 23 | 6 | 0 | 1.248984 | -1.190633 | -1.666547 |
| 24 | 1 | 0 | -0.841670 | 0.214843 | -1.862350 |
| 25 | 1 | 0 | -3.484815 | -0.524999 | -1.955677 |
| 26 | 1 | 0 | -3.150225 | -2.612121 | -0.301674 |
| 27 | 1 | 0 | -4.148900 | -1.236557 | 0.096674 |
| 28 | 1 | 0 | -2.704258 | -1.741652 | 1.998636 |
| 29 | 1 | 0 | -0.404967 | -2.265256 | 1.781512 |
| 30 | 1 | 0 | -0.759502 | -2.466374 | 0.077893 |
| 31 | 1 | 0 | 0.424997 | 2.152026 | -1.621883 |
| 32 | 1 | 0 | 0.815382 | 3.475632 | -0.571117 |
| 33 | 1 | 0 | -0.049245 | 2.140146 | 1.398483 |
| 34 | 1 | 0 | -1.224043 | 2.771841 | 0.275604 |
| 35 | 1 | 0 | -1.441993 | 1.592567 | 3.000522 |
| 36 | 1 | 0 | -2.422516 | 0.226530 | 3.503860 |
| 37 | 1 | 0 | -0.739199 | -0.022012 | 3.094318 |
| 38 | 1 | 0 | -3.298500 | 2.114451 | 1.403924 |
| 39 | 1 | 0 | -3.966057 | 0.916202 | 0.303398 |
| 40 | 1 | 0 | -4.135348 | 0.702670 | 2.033003 |
| 41 | 1 | 0 | -3.246154 | 1.988008 | -1.243737 |
| 42 | 1 | 0 | -2.715449 | 1.414583 | -2.816191 |
| 43 | 1 | 0 | -1.700653 | 2.503372 | -1.894646 |
| 44 | 1 | 0 | 3.287808 | 3.342879 | -1.202250 |
| 45 | 1 | 0 | 3.032640 | 3.549272 | 0.523412 |
| 46 | 1 | 0 | 4.177104 | 2.329019 | -0.058159 |
| 47 | 1 | 0 | 2.094917 | -2.778240 | 0.216112 |
| 48 | 1 | 0 | 1.987774 | -1.752606 | 1.644049 |
| 49 | 1 | 0 | -1.750156 | -1.533394 | -3.026385 |
| 50 | 1 | 0 | 2.225263 | -1.542495 | -2.014019 |
| 51 | 1 | 0 | 0.951226 | -0.388378 | -2.341984 |
| 52 | 1 | 0 | 0.536395 | -2.009628 | -1.768987 |
| 53 | 1 | 0 | 0.425982 | -0.125946 | 1.552930 |

HF = -1006.135589

**Conf.4**


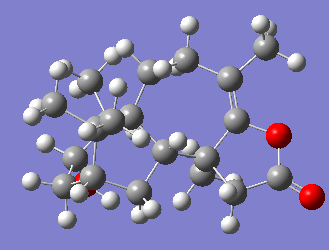


Standard orientation

| Center Number | Atomic Number | Atomic Type | Coordinates (Angstroms) | | |
| --- | --- | --- | --- | --- | --- |
|  |  |  | X | Y | Z |
| 1 | 6 | 0 | 0.067013 | -0.494031 | 0.595855 |
| 2 | 6 | 0 | -1.018045 | 0.599551 | 0.282558 |
| 3 | 6 | 0 | -1.686954 | 0.430394 | -1.144357 |
| 4 | 6 | 0 | -2.755597 | -0.716196 | -1.316601 |
| 5 | 6 | 0 | -3.086991 | -1.580906 | -0.072427 |
| 6 | 6 | 0 | -2.204735 | -1.300962 | 1.145011 |
| 7 | 6 | 0 | -0.750073 | -1.775627 | 0.887513 |
| 8 | 6 | 0 | 1.372849 | -0.719620 | -0.222115 |
| 9 | 6 | 0 | 2.320325 | 0.478477 | -0.172754 |
| 10 | 6 | 0 | 2.106077 | 1.791069 | -0.269649 |
| 11 | 6 | 0 | 0.730986 | 2.385432 | -0.519625 |
| 12 | 6 | 0 | -0.406401 | 2.002557 | 0.452582 |
| 13 | 6 | 0 | -2.079646 | 0.230443 | 1.405950 |
| 14 | 6 | 0 | -1.591629 | 0.464900 | 2.855882 |
| 15 | 6 | 0 | -3.418823 | 0.984008 | 1.308755 |
| 16 | 6 | 0 | -2.291082 | 1.713244 | -1.761163 |
| 17 | 6 | 0 | 3.242081 | 2.781444 | -0.213013 |
| 18 | 6 | 0 | 2.238292 | -1.777531 | 0.515264 |
| 19 | 6 | 0 | 3.664427 | -1.310951 | 0.321917 |
| 20 | 8 | 0 | 3.639964 | 0.008508 | -0.031402 |
| 21 | 8 | 0 | -2.442835 | -1.524256 | -2.464667 |
| 22 | 8 | 0 | 4.687561 | -1.918009 | 0.453807 |
| 23 | 6 | 0 | 1.242377 | -1.179855 | -1.693989 |
| 24 | 1 | 0 | -0.887690 | 0.149579 | -1.826038 |
| 25 | 1 | 0 | -3.684415 | -0.240014 | -1.632503 |
| 26 | 1 | 0 | -3.009781 | -2.635980 | -0.353644 |
| 27 | 1 | 0 | -4.137659 | -1.430911 | 0.192098 |
| 28 | 1 | 0 | -2.635447 | -1.807506 | 2.015327 |
| 29 | 1 | 0 | -0.342500 | -2.312735 | 1.747978 |
| 30 | 1 | 0 | -0.719181 | -2.485565 | 0.054986 |
| 31 | 1 | 0 | 0.437592 | 2.175446 | -1.553257 |
| 32 | 1 | 0 | 0.840144 | 3.473664 | -0.476362 |
| 33 | 1 | 0 | -0.015607 | 2.098203 | 1.469583 |
| 34 | 1 | 0 | -1.194382 | 2.756095 | 0.364865 |
| 35 | 1 | 0 | -1.412816 | 1.522966 | 3.062504 |
| 36 | 1 | 0 | -2.370544 | 0.134677 | 3.549674 |
| 37 | 1 | 0 | -0.686469 | -0.082369 | 3.122273 |
| 38 | 1 | 0 | -3.279481 | 2.052956 | 1.495306 |
| 39 | 1 | 0 | -3.926683 | 0.885739 | 0.351709 |
| 40 | 1 | 0 | -4.109712 | 0.616435 | 2.074286 |
| 41 | 1 | 0 | -3.123846 | 2.112650 | -1.179626 |
| 42 | 1 | 0 | -2.672849 | 1.474564 | -2.757603 |
| 43 | 1 | 0 | -1.567176 | 2.518013 | -1.885587 |
| 44 | 1 | 0 | 3.299535 | 3.353968 | -1.146674 |
| 45 | 1 | 0 | 3.074821 | 3.510870 | 0.588279 |
| 46 | 1 | 0 | 4.207232 | 2.306244 | -0.048313 |
| 47 | 1 | 0 | 2.113506 | -2.798628 | 0.154961 |
| 48 | 1 | 0 | 2.046023 | -1.783956 | 1.593515 |
| 49 | 1 | 0 | -1.642851 | -2.035112 | -2.289648 |
| 50 | 1 | 0 | 2.222014 | -1.472634 | -2.083080 |
| 51 | 1 | 0 | 0.865793 | -0.398025 | -2.351036 |
| 52 | 1 | 0 | 0.594707 | -2.057258 | -1.784708 |
| 53 | 1 | 0 | 0.473509 | -0.166358 | 1.555270 |

HF = -1006.132304

**Conf.5**


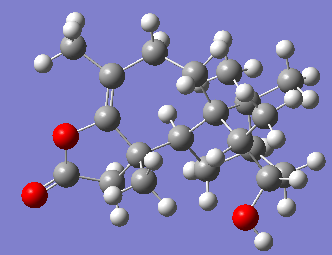


Standard orientation

| Center Number | Atomic Number | Atomic Type | Coordinates (Angstroms) | | |
| --- | --- | --- | --- | --- | --- |
|  |  |  | X | Y | Z |
| 1 | 6 | 0 | -0.035336 | -0.192375 | -0.599398 |
| 2 | 6 | 0 | 1.050494 | 0.601174 | 0.199088 |
| 3 | 6 | 0 | 1.815874 | -0.283965 | 1.279769 |
| 4 | 6 | 0 | 2.716642 | -1.442083 | 0.745082 |
| 5 | 6 | 0 | 3.166213 | -1.337682 | -0.727516 |
| 6 | 6 | 0 | 2.197311 | -0.519151 | -1.580796 |
| 7 | 6 | 0 | 0.770313 | -1.143453 | -1.513722 |
| 8 | 6 | 0 | -1.325485 | -0.721555 | 0.080266 |
| 9 | 6 | 0 | -2.320379 | 0.417712 | 0.287524 |
| 10 | 6 | 0 | -2.178768 | 1.741728 | 0.382734 |
| 11 | 6 | 0 | -0.848285 | 2.477386 | 0.360608 |
| 12 | 6 | 0 | 0.403194 | 1.796260 | 0.943086 |
| 13 | 6 | 0 | 2.041870 | 0.926620 | -0.999449 |
| 14 | 6 | 0 | 1.470837 | 1.862779 | -2.092257 |
| 15 | 6 | 0 | 3.376610 | 1.581346 | -0.598247 |
| 16 | 6 | 0 | 2.585182 | 0.492187 | 2.377156 |
| 17 | 6 | 0 | -3.380385 | 2.652753 | 0.457773 |
| 18 | 6 | 0 | -2.169861 | -1.582293 | -0.895878 |
| 19 | 6 | 0 | -3.596054 | -1.319925 | -0.451964 |
| 20 | 8 | 0 | -3.617729 | -0.136966 | 0.240457 |
| 21 | 8 | 0 | 1.976281 | -2.662722 | 0.981988 |
| 22 | 8 | 0 | -4.585617 | -1.967399 | -0.636581 |
| 23 | 6 | 0 | -1.186718 | -1.532908 | 1.390600 |
| 24 | 1 | 0 | 1.039451 | -0.807287 | 1.828408 |
| 25 | 1 | 0 | 3.613964 | -1.483811 | 1.374134 |
| 26 | 1 | 0 | 3.255303 | -2.350859 | -1.138143 |
| 27 | 1 | 0 | 4.172138 | -0.912051 | -0.778599 |
| 28 | 1 | 0 | 2.570738 | -0.480534 | -2.609799 |
| 29 | 1 | 0 | 0.316230 | -1.208771 | -2.506135 |
| 30 | 1 | 0 | 0.811842 | -2.157914 | -1.116098 |
| 31 | 1 | 0 | -0.992648 | 3.395114 | 0.943824 |
| 32 | 1 | 0 | -0.670757 | 2.833234 | -0.661147 |
| 33 | 1 | 0 | 1.158320 | 2.572895 | 1.108144 |
| 34 | 1 | 0 | 0.118681 | 1.458860 | 1.944601 |
| 35 | 1 | 0 | 1.274228 | 2.864496 | -1.701866 |
| 36 | 1 | 0 | 2.215027 | 1.975170 | -2.886778 |
| 37 | 1 | 0 | 0.559803 | 1.504437 | -2.571846 |
| 38 | 1 | 0 | 3.208711 | 2.571644 | -0.164247 |
| 39 | 1 | 0 | 3.967783 | 1.012139 | 0.113305 |
| 40 | 1 | 0 | 4.002000 | 1.725833 | -1.485237 |
| 41 | 1 | 0 | 3.464953 | 1.025317 | 2.017341 |
| 42 | 1 | 0 | 2.927389 | -0.221379 | 3.132637 |
| 43 | 1 | 0 | 1.958034 | 1.216097 | 2.896354 |
| 44 | 1 | 0 | -3.417025 | 3.167075 | 1.425505 |
| 45 | 1 | 0 | -3.314149 | 3.435121 | -0.307663 |
| 46 | 1 | 0 | -4.319899 | 2.120110 | 0.323438 |
| 47 | 1 | 0 | -1.944447 | -2.648253 | -0.875919 |
| 48 | 1 | 0 | -2.077355 | -1.235597 | -1.931314 |
| 49 | 1 | 0 | 2.543642 | -3.415383 | 0.774587 |
| 50 | 1 | 0 | -2.147437 | -1.981013 | 1.661226 |
| 51 | 1 | 0 | -0.892535 | -0.903704 | 2.230671 |
| 52 | 1 | 0 | -0.458186 | -2.337443 | 1.283167 |
| 53 | 1 | 0 | -0.446895 | 0.555814 | -1.276204 |

HF = 1006.128508
